# Supplementary material for: The main oxidative inactivation pathway of the plant hormone auxin
Source: Nat Commun. 2021 Nov 22;12:6752. doi: 10.1038/s41467-021-27020-1 (PMC8608799; doi:10.1038/s41467-021-27020-1)
Supplement: Supplementary file 1 — Supplementary Information [file 41467_2021_27020_MOESM1_ESM.pdf]

Supplementary Information

The main oxidative inactivation pathway of plant hormone auxin

Ken-ichiro Hayashi[1]*, Kazushi Arai[1], Yuki Aoi[2], Yuka Tanaka[1], Hayao Hira[3], Ruipan Guo[4], Yun Hu[4], Chennan Ge[4], Yunde Zhao[4], Hiroyuki Kasahara[5,6], Kosuke Fukui[1]

Correspondence to: Ken-ichiro Hayashi (hayashi@dbc.ous.ac.jp)

This PDF file includes:

Supplementary Figs. 1 to 20
Supplementary tables 1 and 2.
Supplementary Methods
Supplementary Reference 1 to 14

Supplementary Figures 1-20

Supplementary Fig. 1. Schematic representation of the IAA inactivation pathways and the mutant lines used in this study

Supplementary Fig. 2. GH3 enzymes are involved in major IAA inactivation pathways.

Supplementary Fig. 3. Endogenous amounts of IAA and its metabolites in IAA-inactivating enzyme mutants, *YUC2* overexpressing line, and WT plants treated with a GH3 inhibitor KKI.

Supplementary Fig. 4. AtDAO1 catalyzes the oxidation of IAA-amino acid conjugates to yield corresponding oxIAA-amino acid conjugates.

Supplementary Fig. 5. Docking study of IAA, IAA-Asp, and IAA-Glu with AtDAO1.

Supplementary Fig. 6. OsDAO functions as an IAA-amino acid conjugate oxidase to produce oxIAA-amino acids.

Supplementary Fig. 7. IAA-Asp functions as a storage form of IAA.

Supplementary Fig. 8. IAA-amino acid diesters showed auxin activity in *Brachypodium distachyon* and rice (*Oryza sativa*).

Supplementary Fig. 9. AtDAO1 modulates auxin activity derived from IAA-Asp and IAA-Glu.

Supplementary Fig. 10. Endogenous levels of IAA-amino acid conjugates in wild-type and *dao1-1* and the effects of IAA-amino acid conjugates on *dao1* mutants.

Supplementary Fig. 11. Rice OsDAO complements the hyper-sensitivity of *dao1-1* to IAA-amino acid diesters and the *Atdao2-1* mutants showed the same sensitivity as WT to IAA-Asp-DM

Supplementary Fig. 12. Endogenous amounts of IAA and its metabolites in *dao1-1* and *GFP-AtDAO1*-overexpressing plants.

Supplementary Fig. 13. Endogenous amounts of IAA metabolites in rice (*Oryza sativa*) and *Brachypodium distachyon.*

Supplementary Fig. 14. ILR1/ILL enzymes are responsible for the conversion of IAA-Asp and IAA-Glu to IAA.

Supplementary Fig. 15. *ILR1*, *ILL2*, *IAR3,* and *ILL3* are required for the conversion of IAA-Asp and IAA-Glu to IAA.

Supplementary Fig. 16. Expression patterns of *ILR1*, *ILL2* and *IAR3*.

Supplementary Fig. 17. The responses of the *dao1* mutant to IAA-Asp and IAA-Glu esters depend on ILR1.

Supplementary Fig. 18. The *ilr1 iar3* loss-of-function mutation restored *dao1-1* phenotypes.

Supplementary Fig. 19. GST-ILR1 hydrolyzed both oxIAA-amino acid conjugates and IAA-amino acid conjugates.

Supplementary Fig. 20. Endogenous amounts of IAA-Asp and dioxIAA in *ilr1* mutant and the complementation line.

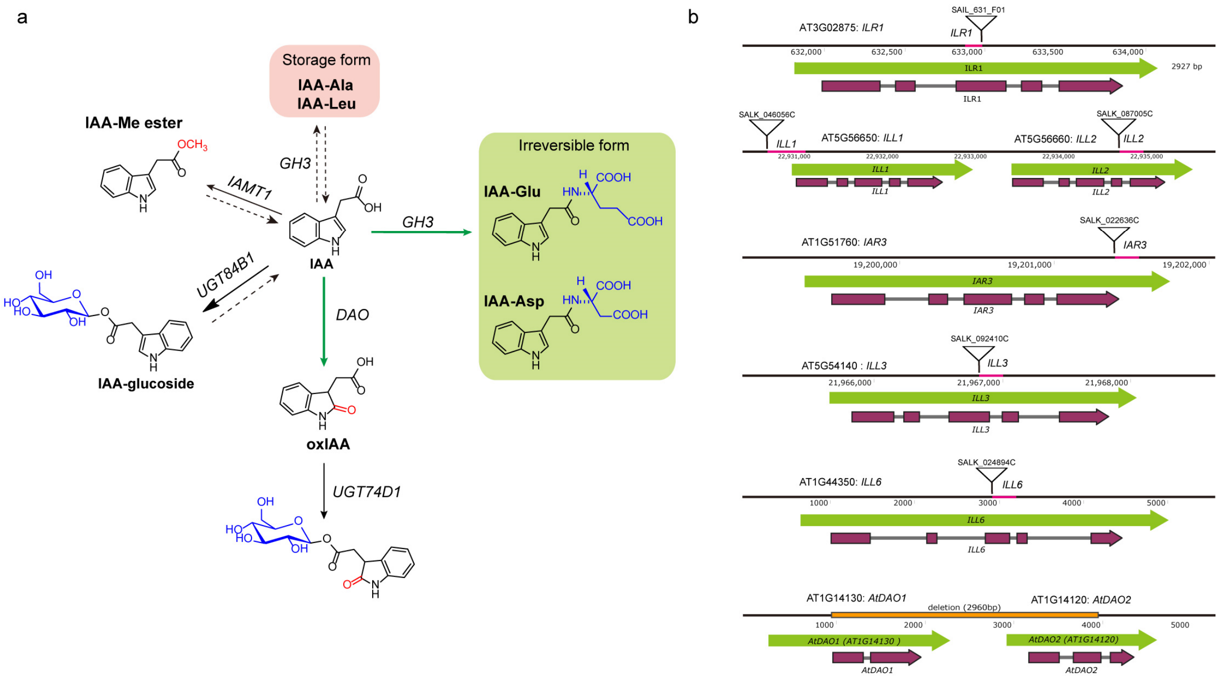

Supplementary Fig. 1. Schematic representation of the IAA inactivation pathways and the mutant lines used in this study.

(a) Chemical structures of IAA metabolites and enzymes in IAA catabolic pathways. (b) Schematic representation of homozygous T-DNA insertion lines and CRISPR-Cas9 *dao1 dao2* line. The magenta boxes indicate exons, the gray lines indicate introns, and the green boxes indicate a genomic sequence. The insertion sites are indicated by triangles. The red line indicates the genome sequence outside the T-DNA left border.

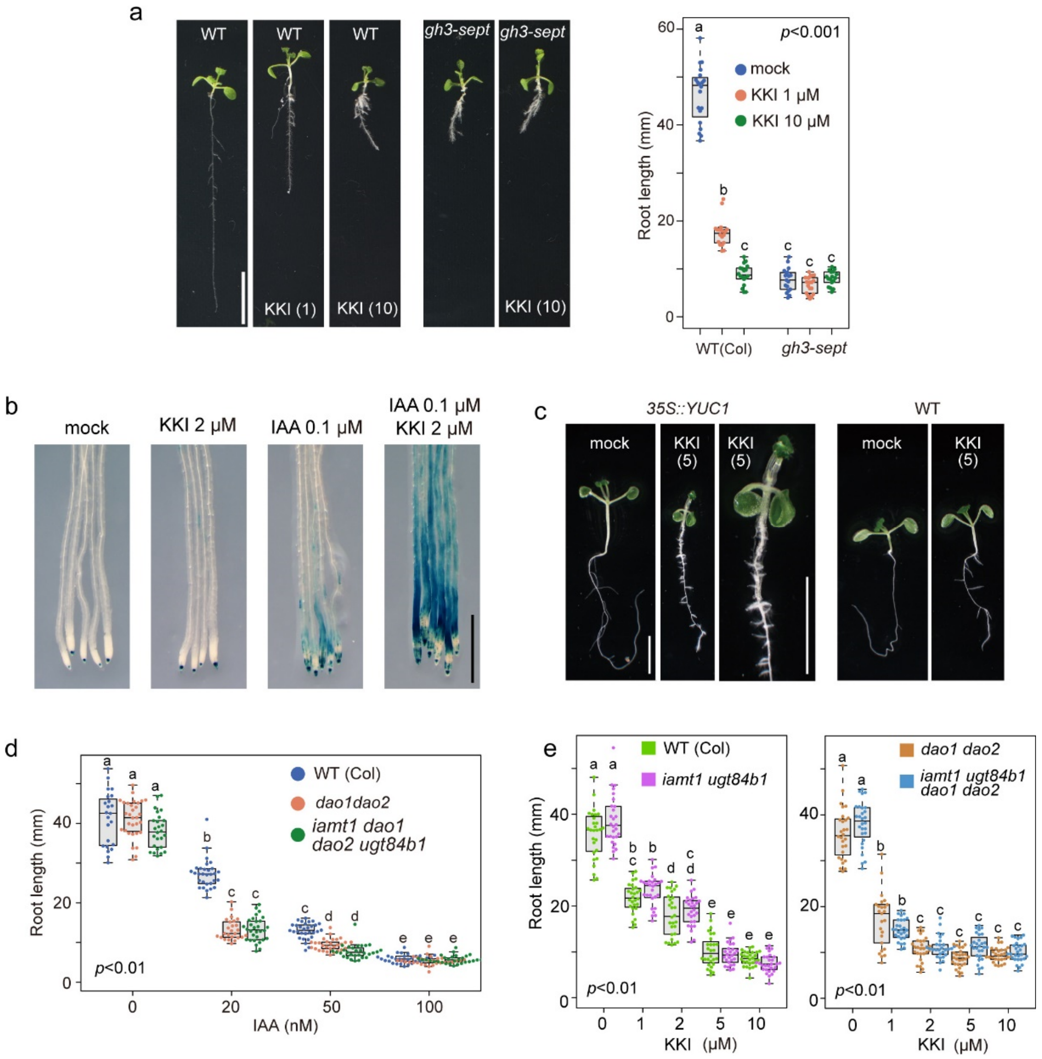

Supplementary Fig. 2. GH3 enzymes are involved in major IAA inactivation pathways.
(a) Effects of kakeimide (KKI, a GH3 inhibitor) on the *gh3-1 2 3 4 5 6 17* (*gh3-sept*) mutant (heterozygote in *gh3-9*). Scale bar, 10 mm. The *gh3-sept* roots were insensitive to KKI. The different letters represent statistical significance at *P*<0.001 (Tukey's HSD test, n = 17–20). (b) Effects of KKI on auxin-inducible *DR5::GUS* transgene expression. Six-day-old *DR5::GUS* seedlings were cultured for 6 h in liquid GM media with chemicals (μM). The auxin-induced GUS activity was visualized by X-Gluc. Scale bar, 1 mm. Cotreatment with IAA and KKI synergistically enhanced *DR5::GUS* expression. (c) Effects of KKI on the IAA-overproduction line *35S::YUC1*. The *35S::YUC1* is hypersensitive to KKI and displays extreme high-auxin phenotypes. Plants were grown on GM plates with KKI for 7 days. Scale bar, 5 mm. (d) The sensitivities of mutants in IAA inactivation enzymes to IAA. The primary root length was measured after 8 days of cultivation on vertical GM medium (6 g/L gellan gum) containing IAA. The mutation in *dao1 dao2* enhanced the sensitivity to IAA (20–50 nM) in the root. The different letters represent statistical significance at *P*<0.001 (Tukey's HSD test, n = 23–30). (e) The *iamt1 ugt84b1* double mutation did not affect the sensitivity to KKI in the presence or absence of *dao1* and *dao2*. The primary root length was measured after 8 days of cultivation on vertical GM medium (6 g/L gellan gum) containing KKI. These results suggest that IAMT1 and UGT84B1 do not function as alternative pathways to GH3. The different letters represent statistical significance at *P*<0.001 (Tukey's HSD test, n=25–28). (a,d, e) Box plots in all figures show the median as lines in the box, the 1st to 3rd quartiles as bounds of box, whiskers with end caps extending 1.5-fold interquartile range beyond the box, and the end caps represent the minimum and maximum.

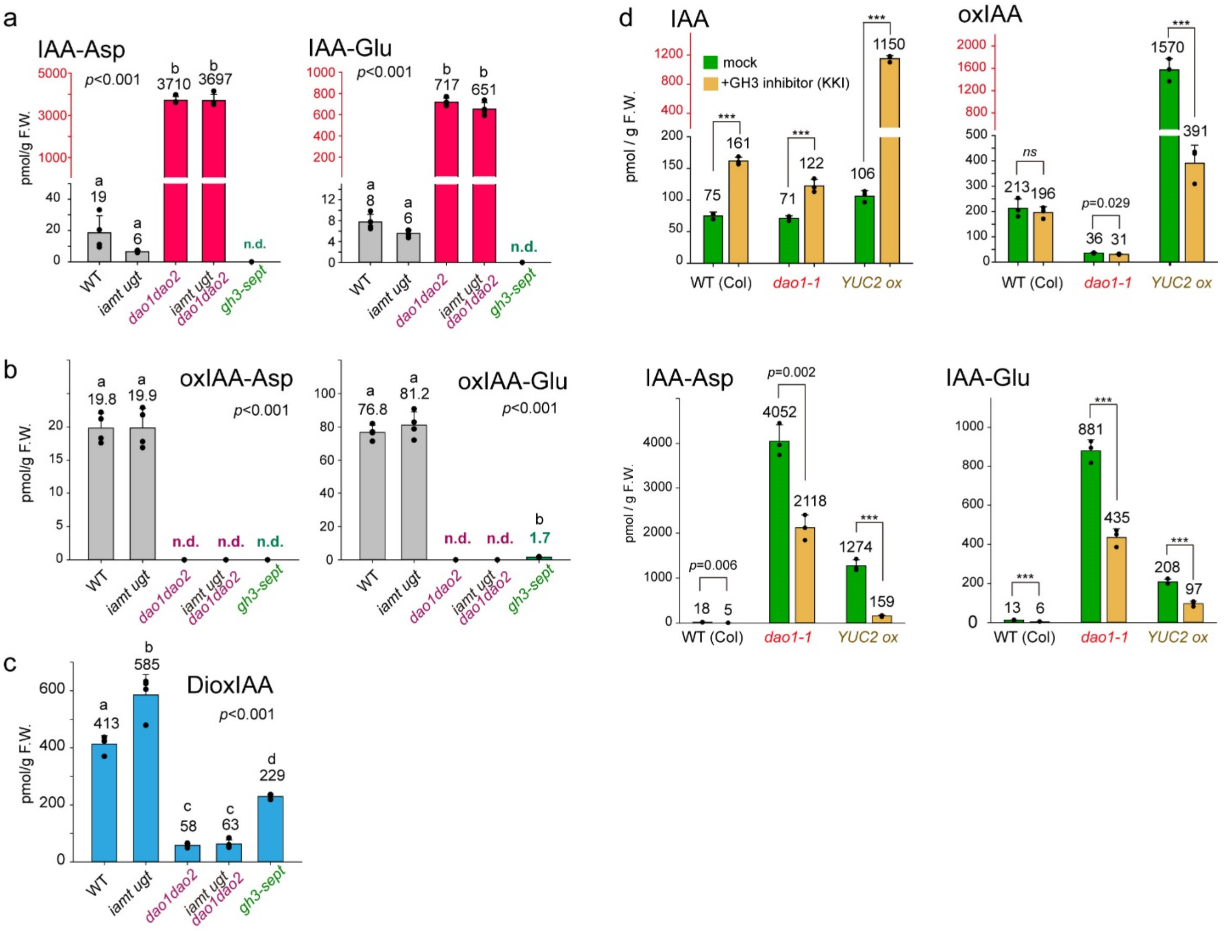

Supplementary Fig. 3. Endogenous amounts of IAA and its metabolites in IAA-inactivating enzyme mutants, *YUC2* overexpressing line, and WT plants treated with a GH3 inhibitor KKI.

(a–c) The levels of endogenous IAA-amino acid conjugates (a) and oxIAA-amino acid conjugates (b), and dioxIAA (c) in IAA-inactivating enzyme mutants (8-d-old) shown in Fig. 2c. (d) Eight-day-old WT, *dao1-1,* and *pMDC7::YUC2* seedlings were incubated in liquid GM media with or without 20 µM KKI in the presence of 2 µM estradiol (ER) for 36 h. After extensive washing of seedlings with fresh water, the metabolites were analyzed with LC-MS/MS. (a–c) The values shown are the means ± SD (n = 4). The different letters represent statistical significance at $P<0.01$ (Tukey's HSD test). n.d, less than the detection limit. (d) The values shown are the means ± SD (***$P < 0.001$, two-tailed Student's *t*-test, n = 3).

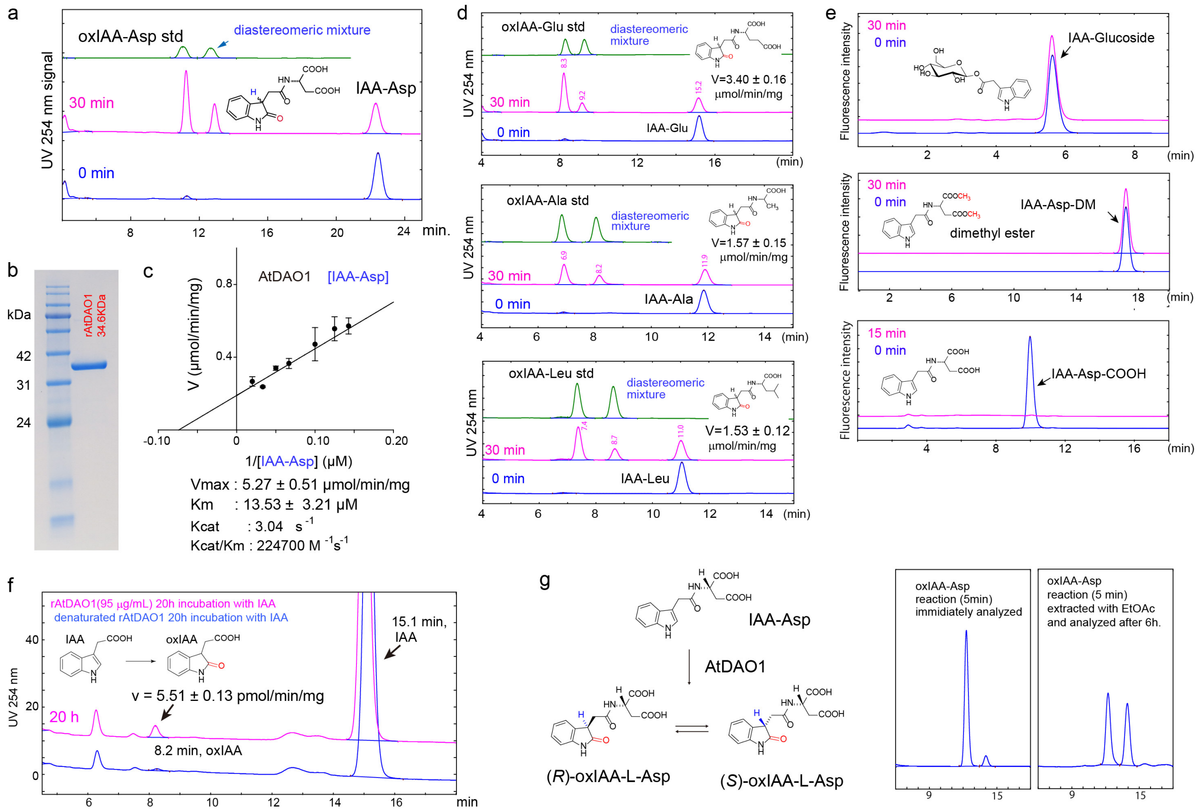

Supplementary Fig. 4. AtDAO1 catalyzes the oxidation of IAA-amino acid conjugates to yield corresponding oxIAA-amino acid conjugates.

(a) HPLC chromatogram of the reaction products of the AtDAO1 enzyme. IAA-Asp was used as a substrate. oxIAA-Asp was detected as a reaction product. (b) SDS-PAGE analysis of purified AtDAO1 protein (34.6 kDa) expressed in *E. coli* harboring the pCold-AtDAO1 vector. This experiment was repeated three times independently with similar results. (c) Lineweaver–Burk plot and kinetics parameters of AtDAO1 for IAA-Asp. (n=3). Error bars, SE. (d) HPLC chromatogram of the reaction products of the AtDAO1 enzyme. IAA-Glu, IAA-Ala, and IAA-Leu were used as substrates. oxIAA-Glu, oxIAA-Ala, and oxIAA-Leu were detected as reaction products from IAA-Glu, IAA-Ala, and IAA-Leu, respectively. (n=3) (e) HPLC chromatogram of the reaction mixtures of AtDAO1. IAA-β-D-glucoside, IAA-Asp dimethyl ester (IAA-Asp-DM), and IAA-Asp were used as substrates. IAA-Asp was completely consumed after 15 min of reaction. A total of 98% IAA-β-D-glucoside and 97% IAA-Asp-DM remained after the AtDAO1 reaction for 30 min. (f) AtDAO1 oxidizes IAA to produce oxIAA as a reaction product. The oxIAA production rate by AtDAO1 was 5.51 ± 0.13 pmol/min/mg under our assay conditions. (n=4) (g) oxIAA-amino acids were detected as a diastereomeric mixture from the reaction mixture. One of the diastereomers of oxIAA-Asp was readily epimerized during sample preparation.

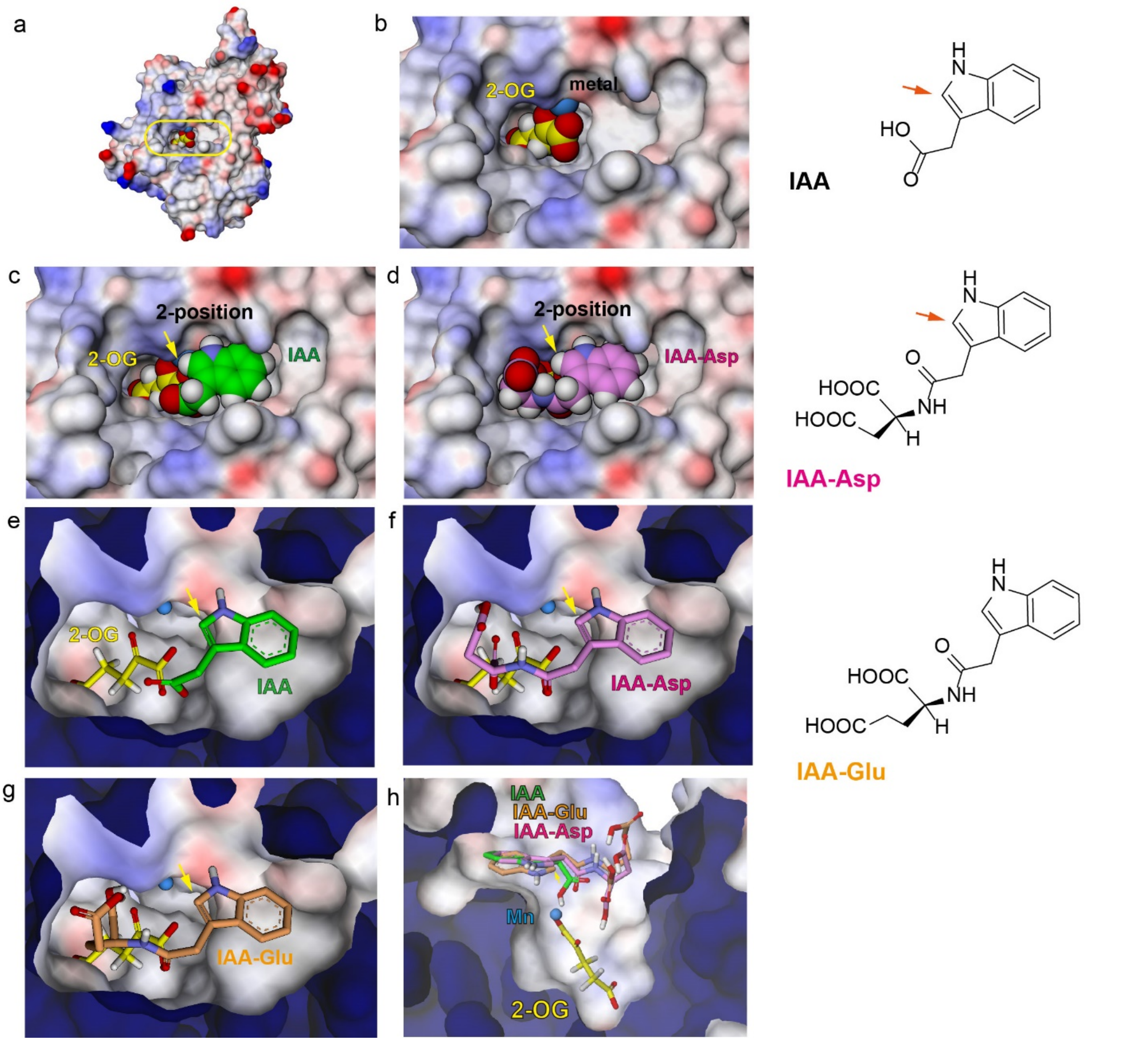

Supplementary Fig. 5. Docking study of IAA, IAA-Asp, and IAA-Glu with AtDAO1.
(a) AtDAO1 structure from the crystal coordinates (PDB ID: 6KWB). (b) The substrate-binding site of AtDAO1. AtDAO1 was co-crystalized with 2-oxoglutarate (2-OG: yellow color) and magnesium ion. (c-h) The predicted binding poses of IAA and IAA-amino acid conjugates in AtDAO1 were calculated by AutoDock Vina molecular docking software. The top scored poses are visualized as green molecules, IAA; magenta, IAA-Asp; cyan, IAA-Glu. The molecules are shown as space-filling models (c and d) or stick models (e–h). The IAA moiety was located at the same position among the IAA and IAA-amino acid conjugates. The 2-position of the IAA moiety was oriented to 2-OG and the magnesium ion (arrows). IAA could not fill the binding site. On the other hand, the amino acid moieties of IAA-Asp and IAA-Glu occupied the entire binding pocket of AtDAO1. Under this calculation condition, the affinity scores (Kd) of IAA, IAA-Asp, IAA-Glu, IAA-Leu, and IAA-Ala were estimated to be –6.1, –7.7, –7.1, –7.0, and –7.5, respectively.

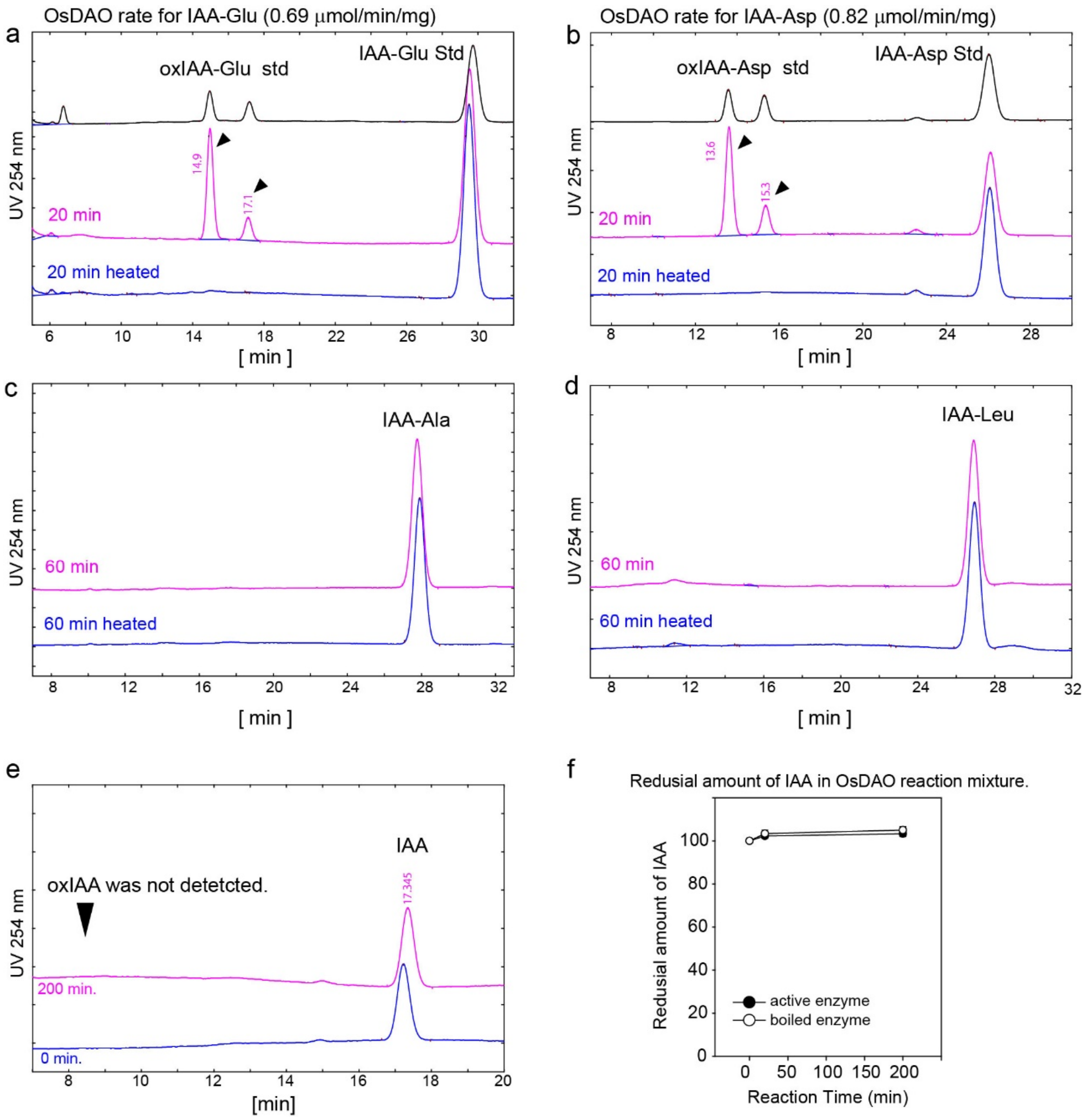

Supplementary Fig. 6. OsDAO functions as an IAA-amino acid conjugate oxidase to produce oxIAA-amino acids.

Recombinant OsDAO was expressed in *E. coli* BL21 harboring pCold-OsDAO vector and chaperonin vector pG-Tf2. (a and b) HPLC chromatogram of the reaction products of the OsDAO1 enzyme. IAA-Glu and IAA-Asp were used as substrates. OsDAO oxidized IAA-Glu and IAA-Asp to produce oxIAA-Glu and oxIAA-Asp, respectively. (c and d) HPLC chromatogram of the reaction mixture of the OsDAO enzyme. IAA-Ala and IAA-Leu were used as substrates. oxIAA-Ala and oxIAA-Leu were not detected, and more than 97% of IAA-Ala and IAA-Leu remained after 60 min of reaction. (e and f) oxIAA was not detected in the reaction mixture of OsDAO after 200 min incubation with IAA as the substrate (e), and IAA was not reduced after the reaction (n=4, f). The reaction was conducted under the same conditions as IAA-Glu (20 min reaction time).

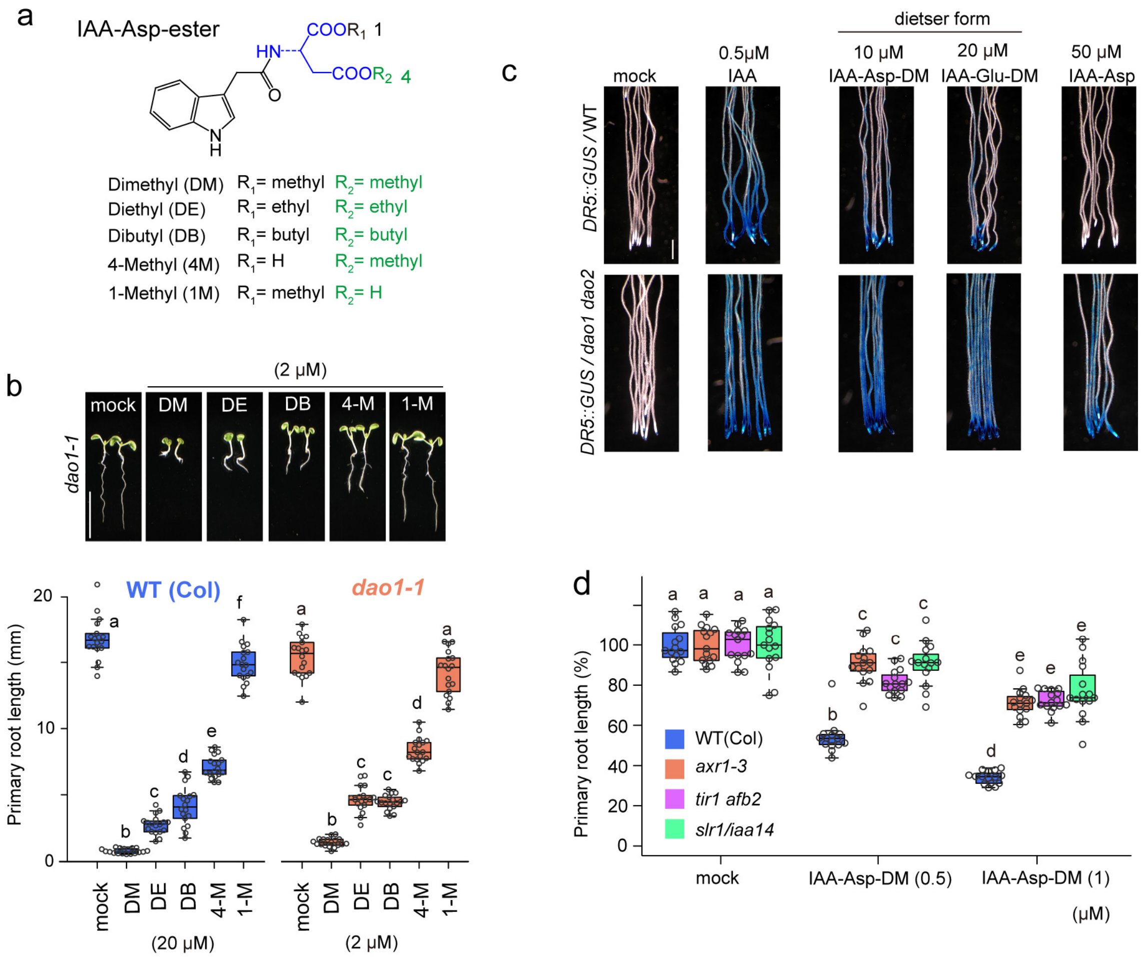

Supplementary Fig. 7. IAA-Asp functions as a storage form of IAA.

(a) Chemical structures of membrane-permeable IAA-Asp diesters [dimethyl ester (DM), diethyl ester (DE), dibutyl ester (DB), and IAA-Asp monomethyl esters [4-methyl ester (4-M) and 1-methyl ester (1-M)]. (b) IAA-Asp diesters inhibited primary root growth as typical auxin activity in both *dao1-1* mutant and WT (Col) plants. Monomethyl esters of IAA-Asp (4-M and 1-M) were less active in the root auxin response than diesters of IAA-Asp. WT and *dao1-1* mutant seedlings were grown for 6 days on GM plates containing 20 µM compounds (for wild-type plants) or 2 µM compounds (for *dao1-1* mutants). The different letters represent statistical significance at $P<0.01$ (Tukey's HSD test, n = 18). Scale bar, 10 mm. (c) Auxin-inducible *DR5::GUS* transgene expression in *dao1dao2* and WT seedlings. Six-day-old seedlings were cultured for 8 h in liquid GM media with chemicals (µM). The auxin-induced GUS activity was visualized by X-Gluc. Scale bar, 1 mm. (d) The auxin-resistant mutants *axr1-3*, *tir1 afb2*, and *slr1/iaa14* were resistant to IAA-Asp-DM. The WT and mutant plants were grown on GM media (4 g/L agar) containing IAA containing IAA-Asp-DM for 6 days. Relative root length is shown as the percentage of that in mock-treated plants (100%). The different letters represent statistical significance at $P<0.005$ (Tukey's HSD test, n = 15).

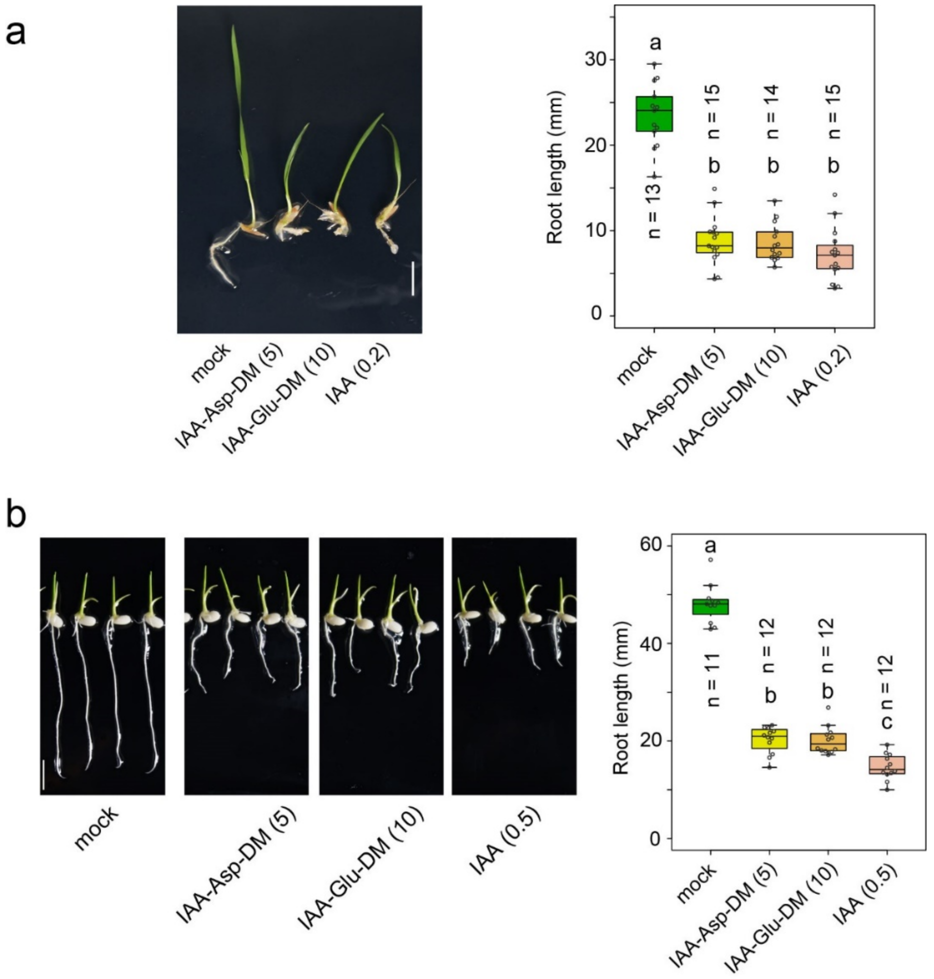

Supplementary Fig. 8. IAA-amino acid diesters showed auxin activity in *Brachypodium distachyon* and rice (*Oryza sativa*).

(a) *Brachypodium distachyon* Bd21 seeds were cultured for 8 days on GM agar medium with hormones. Scale bar, 10 mm. (b) *Oryza sativa* was cultured for 5 days in distilled water containing hormones. The different letters represent statistical significance at P<0.001 (Tukey's HSD test), and n indicates the number of plants analyzed. Scale bar, 10 mm. Similar to IAA, both IAA-Asp-DM and IAA-Glu-DM inhibited root growth in *B. distachyon* and rice seedlings.

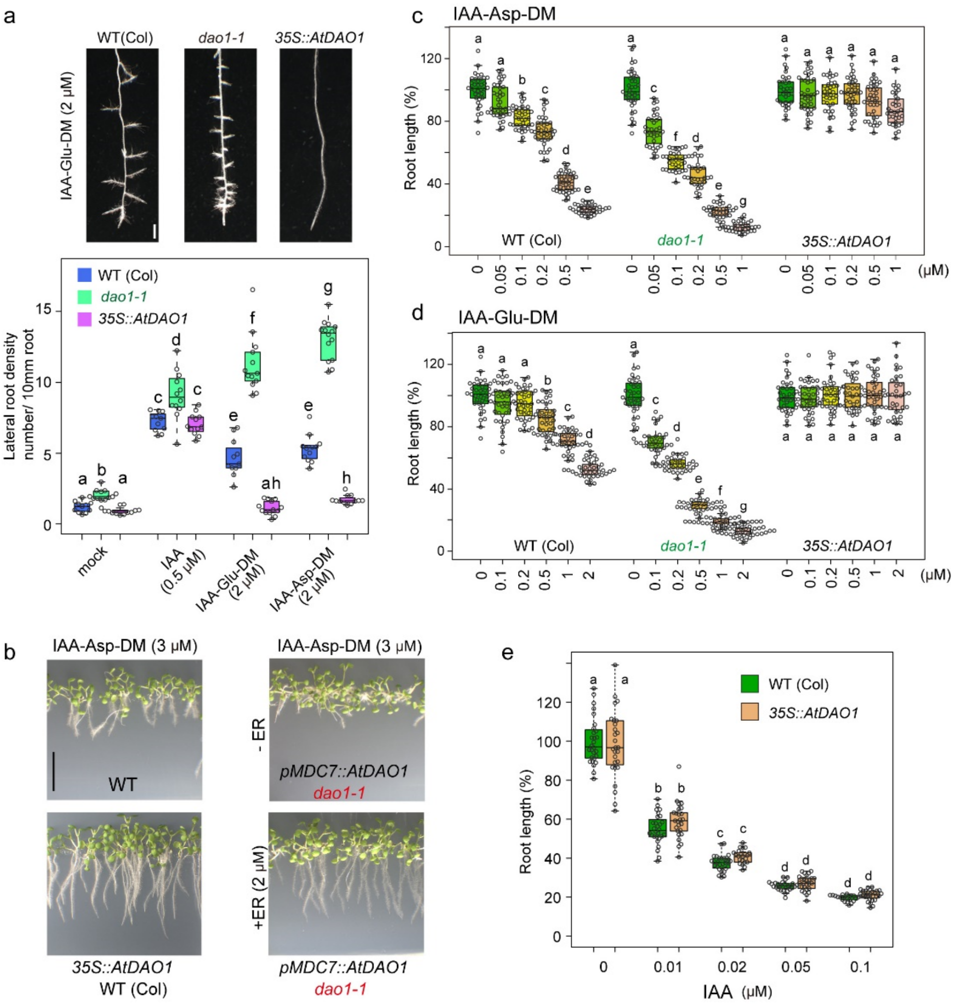

Supplementary Fig. 9. AtDAO1 modulates auxin activity derived from IAA-Asp and IAA-Glu.
(a) Lateral root formation in *dao1-1* and *AtDAO1*-overexpressing plants (*35S::AtDAO1*). Five-day-old plants were incubated for an additional 3 days on a horizontal GM plate containing IAA and conjugates. IAA induced lateral root formation of WT (Col), *dao1-1*, and *35S::AtDAO1* plants to the same extent. IAA-Asp-DM and IAA-Glu-DM induced lateral root formation in WT (Col) and *dao1-1* but not in *35S::AtDAO1* plants. The *dao1-1* mutant was more sensitive to the conjugates than WT plants. The different letters represent statistical significance at $P<0.001$ (Tukey's HSD test, n = 11–14). Scale bar, 1 mm. (b) Phenotype of the *AtDAO1* overexpression line. The seedlings were grown vertically for 7 days on GM plates containing 2 μM estradiol (ER) with or without IAA-Asp-DM (3 μM). ER was added to induce the *AtDAO1* transgene in *pMDC7::AtDAO1* in the *dao1-1* mutant. Scale bar, 10 mm. (c, d) The primary root length of WT and *35S::AtDAO1* and *dao1-1* mutants cultured for 6 days on GM plates (4 g/L agar) containing IAA-Asp-DM (c) and IAA-Glu-DM (d). Relative root length is shown as the percentage of that in mock-treated plants (100%). The different letters represent statistical significance at $P<0.001$ (Tukey's HSD test, n = 34–40). (e) *35S::AtDAO1* and WT roots showed the same sensitivity to IAA. Seedlings were cultured on a vertical GM plate (6 g/L gellan gum) containing IAA for 6 days. The different letters represent statistical significance at $P<0.001$ (Tukey's HSD test, n = 22–28). AtDAO1 predominantly inactivates IAA-amino acid conjugates rather than IAA in planta.

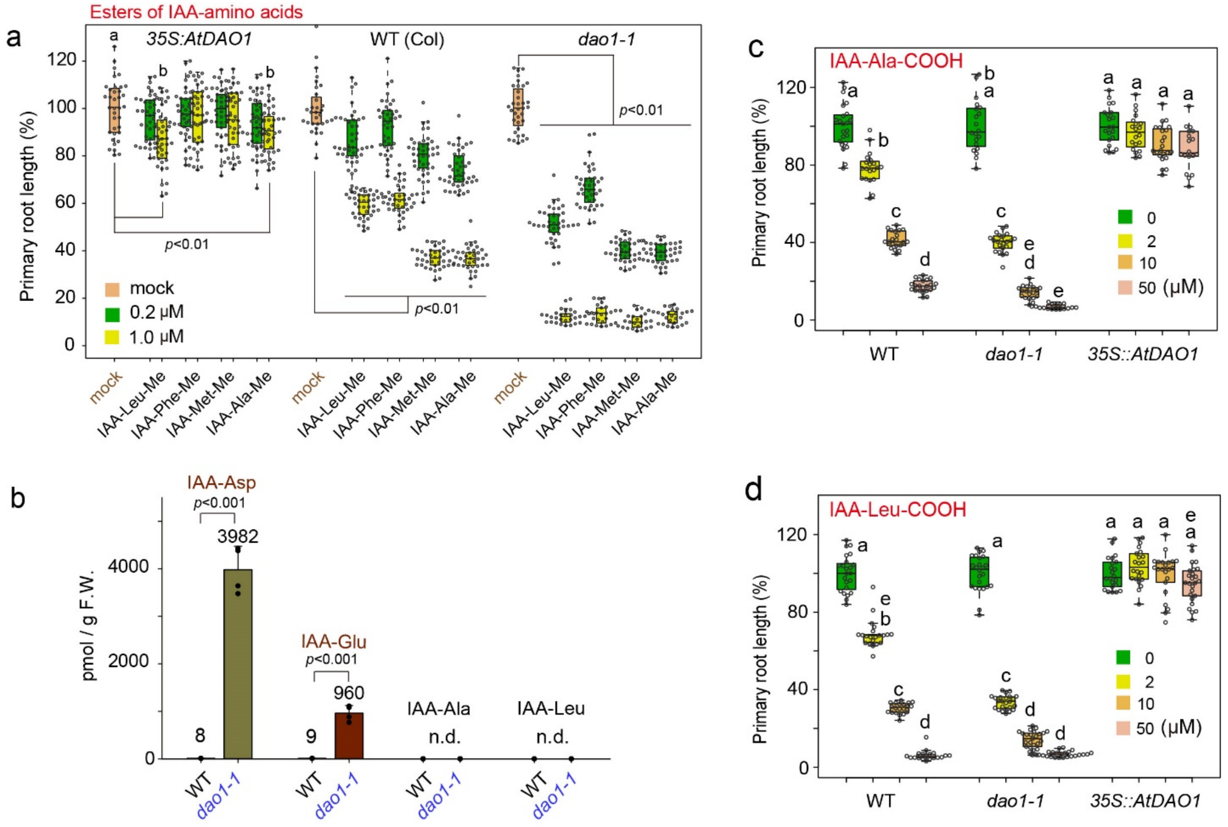

Supplementary Fig. 10. Endogenous levels of IAA-amino acid conjugates in wild-type and *dao1-1*, and the effects of IAA-amino acid conjugates on *dao1* mutants.

(a) Effects of IAA-amino acid esters on root growth in the *dao1-1 and 35S::AtDAO1* lines. P values were determined by Tukey's HSD relative to mock treatment (n= 23–44). (b) Endogenous levels of IAA-Asp, IAA-Glu, IAA-Ala, and IAA-Leu in WT (Col) and *dao1-1* mutant plants. IAA-amino acid conjugates in 7-d-old seedlings were measured by LC-MS/MS. IAA-Asp and IAA-Glu accumulated substantially in the *dao1-1* mutant. IAA-Ala and IAA-Leu levels were below our detection limit in both the WT and *doa1-1* mutant plants. The different letters represent statistical significance at P<0.001 (Tukey's HSD test, n = 4). (c and d) Effects of IAA-Ala and IAA-Leu on root growth in the *dao1-1 and 35S::AtDAO1* lines. (a, c, and d) Seedlings were grown for 6 days on GM plates (4 g/L agar) with IAA-Ala and IAA-Leu. After cultivation, the primary root lengths of the WT (Col), *35S::AtDAO1,* and *dao1-1* mutant plants were measured. Relative root length is shown as the percentage of that in mock-treated plants (100%). The *35S::AtDAO1* overexpression line was resistant to various IAA-amino acid conjugates, but the *dao1-1* mutant was hypersensitive to these conjugates. The different letters represent statistical significance at P<0.001 [Tukey's HSD test, (c) n = 19–26, (d) n = 17–23].

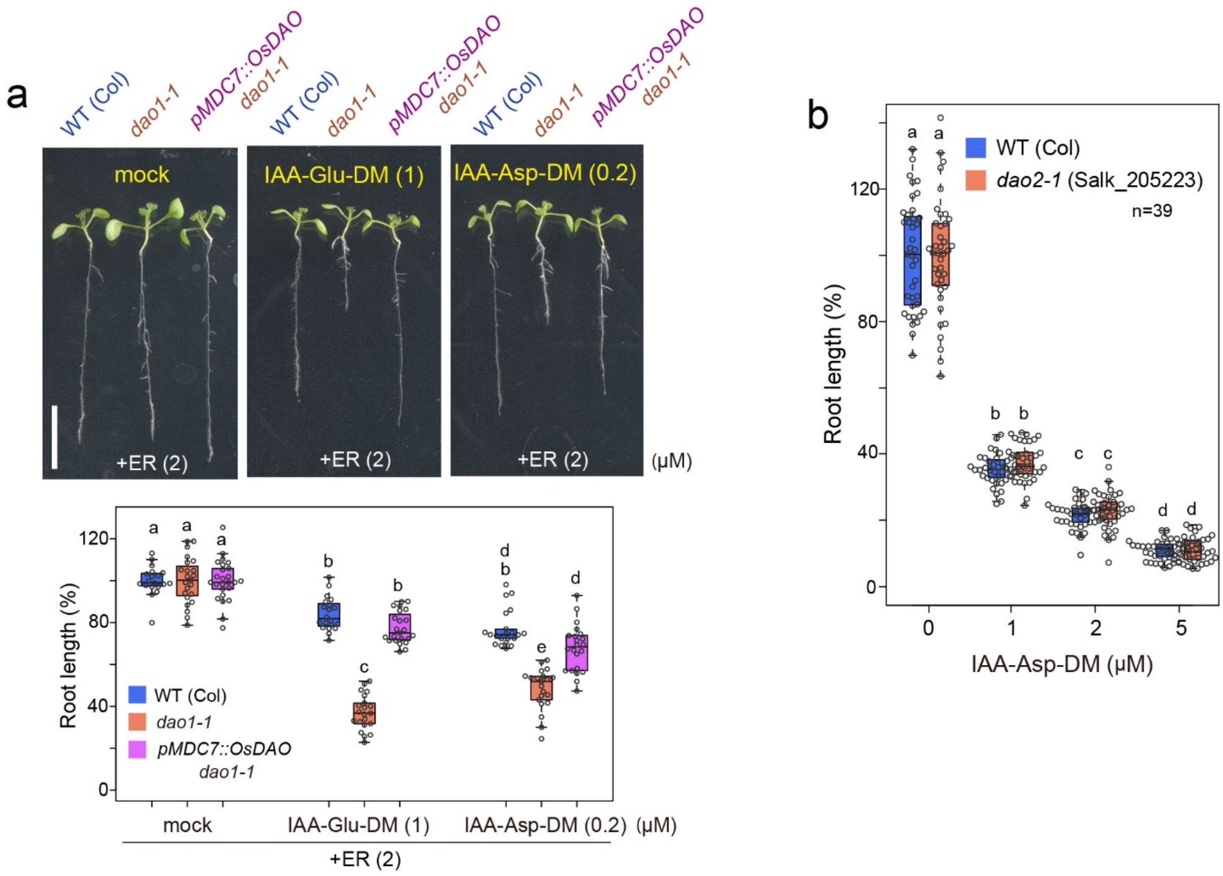

Supplementary Fig. 11. Rice OsDAO complements the hyper-sensitivity of *dao1-1* to IAA-amino acid diesters, and the *Atdao2-1* mutant showed the same sensitivity as WT to IAA-Asp-DM.

(a) The overexpression of *OsDAO* decreased the sensitivity of the *dao1-1* mutant to IAA-Glu-DM and IAA-Asp-DM. *pMDC7::OsDAO* lines were grown for 7 days on GM vertical plates containing 2 μM ER with or without IAA-Glu-DM. Relative root length is shown as the percentage of that in mock-treated plants (100%). Scale bar, 10 mm. The different letters represent statistical significance at $P<0.01$ (Tukey's HSD test, n= 19–25). (b) *Arabidopsis dao2-1* loss-of-function mutants showed the same response to IAA-Asp-DM as wild-type plants. Plants were cultured for 7 days on GM plates with IAA-Asp-DM. The different letters represent statistical significance at $P<0.001$ (Tukey's HSD test, n = 39).

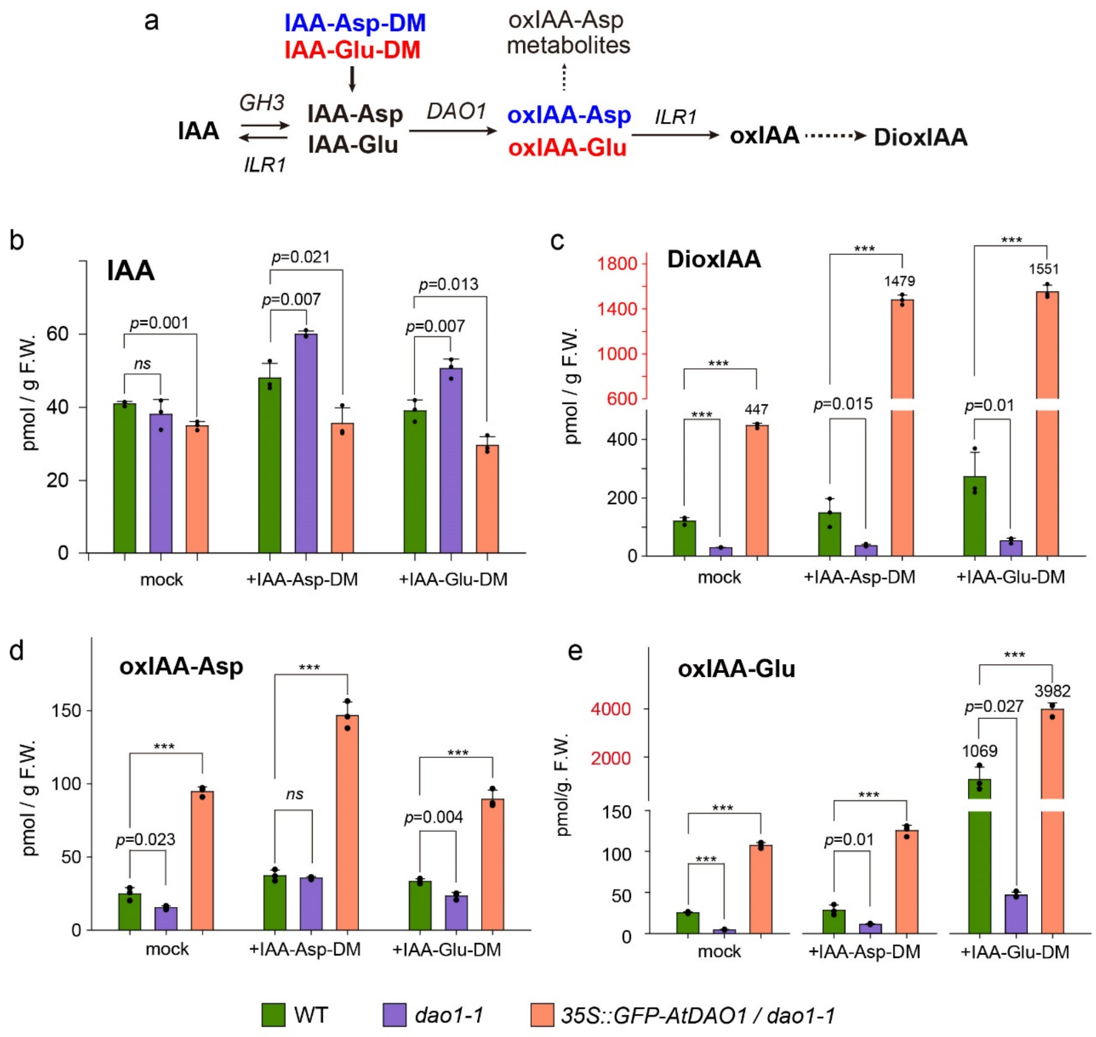

Supplementary Fig. 12. Endogenous amounts of IAA and its metabolites in *dao1-1* and *GFP-AtDAO1*-overexpressing plants.

(a) Our working model of the IAA inactivation pathway is shown. (b–e) Seven-day-old WT, *dao1-1,* and *35S::GFP-AtDAO1* in *dao1-1* seedlings were incubated in liquid GM media with or without 0.5 μM IAA-Asp-DM and IAA-Glu-DM for 20 h. After extensive washing of seedlings with fresh water, the metabolites were analyzed with LC-MS/MS. The levels of endogenous IAA (b), DioxIAA (c), oxIAA-Asp (d), and oxIAA-Glu (e) in WT, *dao1-1,* and the *GFP-AtDAO1*-overexpressing *dao1-1* line are shown. The values shown are the means ± SD (****P* < 0.001, *ns*, not significant, two-tailed Student's *t*-test, n = 3).

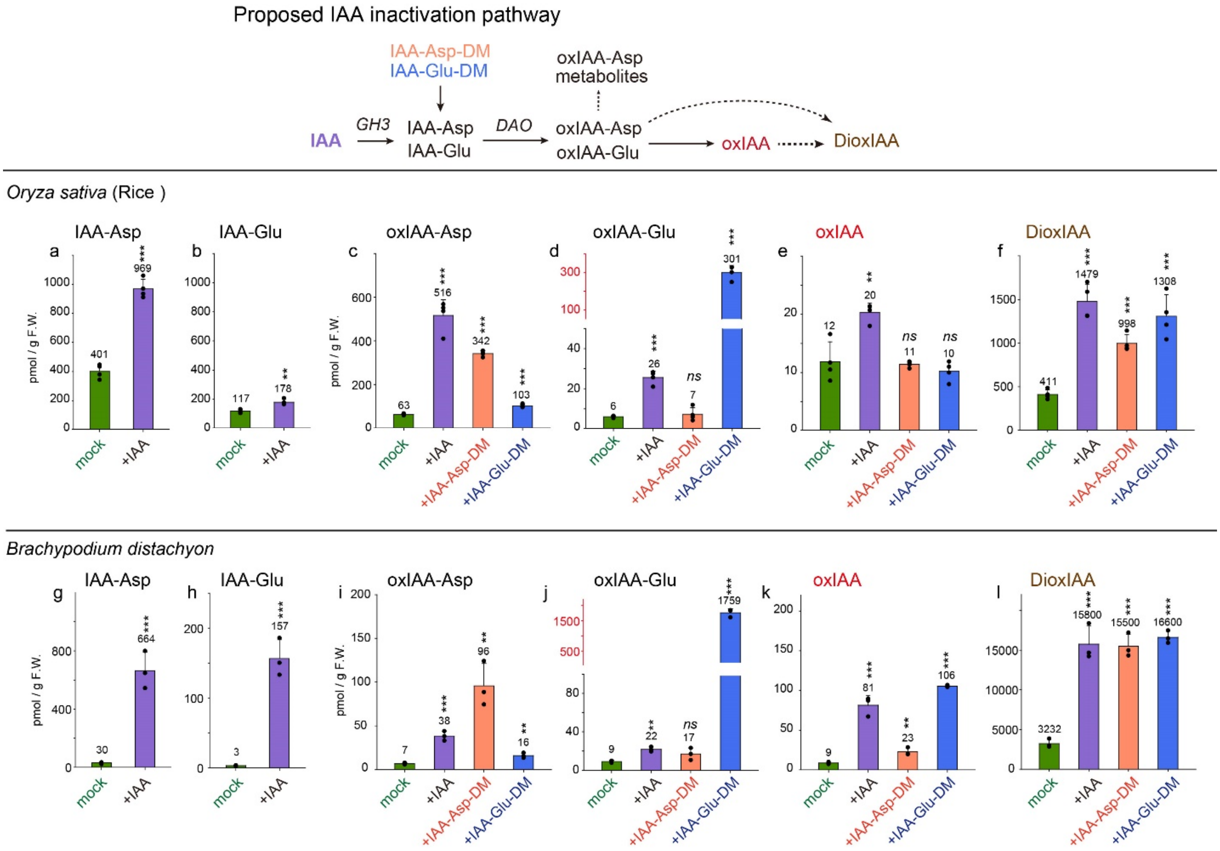

Supplementary Fig. 13. Endogenous amounts of IAA metabolites in rice (*Oryza sativa*) and *Brachypodium distachyon*.

Our working model of the IAA inactivation pathway is shown. (a–f) *Oryza sativa* cv. Nipponbare was grown on agar (3 g/L) for 5 days at 25 °C under continuous light after 2 days of incubation at 28 °C. The plants were incubated in water with 1 µM compounds for 24 h. After extensive washing of seedlings with fresh water, the root was excised. The metabolites in the root were analyzed with LC-MS/MS. The values shown are the means ± SD (***$P < 0.001$, **$P < 0.01$, *ns*: not significant, Two-tailed Student's *t*-test [vs mock treatment], n = 4). (g–l) *Brachypodium distachyon* Bd21 was grown hydroponically with 1/10 GM medium (without sucrose) for 15 days at 24 °C under continuous light. The plants were incubated in water with 1 µM compounds for 24 h. After extensive washing of seedlings with fresh water, the root was excised. The metabolites in the root were analyzed with LC-MS/MS. The values shown are the means ± SD (***$P < 0.001$, **$P < 0.01$, *ns*: not significant, Two-tailed Student's *t*-test [vs mock treatment], n = 3).

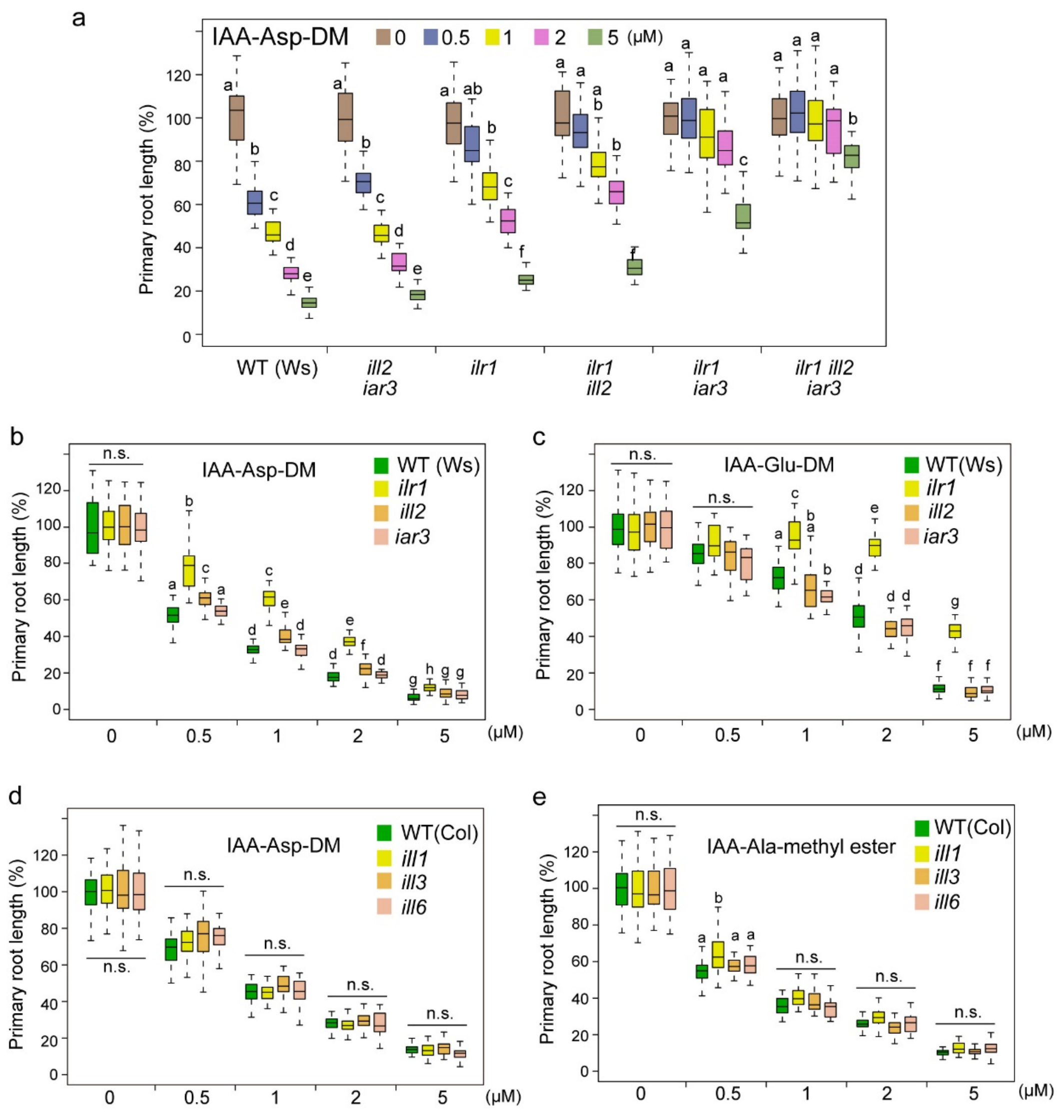

Supplementary Fig. 14. ILR1/ILL enzymes are responsible for the conversion of IAA-Asp and IAA-Glu to IAA.

(a–e) Effects of IAA-Asp-DM and IAA-Glu-DM on root growth of the single, double, and triple mutants of *ilr1*, *ill2*, and *iar3*. The *ilr1* and *iar3* (*ilr1-1* and *ilr3-2*) mutants are EMS lines, and the *ill2* mutant (*ill2-1*) is a T-DNA insertion line in Ws background. (d and e) Effects of IAA-Asp-DM and IAA-Glu-DM on root growth of *ill1*, *ill3*, and *ill6* single mutants The *ill1*, *ill3*, and *ill6* mutants are T-DNA insertion lines in Col background. (a–e) The seedlings were grown for 7 days on GM plates with or without the indicated conjugates. Relative root length is shown as the percentage of that in mock-treated plants (100%). The different letters represent statistical significance at $P<0.001$ (ns: not significant, Tukey's HSD test, (a) n= 32–42 (average=36), (b) n= 33, (c) n= 35, (d) n= 37, (e) n= 30).

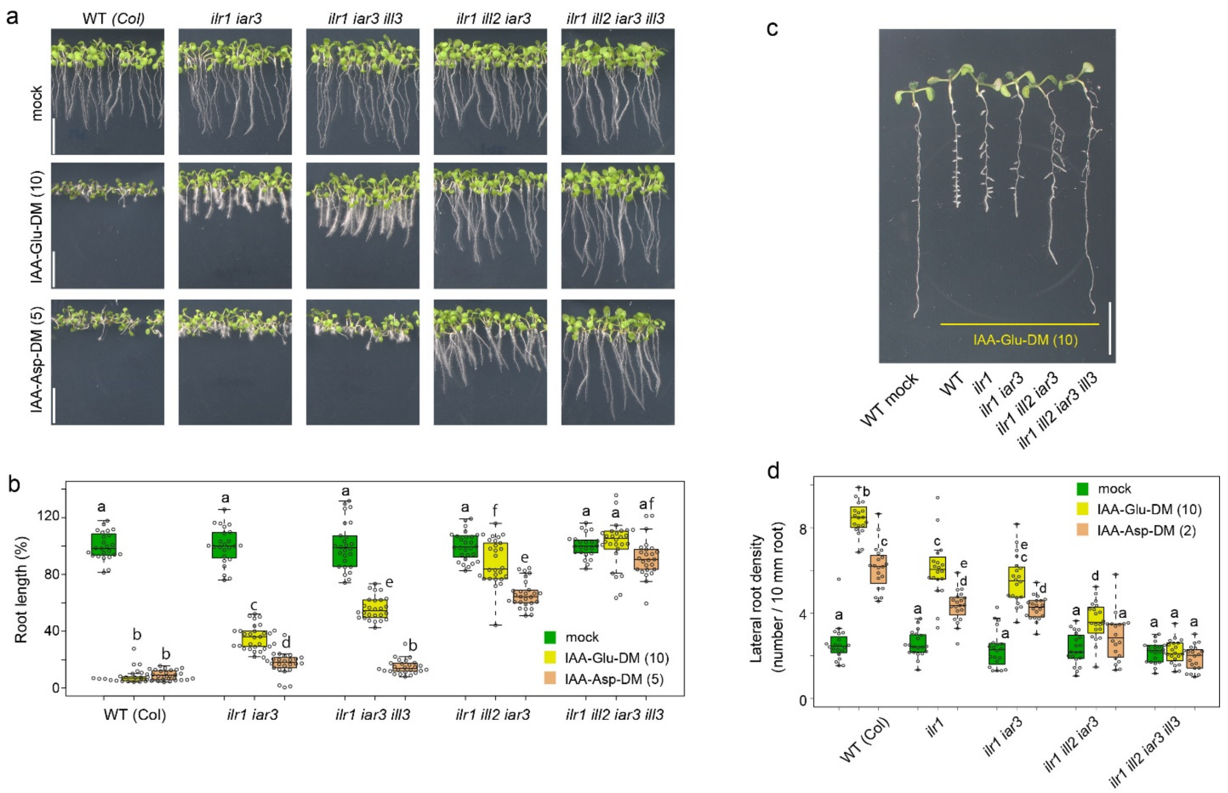

Supplementary Fig. 15. *ILR1*, *ILL2*, *IAR3,* and *ILL3* are required for the conversion of IAA-Asp and IAA-Glu to IAA.

(a and b) The sensitivity of root growth of *ilr1*, *ill2*, *iar3,* and *ill3* multiple mutants to IAA-Asp-DM and IAA-Glu-DM. The *ilr1/ill* mutants are T-DNA insertion lines (Col background). The seedlings were vertically grown for 6 days on GM plates (6 g/L gellan gum) with or without the conjugates. Relative root length is shown as the percentage of that in mock-treated plants (100%). Scale bars, 10 mm. (c and d) Lateral root formation of *ilr1*, *ill2*, *iar3,* and *ill3* multiple mutants (Col background) treated with IAA-Asp-DM and IAA-Glu-DM. Five-day-old plants were incubated for an additional 3 days on a horizontal GM plate containing hormones. Scale bars, 10 mm. The different letters represent statistical significance at $P<0.001$ [Tukey's HSD test, (b) n= 24–30, (d) n= 20].

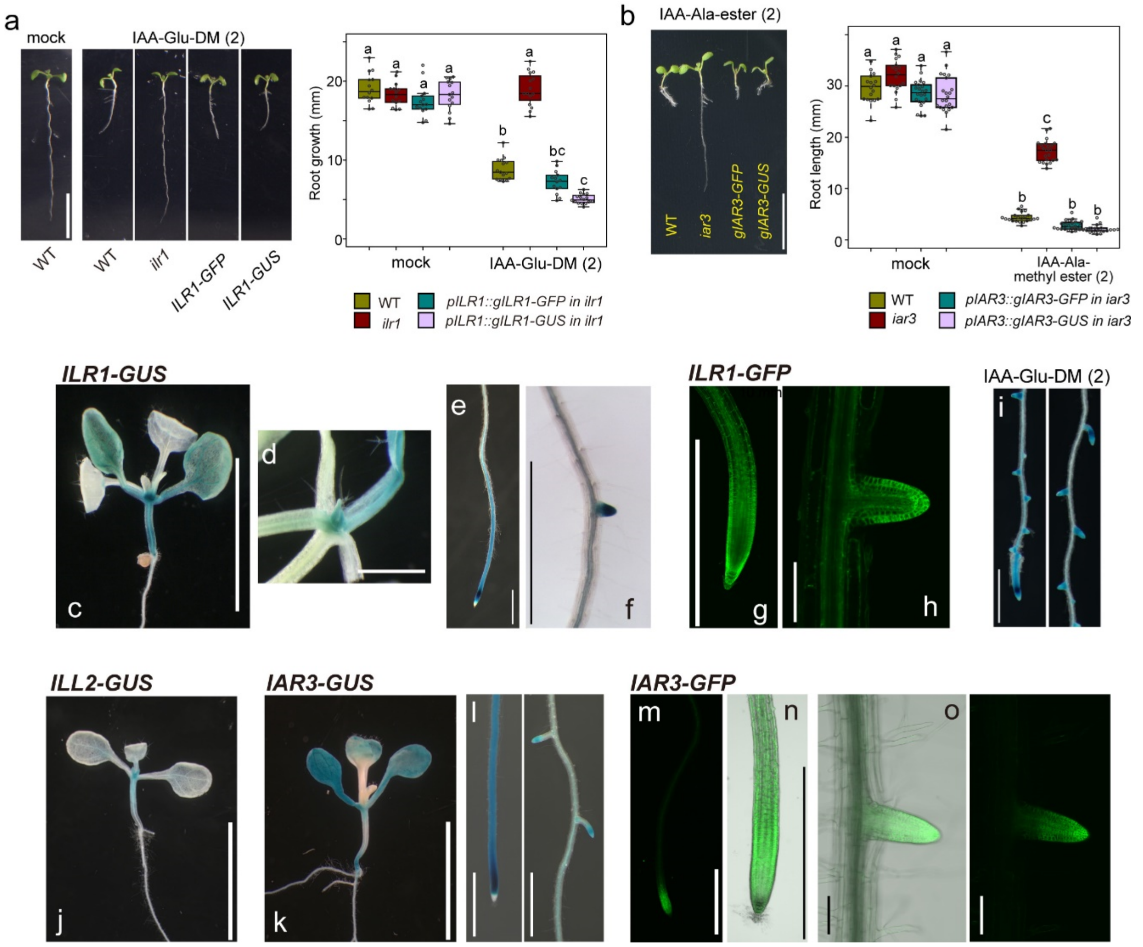

Supplementary Fig. 16. Expression patterns of *ILR1*, *ILL2* and *IAR3*.
(a) The *pILR1::ILR1-GFP* and *pILR1::ILR1-GUS* transgenes functionally complemented the IAA-Glu-DM-insensitive phenotype of the *ilr1* mutant (Col background). The *pILR1::ILR1-GFP* and *pILR1::ILR1-GUS* plants were grown on GM plates containing 2 μM IAA-Glu-DM for 7 days. Scale bar, 10 mm. (b) *pIAR3::IAR3-GFP* and *pIAR3::IAR3-GUS* transgenes functionally complemented the IAA-Ala ester-insensitive phenotype of the *iar3* mutant (Col background). The *pIAR3::IAAR3-GFP* and *pIAR3::IAR3-GUS* plants were grown on GM plates containing 2 μM IAA-Ala methyl ester for 6 days. Scale bar, 10 mm. The different letters represent statistical significance at $P<0.001$ [Tukey's HSD test, (a) n=14–16, (b) n=15–21]. (c–h). *pILR1::ILR1-GUS* and *pILR1::ILR1-GFP* seedlings were grown for 9 days on vertical GM plates. (i) Six-day-old *pILR1::ILR1-GUS* seedlings were incubated with 2 μM IAA-Glu-DM for another 3 days. The *pILR1::ILR1-GUS* plants were placed in X-Gluc staining buffer until sufficiently stained. (j) *ILL2-GUS* expression in 8-day-old *pILL2::ILL2-GUS* seedlings. (k–o), *pIAR3::IAR3-GUS* (k and l) and *pIAR3::IAR3-GFP* (m–o) plants were grown for 9 days on vertical GM plates. At least five seedlings from three independent lines were examined. This experiment was repeated three times independently with similar results. Scale bars, 10 mm (c, j, and k), 1 mm (d, e, f, g, i, l, m, n), and 100 μm (h and o).

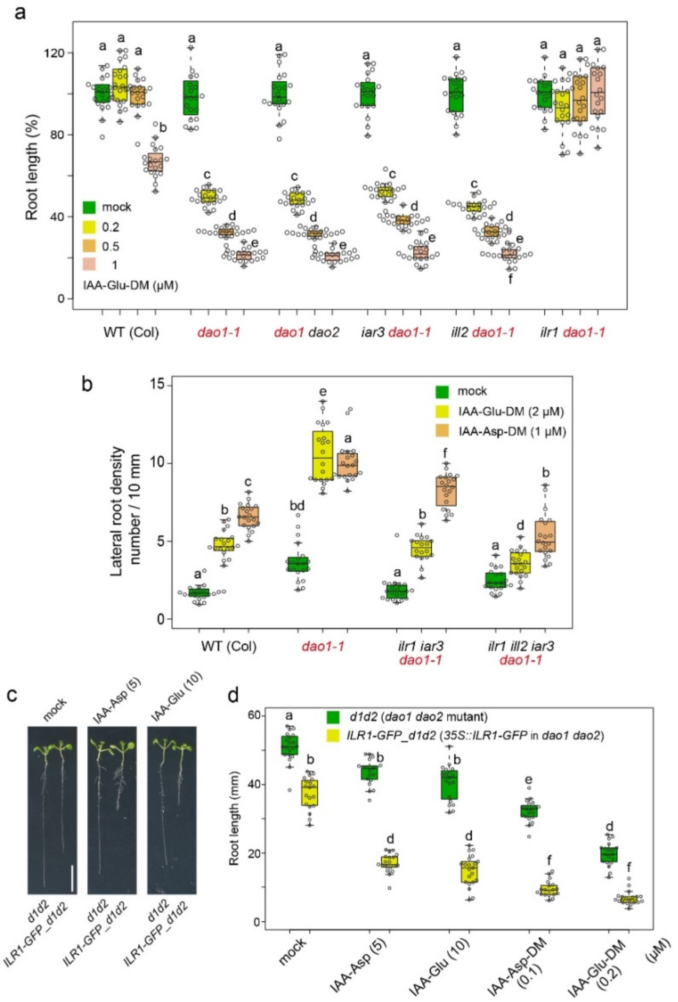

Supplementary Fig. 17. The responses of the *dao1* mutant to IAA-Asp and IAA-Glu esters depend on ILR1.

(a) Effects of IAA-Glu-DM on root growth of *dao1 ilr/ill* mutants (*dao1-1*, *dao1 dao2*, *iar3 dao1-1*, *ill2 dao1-1*, and *ilr1 dao1-1*). The seedlings were grown for 6 days on GM plates (4 g/L agar) containing IAA-Glu-DM. Relative root length is shown as the percentage of that in mock-treated plants (100%). The *ilr1* mutation decreased high sensitivity of root inhibition in the *dao1-1* mutant. The different letters represent statistical significance at $P<0.01$ (Tukey's HSD test, n = 20). (b) Effects of IAA-Asp-DM and IAA-Glu-DM on lateral root formation of *dao1 ilr/ill* mutants (*dao1-1*, *ilr1 iar3 dao1-1*, and *ilr1 ill2 iar3 dao1-1*). The seedlings were grown vertically for 6 days on GM plates and then transferred to a horizontal GM plate containing compounds. The seedlings were cultured for another 3 days. The root length and lateral root number were measured. The different letters represent statistical significance at $P<0.01$ (Tukey's HSD test, n =20). (c and d) Overexpression of the ILR1-GFP fusion protein enhanced the sensitivity of the *dao1 dao2* mutant to IAA-Asp and IAA-Glu and their diesters (IAA-Asp-DM and IAA-Glu-DM). The seedlings were grown vertically for 8 days on GM plates (6 g/L gellan gum) containing IAA conjugates. Primary root length was measured. Scale bar, 10 mm. The different letters represent statistical significance at $P<0.001$ (Tukey's HSD test, n = 19-20).

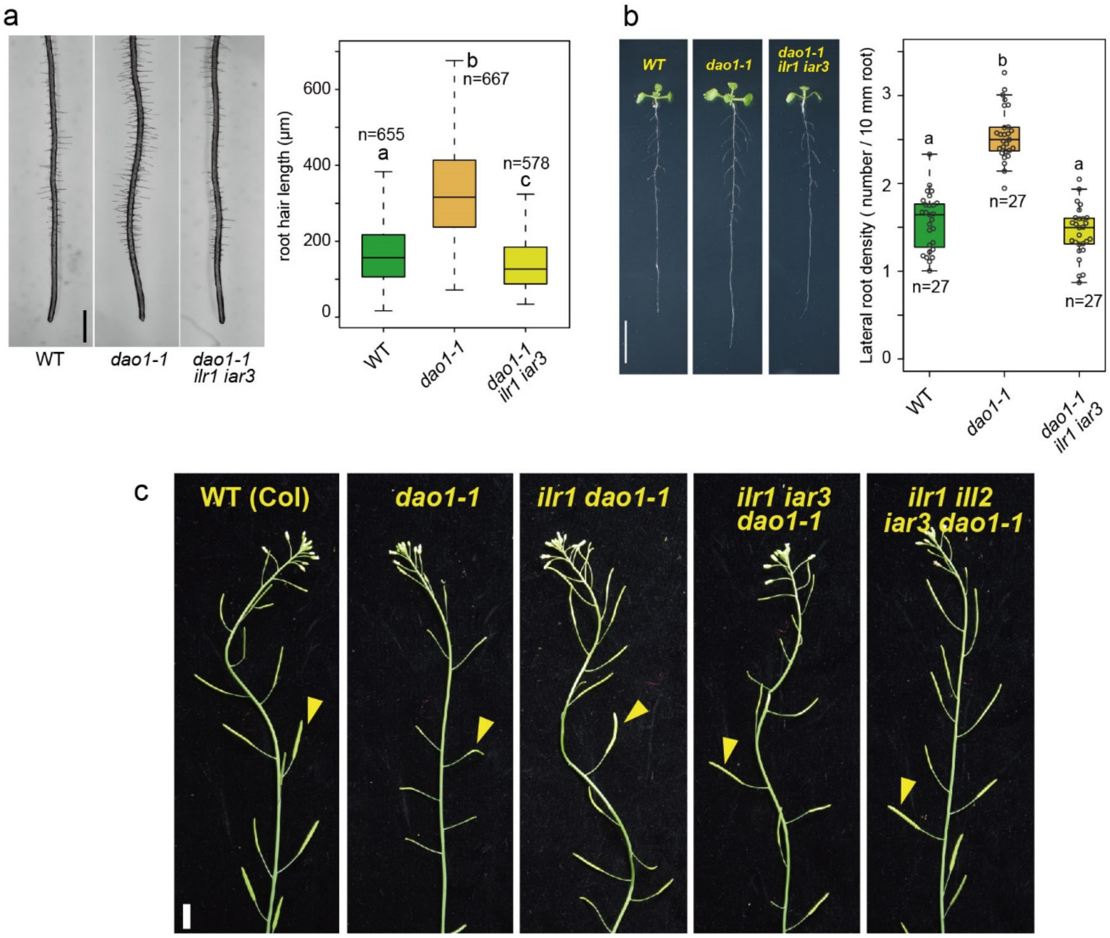

Supplementary Fig. 18. The *ilr1 iar3* loss-of-function mutation restored *dao1-1* phenotypes.
(a) Root hair length of wild-type (Col), *dao1-1,* and *ilr1 iar3 dao1-1* triple mutant plants. Scale bar, 1 mm. The seedlings were grown vertically for 9 days on GM plates (6 g/L gellan gum). Primary root hair length was measured. The different letters represent statistical significance at $P<0.001$ (Tukey's HSD test). The values shown are the means, and n indicates the number of root hairs analyzed. Scale bar, 1 mm. (b) Lateral root density in wild-type, *dao1-1,* and *ilr1 iar3 dao1-1* mutant plants. The seedlings were grown vertically for 9 days on GM plates. The number of lateral roots and root length were measured. Scale bar, 10 mm. The different letters represent statistical significance at $P<0.01$ (Tukey's HSD test). $P=0.348$ for letter a. (c) Phenotypes of primary inflorescences from 40-day-old WT (Col), *dao1-1*, *ilr1 dao1-1*, *ilr1 iar3 dao1-1* and *ilr1 ill2 iar3 dao1-1* mutant plants (Col background). The *ilr1* mutation restored less fertility in primary inflorescences in the *dao1-1* mutant. At least five plants from each line were examined. Scale bar, 10 mm. This experiment was repeated three times independently.

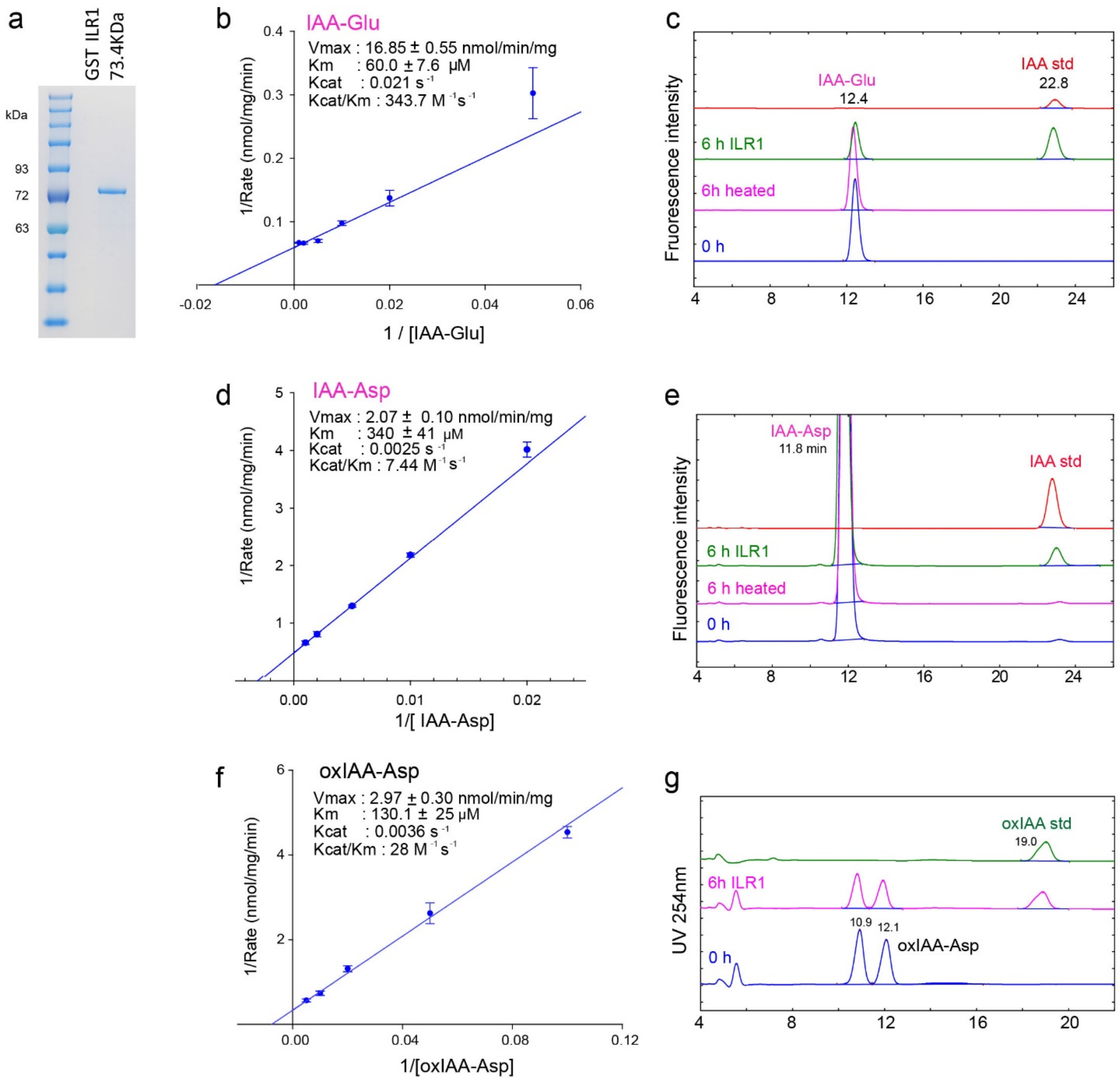

Supplementary Fig. 19. GST-ILR1 hydrolyzed both oxIAA-amino acid conjugates and IAA-amino acid conjugates.

(a) SDS-PAGE analysis of purified GST-ILR1 (73.4 kDa) protein expressed in a wheat germ cell-free protein expression system. This experiment was repeated three times independently with similar results. (b) Lineweaver–Burk plot and kinetics parameters of GST-ILR1 for oxIAA-Asp (n=3). (c) HPLC chromatogram of the reaction products of GST-ILR1. oxIAA-Asp was used as a substrate. (d) Lineweaver–Burk plot and kinetics parameters of GST-ILR1 for IAA-Glu (n=4). Error bars, SE. (e) HPLC chromatogram of the reaction products of GST-ILR1. IAA-Glu was used as a substrate. Error bars, SE. (f) Lineweaver–Burk plot and kinetics parameters of GST-ILR1 for IAA-Asp (n=4). Error bars, SE. (g) HPLC chromatogram of the reaction products of GST-ILR1. IAA-Asp was used as a substrate.

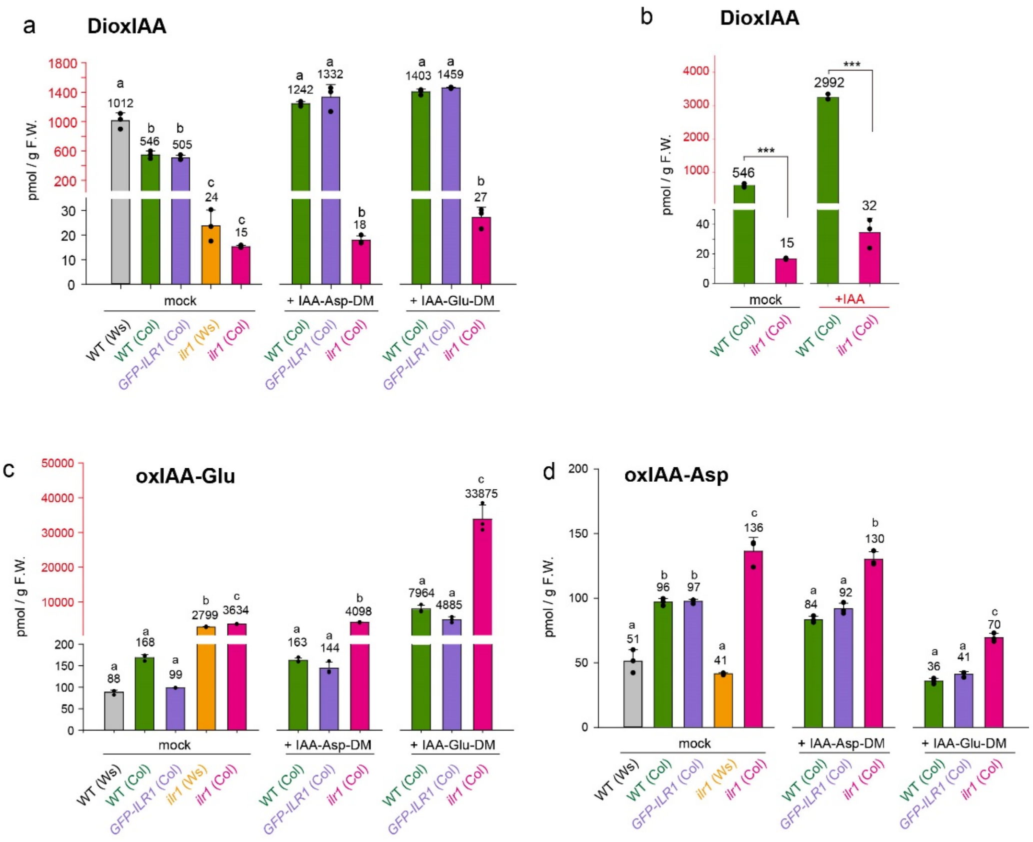

Supplementary Fig. 20. Endogenous amounts of IAA-Asp and dioxIAA in *ilr1* mutant and the complementation line.

(a–d) Seven-day-old seedlings of wild-type, *ilr1-1* (Ws, EMS mutant), *ilr1* (Col, SAIL_631_F01) and *pILR1::ILR1-GFP* in *ilr1* (Col) complementation lines were incubated in liquid GM media with or without 0.5 µM IAA, IAA-Asp-DM or IAA-Glu-DM for 24 h. After extensive washing of seedlings with fresh water, the metabolites [DioxIAA (a, b), oxIAA-Glu (c), oxIAA-Asp (d)] were analyzed with LC-MS/MS for the same sample in Fig. 6c. (a,c,d) The values shown are the means ± SD (n = 3). The different letters represent statistical significance at $P<0.001$ (Tukey's HSD test). (b) The values shown are the means ± SD (***$P < 0.001$, two-tailed Student's t-test, n = 3).

Supplementary Methods

Plasmid construction and transformation

Full-length cDNA clones of *AtDAO1*and *AtDAO2*, in this work was developed by the plant genome project of RIKEN Genomic Sciences Center (Japan) and cloned into the pDONR-ZEO entry vector by Gateway BP reaction. The genomic fragments of *gILR1* (3.9 kb), *gILL2* (3.2 kb), and *gIAR3* (4.3 kb), including the promoter regions (*pILR1*; 1.9 kb, *pILL2*; 1.4 kb, *pIAR*3; 2.4 kb), were amplified by KOD One (Toyobo, JAPAN) from *Arabidopsis* genomic DNA with the corresponding primers listed in Supplementary table 1. The amplified fragments were cloned into the pDONR-ZEO entry vector by Gateway BP reaction. The ORFs of *OsDAO* [*Oryza sativa*, Os04g0475600] and *BdDAO* [*Brachypodium distachyon*, XP_003579963] were synthesized and codon-optimized for expression *in Arabidopsis thaliana* and cloned into the pDONR-ZEO entry vector by BP clonase. To generate the β-estradiol-inducible constructs, AtDAO1 in pDONR-ZEO were cloned into the pMDC7 destination vector [1] by Gateway LR reaction. The pILR1::genomeILR1-GFP/GUS, pILL2::genomeILL2-GFP/GUS, and pIAR3::genomeIAR3-GFP/GUS constructs were generated by LR reaction of pDONR-proILR1::genomeILR1, pDONR-proILL2::genomeILL2, and pDONR-proIAR3::genomeIAR3 with pGWB504 and pGWB533 C-terminal fusions destination vectors. 35S::GFP-AtDA1 and 35S::GFP-OsDAO were generated by LR reaction of pDONR-AtDAO1_ORF and pDONR-OsDAO_ORF with pGWB506 and 35S::N-terminal GFP fusion destination vectors[2].

35S::GFP-DAO2 and 35S::GFP-BdDAO were generated by LR reaction of pDONR-AtDAO2_ _ORF and pDONR- BdDAO_ORF with pMDC43 and 35S::N-terminal GFP fusion destination vectors. 35S::genomeILR1-GFP, 35S::genomeILL2-GFP, and 35S::genomeIAR3-GFP were generated by LR reaction of pDONR-genomeILR1, pDONR-genomeILL2 and pDONR-genomeIAR3 with pMDC83 and 35S::C-terminal GFP fusion destination vectors [1]. Constructs were introduced into *Agrobacterium tumefaciens* GV3101 (pMP90). Wild-type and mutant plants were transformed by the floral dip method [3], and homozygous lines were selected.

HPLC analysis of IAA metabolites

In HPLC analysis, IAA, IAA-Asp, IAA-Glu, IAA-Ala, and IAA-Leu were detected by a fluorescent detector (Ex 280 nm, Em 360 nm) and UV absorption detector (254 nm). The oxIAA, oxIAA-Asp, oxIAA-Glu, oxIAA-Ala, and oxIAA-Leu were detected with UV detector (absorption at 254 nm).

For the analysis of GST-ILR1 reaction, the oxIAA, oxIAA-Glu, and oxIAA-Asp were analyzed by Inertsil ODS-3 (ODS, 150 × 4.6 mmm ID, GL-Science, Japan) with 0.5 mL/min flow rate of the mobile phase [MeOH : H_2O=30 : 70 containing 10 mM H_3PO_4] in Fig.4a and Supplimentary Fig. 19c. The IAA, IAA-Asp, and IAA-Glu were analysed by a Inertsil ODS-3 (150 × 4.6 mmm ID) with 0.5 mL/min flow rate of the mobile phase [MeOH : H_2O=43 : 57 containing 10 mM H_3PO_4] in Supplimentary Fig. 19e and 19g.

For the analysis of AtDAO1 reaction, the oxIAA-Asp and IAA-Asp was analyzed by Cosmosil C_{18}-MS-II column (ODS, 150 × 4.6 mmm ID, Nacalai-tesque, Japan) with 0.5 mL/min flow rate of the mobile phase [MeOH : H_2O=30 : 70 containing 10 mM phosphoric acid] in Supplimentary Fig. 4a. The oxIAA-Glu and IAA-Glu were analyzed by Cosmosil C_{18}-MS-II column with 0.5 mL/min flow rate of the mobile phase [MeOH : H_2O=30 :70 containing 10 mM phosphoric acid] in Supplimentary Fig. 4d. The IAA-Ala and oxIAA-Ala were analyzed by Cosmosil C_{18}-MS-II column (ODS, 150 × 4.6 mmm ID, Nacalai-tesque, Japan) with 0.5 mL/min flow rate of mobile phase [MeOH : H_2O=60 : 40 containing 10 mM H_3PO_4], and the IAA-Leu and oxIAA-Leu were analyzed by Cosmosil C_{18}-MS-II column (150 × 4.6 mmm ID) with 0.5 mL/min flow rate of mobile phase [MeOH : H_2O=50 : 50 containing 10 mM H_3PO_4] in Supplimentary Fig. 4d. The IAA and oxIAA were analysed by a Inertsil ODS-3 (150 × 4.6 mmm ID) with 0.5 mL/min flow rate of the mobile phase [MeOH : H_2O=43 : 57 containing 10 mM H_3PO_4] in Supplimentary Fig. 4F. IAA-glucoside and IAA-Asp-dimethyl ester were separated by Inertsil ODS3 coulumn (150 × 4.6 mmm ID) and Cosmosil C_{18}-MS-II coulumn (150 × 4.6 mmm ID) with the eluates,

MeOH : H_2O=45 : 55 containing 10 mM H_3PO_4 and MeOH : H_2O=50 : 50 containing 10 mM H_3PO_4 (0.5 mL/min), respectively in fig. Supplimentary Fig. 4e.

For the analysis of OsDAO reaction, the oxIAA-Glu, oxIAA-Asp, IAA-Glu, and IAA-Asp were analyzed by InertSustain C18 (150 × 4.6 mmm ID, GL-Science, Japan) with 0.5 mL/ min flow rate of the mobile phase [MeOH : H_2O=25 : 75 containing 10 mM phosphoric acid] in Supplimentary Fig. 6a and 6b. The IAA-Ala, IAA-Leu, oxIAA-Ala and oxIAA-Leu were also analysed by InertSustain C18 (150 × 4.6 mmm ID) with 0.5 mL/min flow rate of the mobile phase [MeOH : H_2O=30 : 70 containing 10 mM H_3PO_4] in Supplimentary Fig. 6c and with 0.5 mL/min flow rate of the mobile phase [MeOH : H_2O=50 : 50 containing 10 mM H_3PO_4] in Supplimentary Fig. 6d. The IAA and oxIAA were analysed by InertSustain C18 (150 × 4.6 mmm ID) with 0.5 mL/min flow rate of the mobile phase [MeOH : H_2O=57 : 43 containing 10 mM H_3PO_4] in Supplimentary Fig. 6e and 6f.

Molecular docking

The crystal data of AtDAO1 structure (PDB ID: 6KWB) containing 2-oxoglutarate and Mg ion were edited by Discovery Studio visualizer (Dassalt System, France). The macromolecule file (PDBQT) of AtDAO1 was prepared by AutoDockTools (MGL Tools software, The Scripps Research Institute). The ligand structure was prepared by Chem3D software (PerkinElmer ChemOffice, USA). The docking calculation of IAA and IAA-amino acid conjugates on AtDAO1 were performed by AutoDock Vina docking software [4]. The calculated coordinates of AtDAO1 and substrate were visualized by Discovery Studio visualizer.

Histochemical GUS Analysis

For GUS histochemical analysis, the seedlings were washed with GUS staining buffer (100 mM sodium phosphate, pH 7.0, 10 mM EDTA, 0.5 mM $K_4Fe(CN)_6$, 0.5 mM $K_3Fe(CN)_6$, and 0.1% Triton X-100), transferred to GUS staining buffer containing 1 mM 5-bromo-4-chloro-3-indolyl-*β*-D-glucuronide and incubated at 37 °C until sufficient staining developed. After the tissue was sufficiently decolorized with 75% (vol/vol) ethanol to remove chlorophyll, individual representative plant tissues were photographed under a microscope (Olympus, SZX16) equipped with a camera.

Synthesis of chemicals

General experimental conditions.

^{1}H and ^{13}C-NMR spectra were recorded on ECS400 and ECZ400 spectrometers (JEOL, Japan). Chemical shifts are shown as δ values from TMS as the internal reference. Peak multiplicities are quoted in Hz. Mass spectra were measured on autoflex speed MALDI-TOF MS (Bruker, Japan), and Agilent 6420 Triple Quad LC-MS (Agilent Technologies, USA). Column chromatography was carried out on columns of silica gel 60 (230–400 mesh, Merck, Japan). All chemicals were purchased from Tokyo Chemical Industry Japan (Japan), FUJIFILM Wako Pure Chemical (Japan) and Sigma-Aldrich Japan (Japan) unless otherwise stated.

Synthesis of kakeimide

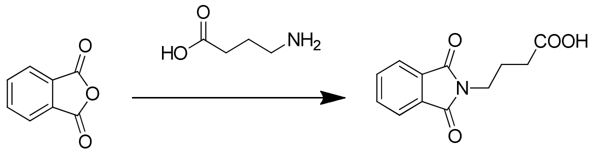

4-(1,3-dioxoisoindolin-2-yl)butanoic acid

4-(1,3-dioxoisoindolin-2-yl)butanoic acid [CAS 3130-75-4] was synthesized according to the method previously described [5]. The 4-(1,3-dioxoisoindolin-2-yl)butanoic acid was obtained as colorless powder (1358 mg, 75% yield). ^{1}H-NMR (400 MHz, $CDCl_3$) δ 7.85 (m, 2H), 7.72 (m, 2H), 3.77 (t, J = 6.9 Hz, 2H), 2.42 (t, J = 7.6 Hz, 2H), 2.05-1.98 (m, 2H); ^{13}C-NMR (100 MHz, $CDCl_3$) δ 178.5, 168.4, 134.0, 132.0, 123.3, 37.1, 31.3, 23.6.

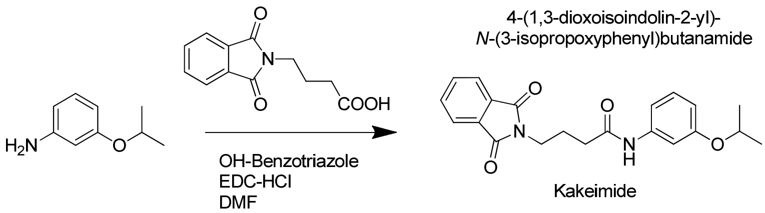

To the solution of 4-(1,3-dioxoisoindolin-2-yl) butanoic acid (940 mg, 4.0 mmol) in 8 mL of DMF was added 1-hydroxybenzotriazole monohydrate (545 mg, 4.0 mmol), 3-isopropoxyaniline (610 mg, 4.0 mmol), and EDC-HCl, 1-Ethyl-3-(3-dimethylaminopropyl)carbodiimide monohydrochloride (773 mg, 4.0mmol). The reaction mixture was stirred for 2 h at room temperature. The reaction mixture was poured into water (40 mL) and adjusted to pH 2-3 with 6M HCl. The mixture was extracted with EtOAc (30 mL × 2 times) and the EtOAc layer was then washed with 1 M Na_2CO_3 aqueous solution (30 mL). The EtOAc layer was dried over anhydrous Na_2SO_4 and then concentrated *in vacuo*. The resulting powder was recrystallized from *n*-hexane–EtOAc. The product, kakeimide, (4-(1,3-dioxoisoindolin-2-yl)-N-(3-isopropoxyphenyl) butanamide) was obtained as a colorless powder (1007 mg, 68% yield). m.p.= 113−115°C; ^{1}H-NMR (400 MHz, $CDCl_3$) δ 8.13 (s, 1H), 7.84 (m, 2H), 7.71 (m, 2H), 7.33 (s, 1H), 7.17 (t, J = 8.2 Hz, 1H), 7.02 (d, J = 8.7 Hz, 1H), 6.62 (d, J = 8.2 Hz, 1H), 4.56-4.50 (m, 1H), 3.81 (t, J = 6.2 Hz, 2H), 2.37 (t, J = 6.9 Hz, 2H), 2.12 (m, 2H), 1.32 (d, J = 6.0 Hz, 6H); ^{13}C-NMR (100 MHz, $CDCl_3$) δ 170.3, 168.9, 158.4, 139.2, 134.1, 131.9, 129.5, 123.3, 111.8, 111.6, 107.3, 69.8, 37.1, 35.0, 25.2, 22.0; MALDI-TOFMS $[M+Na]^+$ *m/z* calcd. for $C_{21}H_{22}N_2O_4Na$: 389.14773, found 389.14682.

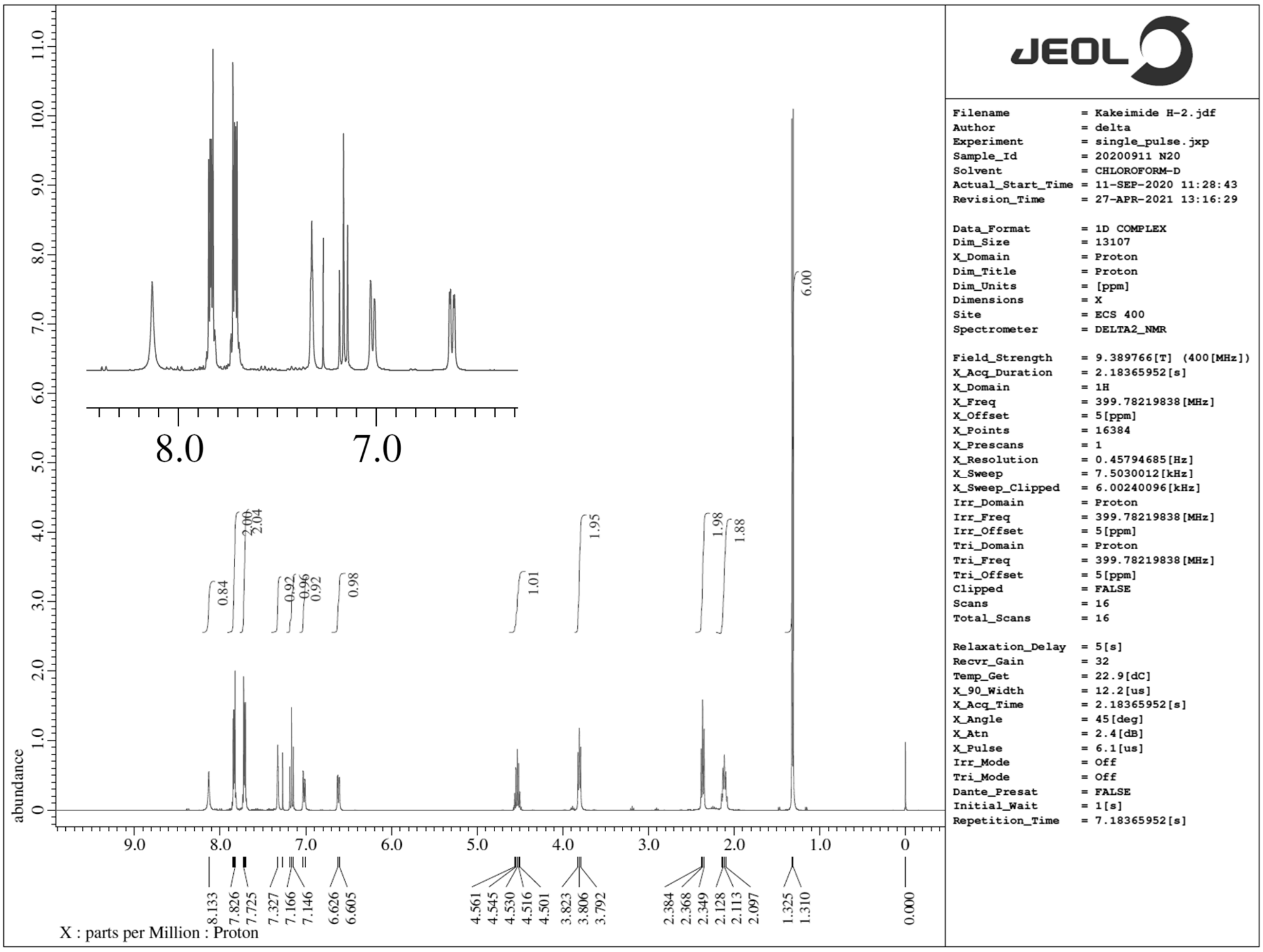

^{1}H-NMR-specrtum of kakeimide

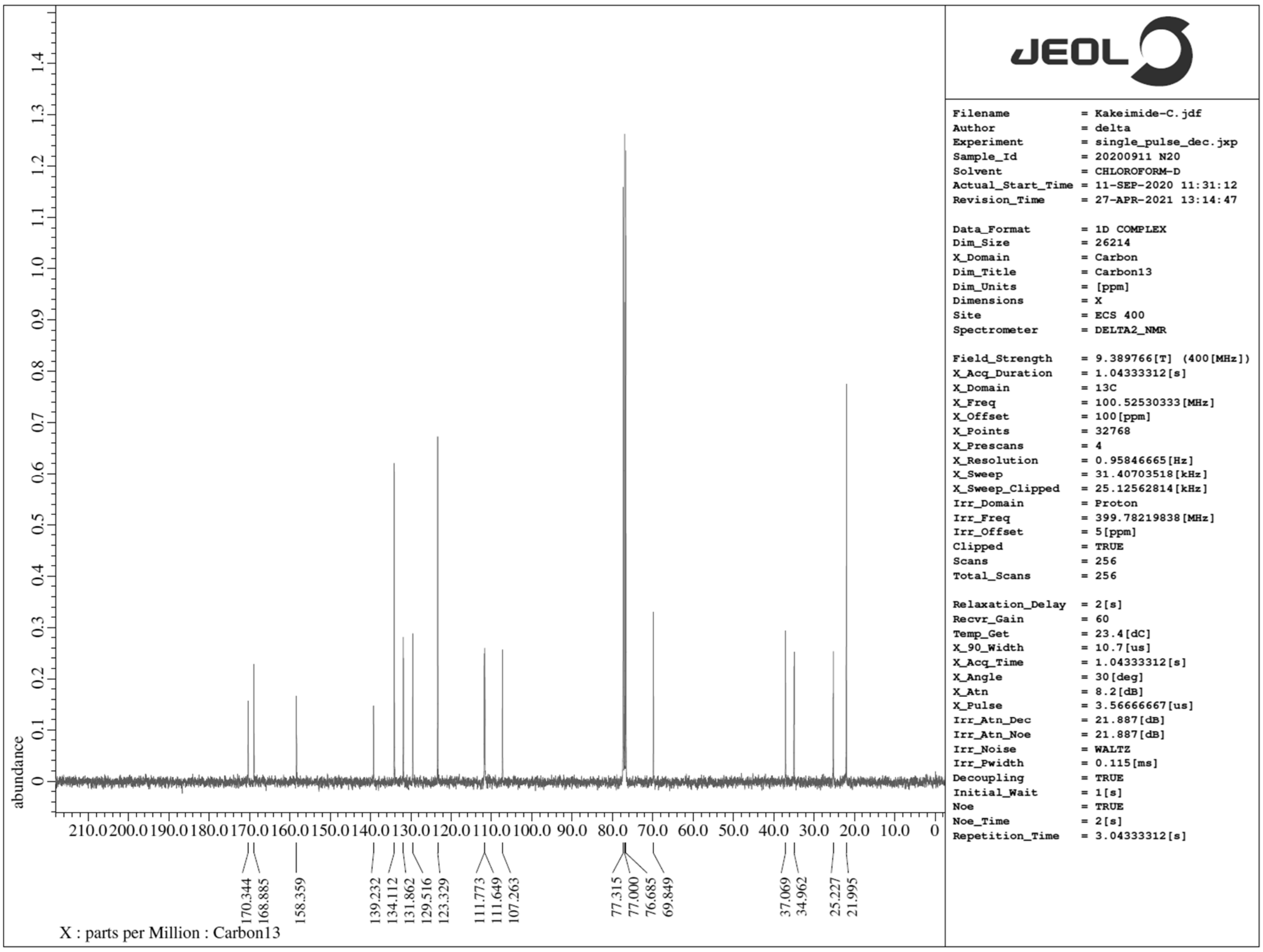

^{13}C-NMR-specrtum of kakeimide

Inhibitory activity of kakeimide on AtGH3.6

Kakeimide was found to be potent inhibitor specific to GH3 enzyme that catalyzed the conjugation of IAA with an amino acid. Kakeimde inhibited GH3 enzymatic activity in competition with IAA. Recombinant GH3.6 protein was purified by TALON metal affinity resin from the culture lysate of *E. coli* BL21 harboring pCold-GH3.6. The enzyme reaction was performed in the following assay condition; GH3.6 protein: 1.5 µg/mL, 50 mM Tris-HCl (pH 8.6), 3 mM $MgCl_2$, 3 mM ATP, various concentration of IAA, 3 mM *L*-aspartate, 1 mM dithiothreitol, 30 °C, 30 min [6]. The product, IAA-Asp was analyzed with HPLC system (EXTREMA, JASCO Japan). The Ki values for AtGH3.6 (Km=55.8 µM for IAA) was determined to be 48 nM （Supplementary table 3）according to the methods by Morrison and Cha [7, 8]. The detailed biological activity of kakeimide will be reported in the a separate manuscript.

Ki values of GH3 inhibitor, kakeimide on AtGH3.6

$$Ki = \frac{\text{Ki apparent}}{(1+\frac{[IAA]}{Km})}$$

IAA (µM)	$Ki^{apparent}$	Ki
70 µM	111 nM	49 nM
110 µM	143 nM	48 nM
250 µM	268 nM	49 nM
1000 µM	886 nM	47 nM
		Average Ki=48 nM

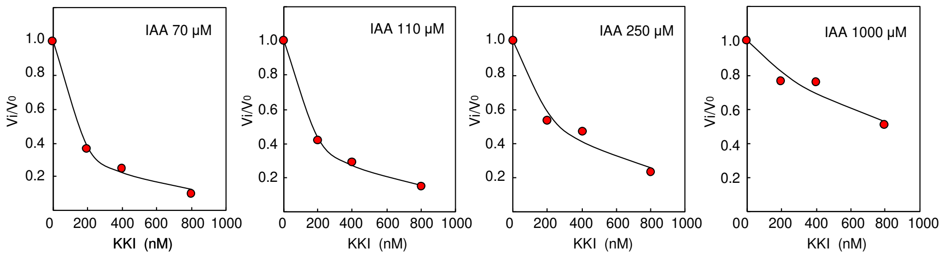

The Ki values of kakeimide on AtGH3.6 (Km=55.8 µM for IAA) was calculated from $Ki^{apparent}$ at different concentration of IAA according to the methods by Morrison and Cha [7, 8]. $Ki^{apparent}$ values were elevated in proportion to the concentration of IAA, indicating the inhibitory activity of kakeimide is competitive to IAA.

Synthesis of IAA metabolites.

The [phenyl-$^{13}C_6$]oxIAA, IAA-[$^{13}C_4$, ^{15}N]Asp, IAA-[$^{13}C_5$, ^{15}N]Glu, were previously synthesized [9, 10 11]. [2,2-$^2H_2$] (indol-3-ylacetyl)-ß-D-glucoside (2,2-$^2H_2$-IAA-glucoside) was synthesized as described previously [12]. [2,2-$^2H_2$]IAA-Ala and [2,2-$^2H_2$]IAA-Leu were synthesized by the coupling of [2,2-$^2H_2$]IAA with *L*-alanine methyl ester hydrochloride and *L*-leucine methyl ester hydrochloride, respectively, according to previously described method [9,13]. [2,2-$^2H_2$]DioxIAA, [2,2-$^2H_2$]oxIAA-Asp and [2,2-$^2H_2$]oxIAA-Glu were synthesized from [2,2-$^2H_2$]-oxIAA by the following procedure. The isotopic purity of these compounds was confirmed by LC–MS/MS analyses.

DioxIAA

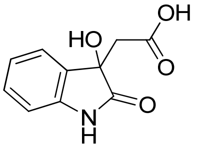

To the solution of oxIAA (38 mg, 0.19 mmol) in 2 mL of THF was added TEMPO, 2,2,6,6-tetramethylpiperidine 1-oxyl (15.4 mg, 0.098 mmol). The reaction mixture was then stirred for 48 h at room temperature. The reaction mixture was added to 0.1M HCl (10 mL), and extracted with EtOAc (15 mL × 3). The organic layer was washed with brine, and then dried over Na_2SO_4. The residue was purified by a silica gel column chromatography ($CHCl_3$:acetone=6:4, 0.2% AcOH) to give dioxIAA, 2-(3-hydroxy-2-oxoindolin-3-yl)acetic acid, (19 mg, 46% yield) as a yellowish solid. ^{1}H-NMR (400 MHz, -D$_6$): δ 9.49 (s, 1H), 7.43 (d, J = 7.3 Hz, 1H), 7.26 (td, J = 7.7, 1.1 Hz, 1H), 7.02-6.98 (m, 1H), 6.90 (d, J = 7.8 Hz, 1H), 3.10 (s, 2H, alpha $^2H_2$ position); ^{13}C-NMR (100 MHz, acetone-D$_6$): δ 175.6, 169.6, 144.7, 130.9, 128.1, 125.6, 122.5, 110.6, 84.3, 38.3; ESI-MS [M+H]$^+$ *m/z* 208.

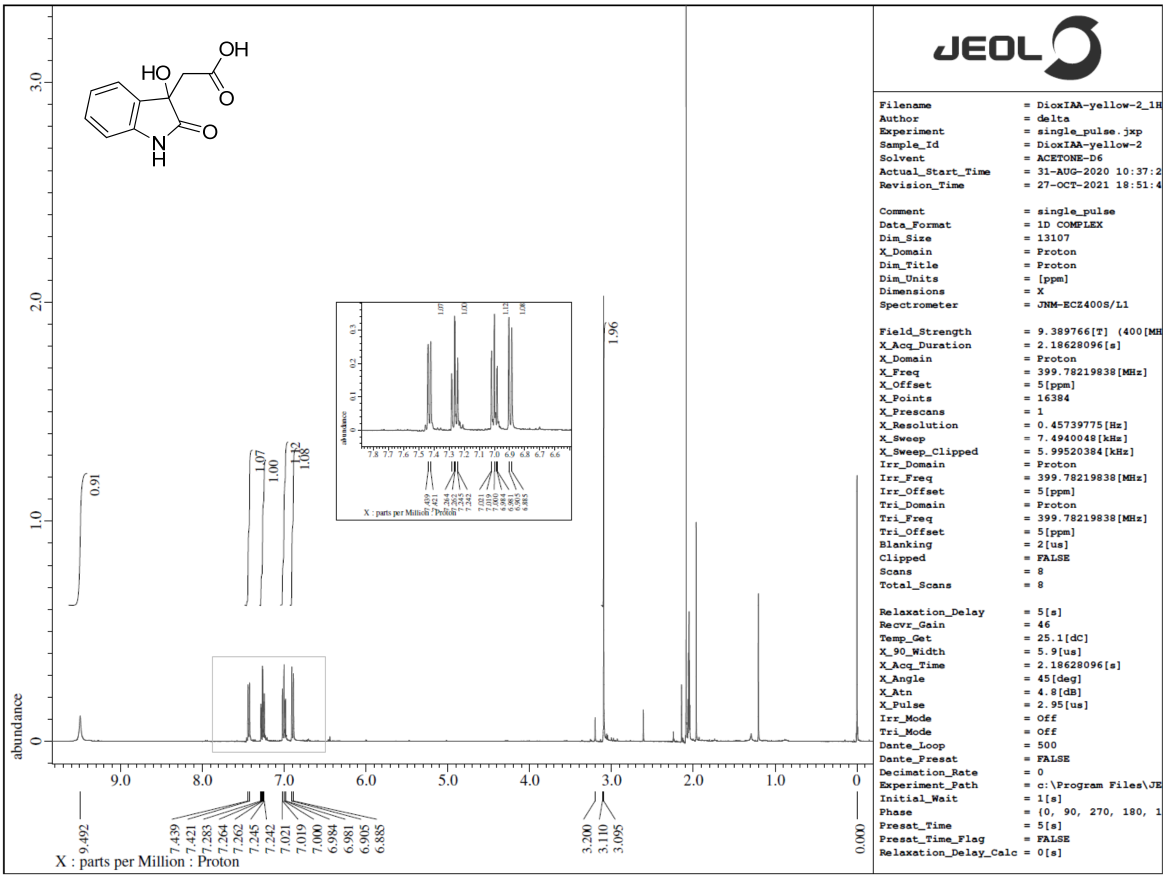

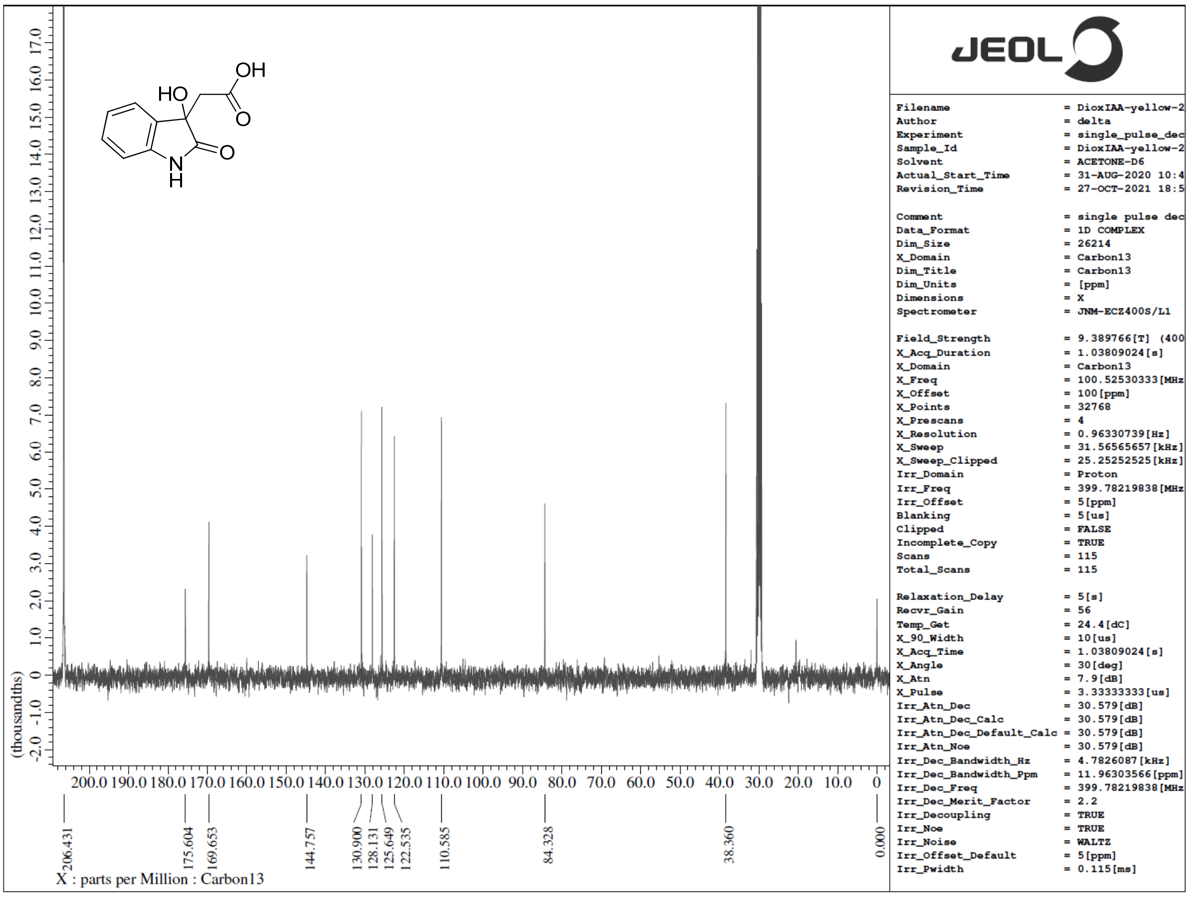
HO
OH
O
N
H
O
(thousandths)
17.0 16.0 15.0 14.0 13.0 12.0 11.0 10.0 9.0 8.0 7.0 6.0 5.0 4.0 3.0 2.0 1.0 0 -1.0 -2.0
200.0 190.0 180.0 170.0 160.0 150.0 140.0 130.0 120.0 110.0 100.0 90.0 80.0 70.0 60.0 50.0 40.0 30.0 20.0 10.0 0
206.431
175.604
169.653
144.757
130.900
128.131
125.649
122.535
110.585
84.328
38.360
0.000
X : parts per Million : Carbon13
Filename = DioxIAA-yellow-2
Author = delta
Experiment = single_pulse_dec
Sample_Id = DioxIAA-yellow-2
Solvent = ACETONE-D6
Actual_Start_Time = 31-AUG-2020 10:4
Revision_Time = 27-OCT-2021 18:5
Comment = single pulse dec
Data_Format = 1D COMPLEX
Dim_Size = 26214
X_Domain = Carbon13
Dim_Title = Carbon13
Dim_Units = [ppm]
Dimensions = X
Spectrometer = JNM-ECZ400S/L1
Field_Strength = 9.389766[T] (400
X_Acq_Duration = 1.03809024[s]
X_Domain = Carbon13
X_Freq = 100.52530333[MHz
X_Offset = 100[ppm]
X_Points = 32768
X_Prescans = 4
X_Resolution = 0.96330739[Hz]
X_Sweep = 31.56565657[kHz]
X_Sweep_Clipped = 25.25252525[kHz]
Irr_Domain = Proton
Irr_Freq = 399.78219838[MHz
Irr_Offset = 5[ppm]
Blanking = 5[us]
Clipped = FALSE
Incomplete_Copy = TRUE
Scans = 115
Total_Scans = 115
Relaxation_Delay = 5[s]
Recvr_Gain = 56
Temp_Get = 24.4[dC]
X_90_Width = 10[us]
X_Acq_Time = 1.03809024[s]
X_Angle = 30[deg]
X_Atn = 7.9[dB]
X_Pulse = 3.33333333[us]
Irr_Atn_Dec = 30.579[dB]
Irr_Atn_Dec_Calc = 30.579[dB]
Irr_Atn_Dec_Default_Calc = 30.579[dB]
Irr_Atn_Noe = 30.579[dB]
Irr_Dec_Bandwidth_Hz = 4.7826087[kHz]
Irr_Dec_Bandwidth_Ppm = 11.96303566[ppm]
Irr_Dec_Freq = 399.78219838[MHz
Irr_Dec_Merit_Factor = 2.2
Irr_Decoupling = TRUE
Irr_Noe = TRUE
Irr_Noise = WALTZ
Irr_Offset_Default = 5[ppm]
Irr_Pwidth = 0.115[ms]

oxIAA-*L*-Ala *tert*-butyl ester.

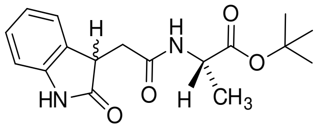

To the solution of oxIAA (200 mg, 1.00 mmol), L-alanine *tert*-butyl ester hydrochloride (229 mg, 1.00 mmol) and 1-hydroxybenzotriazole monohydrate (170 mg, 1.30 mmol) in 5 mL of DMF was added EDC-HCl (15.4 mg, 0.098 mmol) and 4-dimethylaminopyridine (DMAP, 153 mg, 1.30 mmol). The reaction mixture was then stirred for 15 h at room temperature. The reaction mixture was added to water (50 mL) and acidified to pH 3.0, and extracted with EtOAc (50 mL × 3). EtOAc layer was then washed with aqueous 0.25M Na_2CO_3 solution (30 mL). The organic layer was washed with brine, and then dried over Na_2SO_4. The residue was purified by a silica gel column chromatography (hexane:acetone=3:2) to give a diastereomeric mixture (5 : 4) of oxIAA-*L*-Ala *tert*-butyl ester at C-3 position (247 mg, 74 % yield) as a pale yellow solid. ^{1}H-NMR (400 MHz, $CDCl_3$) δ 8.60 (s, 1H), 8.53 (s, 1H), 7.24-7.16 (m, 4H), 6.99 (q, J = 7.3 Hz, 2H), 6.88 (t, J = 7.1 Hz, 2H), 6.83 (d, J = 7.1 Hz, 1H), 6.77 (d, J = 7.1 Hz, 1H), 4.52-4.47 (m, 2H), 3.93-3.89 (m, 2H), 2.98 (dd, J = 15.6, 5.5 Hz, 1H), 2.92 (dd, J = 15.6, 5.5 Hz, 1H), 2.64 (d, J = 7.8 Hz, 1H), 2.60 (d, J = 7.8 Hz, 1H), 1.48 (s, 9H), 1.46 (s, 9H), 1.39 (d, J = 6.9 Hz, 3H), 1.36 (d, J = 6.9 Hz, 3H). The signal integration was adjusted to 1H from each diastereomer; ^{13}C-NMR (100 MHz, $CDCl_3$) δ 179.8, 172.6, 172.4, 169.6, 169.5, 141.3, 129.1, 128.3, 124.7, 124.6, 122.6, 109.8, 82.2, 49.0, 42.6, 37.0, 37.0, 28.0, 18.6; MALDI-TOFMS $[M+Na]^+$ *m/z* 341.15.

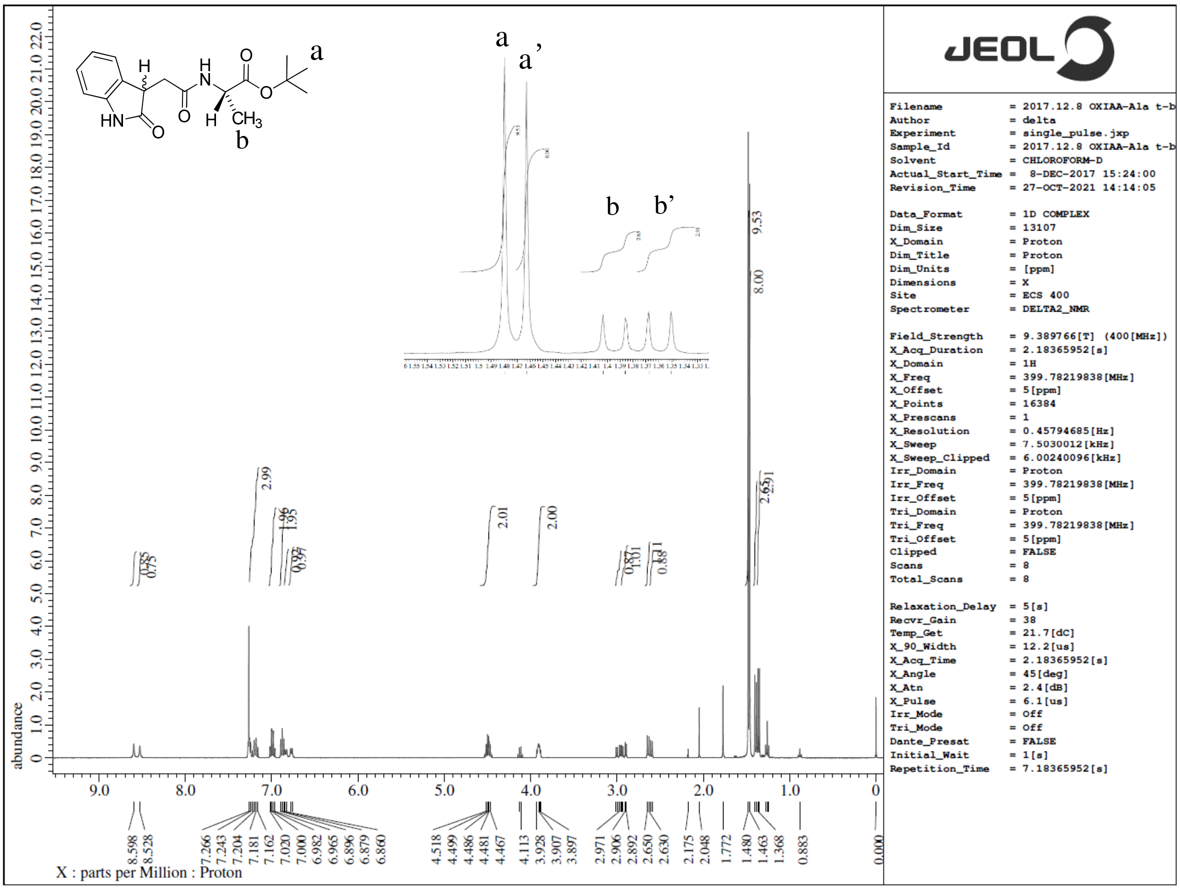

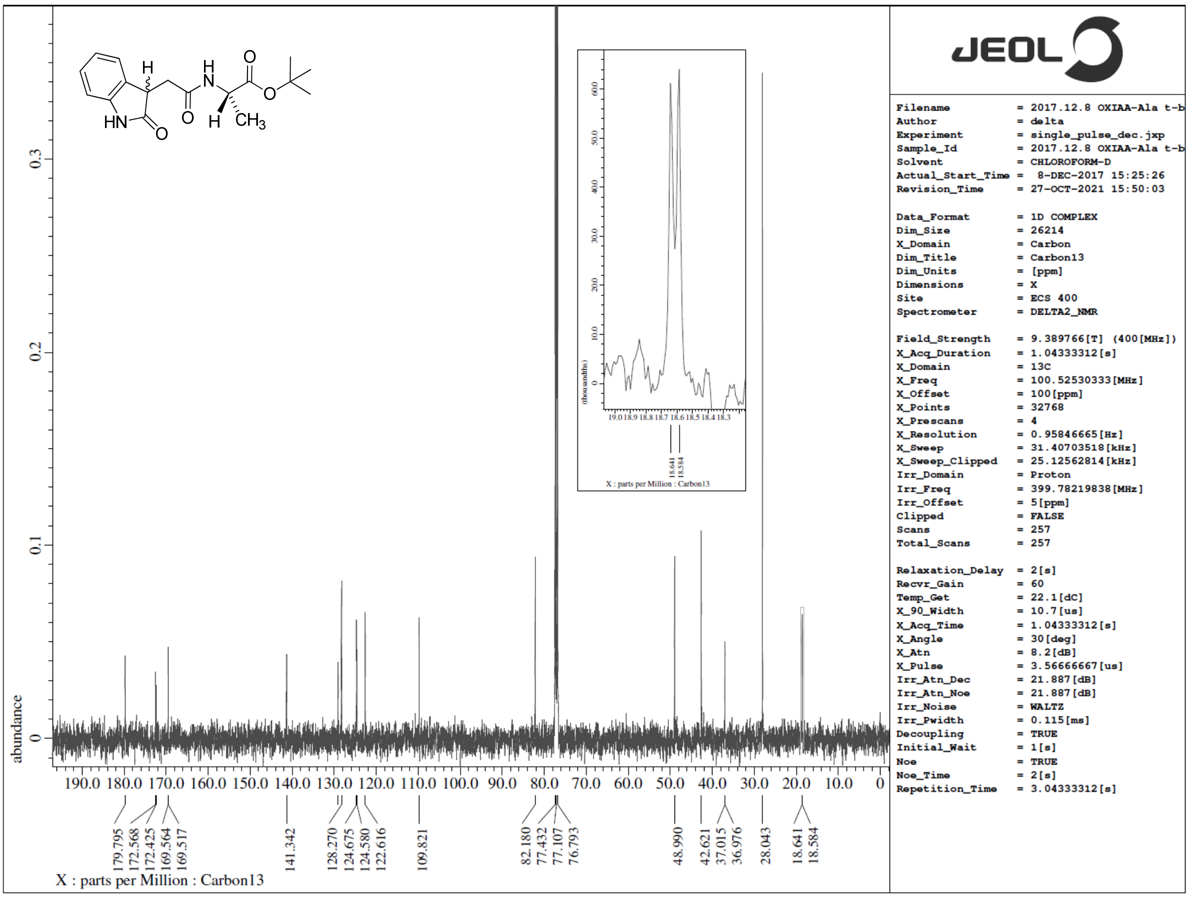

HN
O
H
N
O
H
O
O
H
CH3
abundance
0
0.1
0.2
0.3
190.0 180.0 170.0 160.0 150.0 140.0 130.0 120.0 110.0 100.0 90.0 80.0 70.0 60.0 50.0 40.0 30.0 20.0 10.0 0
179.795
172.568
172.425
169.564
169.517
141.342
128.270
124.675
124.580
122.616
109.821
82.180
77.432
77.107
76.793
48.990
42.621
37.015
36.976
28.043
18.641
18.584
X : parts per Million : Carbon13
(thousandths)
19.0 18.9 18.8 18.7 18.6 18.5 18.4 18.3
18.641
18.584
X : parts per Million : Carbon13
JEOL
Filename = 2017.12.8 OXIAA-Ala t-b
Author = delta
Experiment = single_pulse_dec.jxp
Sample_Id = 2017.12.8 OXIAA-Ala t-b
Solvent = CHLOROFORM-D
Actual_Start_Time = 8-DEC-2017 15:25:26
Revision_Time = 27-OCT-2021 15:50:03
Data_Format = 1D COMPLEX
Dim_Size = 26214
X_Domain = Carbon
Dim_Title = Carbon13
Dim_Units = [ppm]
Dimensions = X
Site = ECS 400
Spectrometer = DELTA2_NMR
Field_Strength = 9.389766[T] (400[MHz])
X_Acq_Duration = 1.04333312[s]
X_Domain = 13C
X_Freq = 100.52530333[MHz]
X_Offset = 100[ppm]
X_Points = 32768
X_Prescans = 4
X_Resolution = 0.95846665[Hz]
X_Sweep = 31.40703518[kHz]
X_Sweep_Clipped = 25.12562814[kHz]
Irr_Domain = Proton
Irr_Freq = 399.78219838[MHz]
Irr_Offset = 5[ppm]
Clipped = FALSE
Scans = 257
Total_Scans = 257
Relaxation_Delay = 2[s]
Recvr_Gain = 60
Temp_Get = 22.1[dC]
X_90_Width = 10.7[us]
X_Acq_Time = 1.04333312[s]
X_Angle = 30[deg]
X_Atn = 8.2[dB]
X_Pulse = 3.56666667[us]
Irr_Atn_Dec = 21.887[dB]
Irr_Atn_Noe = 21.887[dB]
Irr_Noise = WALTZ
Irr_Pwidth = 0.115[ms]
Decoupling = TRUE
Initial_Wait = 1[s]
Noe = TRUE
Noe_Time = 2[s]
Repetition_Time = 3.04333312[s]

oxIAA-*L*-Ala

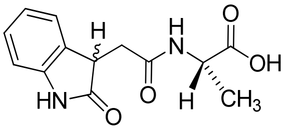

Trifluoroacetic acid (1.5 mL) and triisopropylsilane (0.04 mL) were added to oxIAA-*L*-Ala *tert*-butyl ester (200 mg, 0.60 mmol) in a screw capped glass vial. The reaction mixture was then stirred for 1 h at room temperature. The reaction mixture was dropped into water (30 mL) and extracted with EtOAc (50 mL × 3). The organic layer was washed with brine, and then dried over Na_2SO_4. The residue was purified by a silica gel column chromatography ($CHCl_3$:MeOH=9:1, 0.5% AcOH) to give oxIAA-*L*-Ala (90 mg, 55% yield) as a pale yellow solid. OxIAA-Ala was obtained as a diastereomeric mixture (10 : 9) at C-3 due to the rapid epimerization at C-3.^{1}H-NMR (400 MHz, CD_3OD) δ 7.32 (d, J = 7.3 Hz, 1H), 7.19 (m, 3H), 6.96 (dd, J = 8.0, 7.1 Hz, 2H), 6.88 (d, J = 7.8 Hz, 1H), 6.86 (d, J = 7.8 Hz, 1H), 4.40 (m, 2H), 3.84-3.80 (m, 2H), 2.98 (dd, J = 15.3, 4.4 Hz, 1H), 2.92 (dd, J = 15.1, 4.6 Hz, 1H), 2.61-2.56 (m, 1H), 2.55-2.50 (m, 1H), 1.39 (d, J = 7.3 Hz, 3H), 1.35 (d, J = 7.3 Hz, 3H). The signal integration was adjusted to 1H from each diastereomer; ^{13}C-NMR (100 MHz, CD_3OD) δ 180.2, 174.6, 171.2, 142.1, 129.3, 129.2, 127.9, 127.8, 124.3, 124.0, 121.9, 109.5, 109.4, 42.8, 42.6, 35.9, 35.7, 16.4, 16.2; ESI-MS $[M+H]^+$ *m/z* 263.

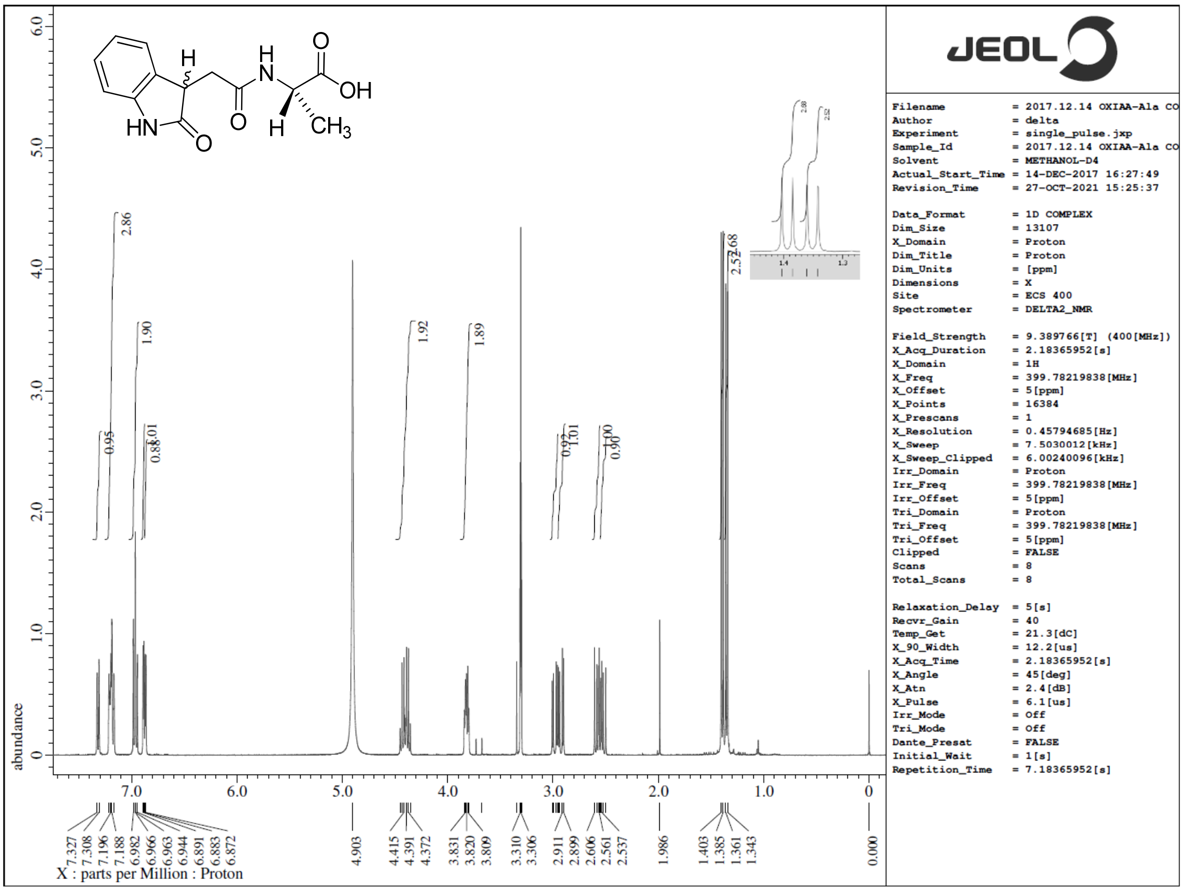

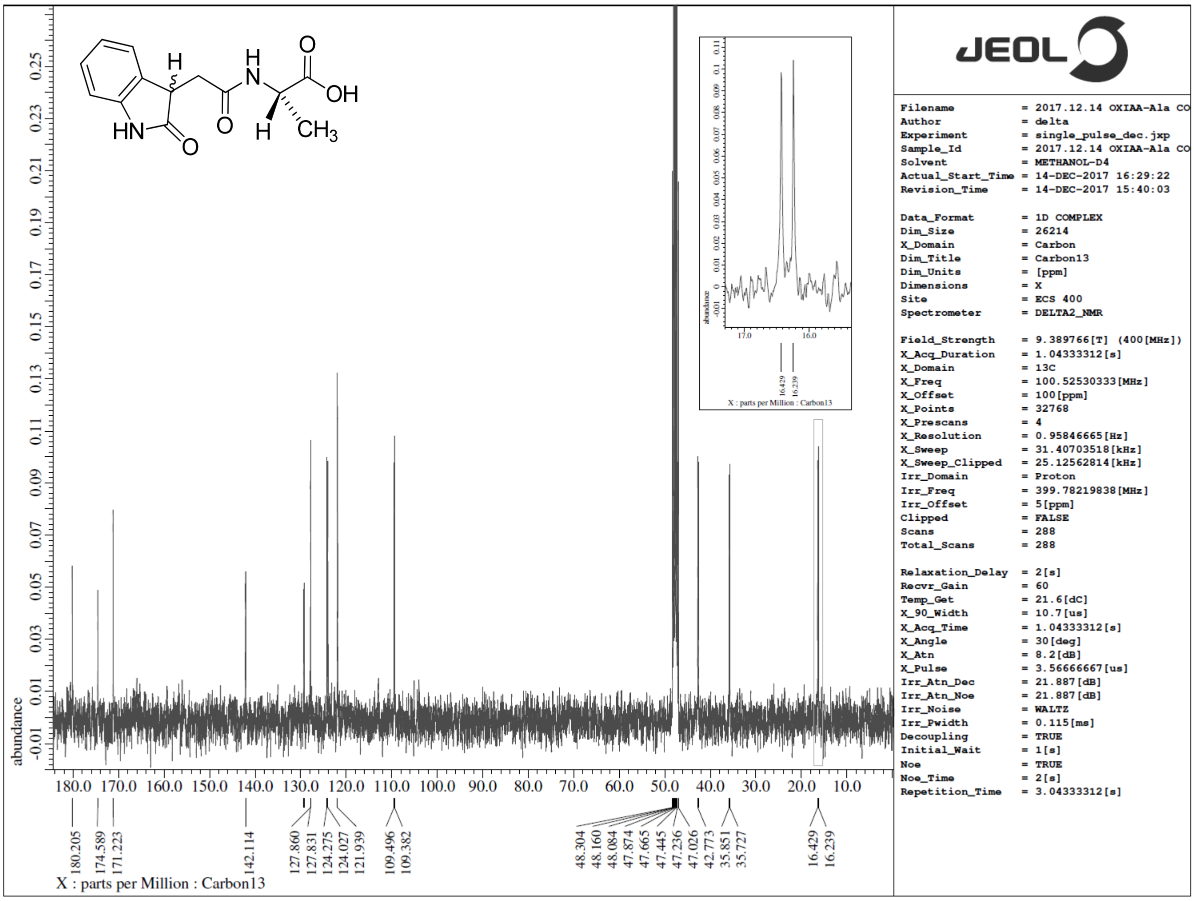
Filename = 2017.12.14 OXIAA-Ala CO
Author = delta
Experiment = single_pulse_dec.jxp
Sample_Id = 2017.12.14 OXIAA-Ala CO
Solvent = METHANOL-D4
Actual_Start_Time = 14-DEC-2017 16:29:22
Revision_Time = 14-DEC-2017 15:40:03
Data_Format = 1D COMPLEX
Dim_Size = 26214
X_Domain = Carbon
Dim_Title = Carbon13
Dim_Units = [ppm]
Dimensions = X
Site = ECS 400
Spectrometer = DELTA2_NMR
Field_Strength = 9.389766[T] (400[MHz])
X_Acq_Duration = 1.04333312[s]
X_Domain = 13C
X_Freq = 100.52530333[MHz]
X_Offset = 100[ppm]
X_Points = 32768
X_Prescans = 4
X_Resolution = 0.95846665[Hz]
X_Sweep = 31.40703518[kHz]
X_Sweep_Clipped = 25.12562814[kHz]
Irr_Domain = Proton
Irr_Freq = 399.78219838[MHz]
Irr_Offset = 5[ppm]
Clipped = FALSE
Scans = 288
Total_Scans = 288
Relaxation_Delay = 2[s]
Recvr_Gain = 60
Temp_Get = 21.6[dC]
X_90_Width = 10.7[us]
X_Acq_Time = 1.04333312[s]
X_Angle = 30[deg]
X_Atn = 8.2[dB]
X_Pulse = 3.56666667[us]
Irr_Atn_Dec = 21.887[dB]
Irr_Atn_Noe = 21.887[dB]
Irr_Noise = WALTZ
Irr_Pwidth = 0.115[ms]
Decoupling = TRUE
Initial_Wait = 1[s]
Noe = TRUE
Noe_Time = 2[s]
Repetition_Time = 3.04333312[s]
180.205 174.589 171.223 142.114 127.860 127.831 124.275 124.027 121.939 109.496 109.382 48.304 48.160 48.084 47.874 47.665 47.445 47.236 47.026 42.773 35.851 35.727 16.429 16.239
X : parts per Million : Carbon13
abundance

oxIAA-*L*-Leu *tert*-butyl ester

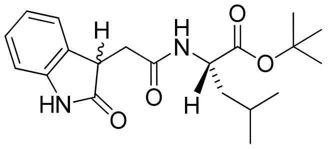

oxIAA-*L*-Leu *tert*-butyl ester was synthesized with the same procedure for oxIAA-*L*-Ala *tert*-butyl ester. oxIAA-*L*-Leu *tert*-butyl ester was obtained as a diastereomeric mixture (10 : 9) of oxIAA-*L*-Leu *tert*-butyl ester at C-3 position (285 mg, 76 % yield) as a pale brownish solid. ^{1}H-NMR (400 MHz, $CDCl_3$) δ 8.44 (s, 1H), 8.36 (s, 1H), 7.26 (s,2H), 7.23-7.16 (m, 2H), 6.99 (q, J = 7.9 Hz, 2H), 6.87 (t, J = 8.5 Hz, 2H), 6.72 (d, J = 8.2 Hz, 1H), 6.61 (d, J = 8.2 Hz, 1H), 4.55-4.52 (m, 2H), 3.91 (m, 2H), 3.02-2.90 (m, 2H), 2.66-2.59 (m, 2H), 1.70-1.51 (m, 8H), 1.48 (s, 9H), 1.46 (s, 9H), 0.96-0.90 (m, 12H). The signal integration was adjusted to 1H from each diastereomer; ^{13}C-NMR (100 MHz, $CDCl_3$) δ 179.8, 179.7, 172.5, 169.8, 141.3, 141.2, 129.0, 128.3, 124.7, 122.7, 122.6, 109.8, 82.1, 51.7, 42.7, 41.9, 37.1, 28.1, 25.0, 22.9, 22.2; MALD-TOFMS [M+Na]$^+$ *m/z* 383.183

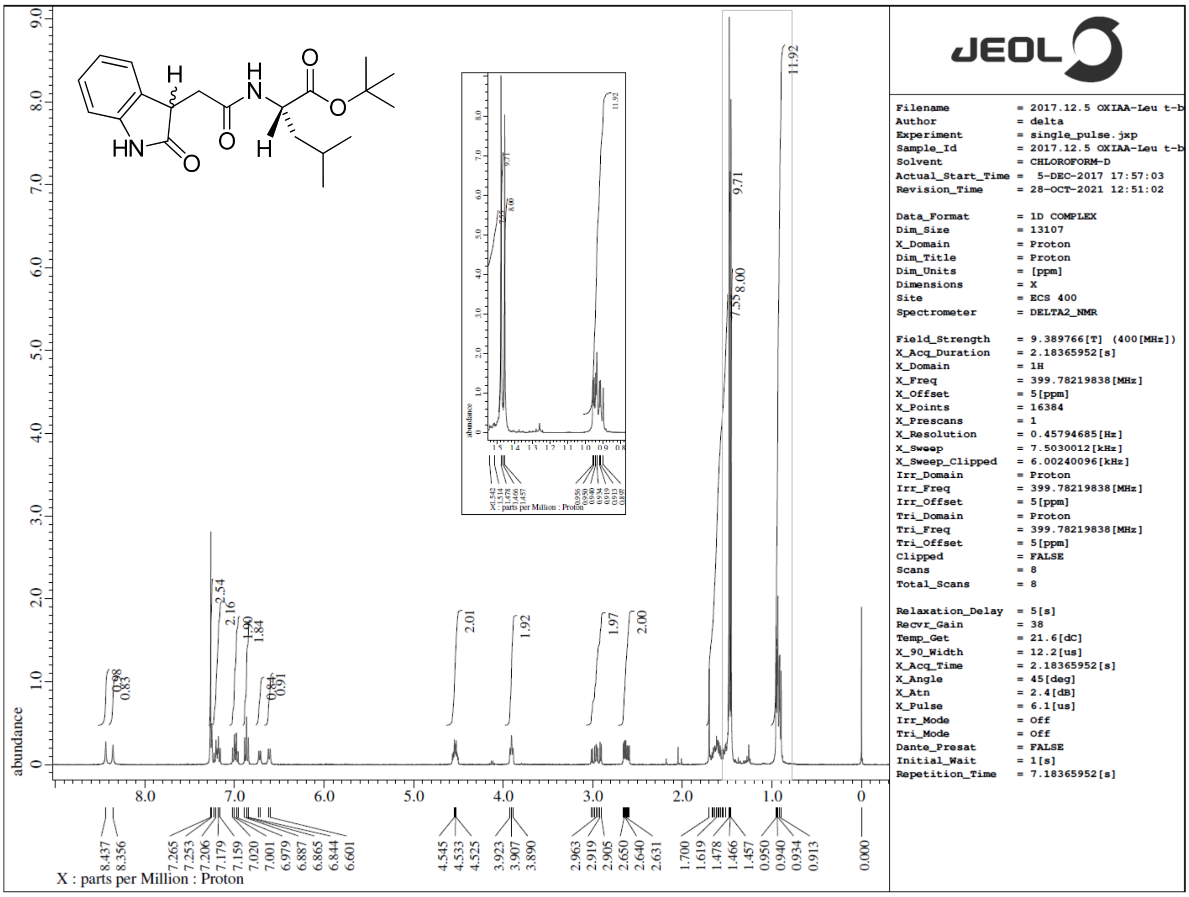

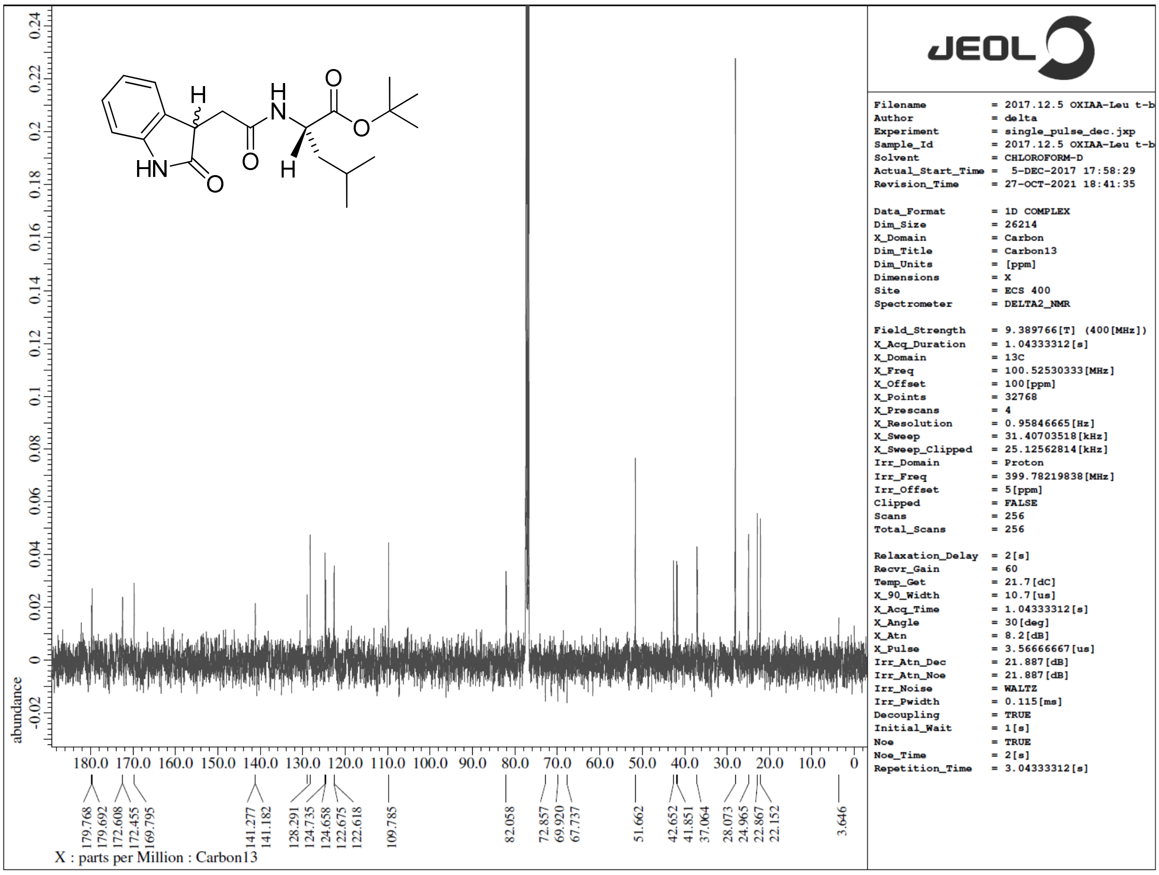
Filename = 2017.12.5 OXIAA-Leu t-b
Author = delta
Experiment = single_pulse_dec.jxp
Sample_Id = 2017.12.5 OXIAA-Leu t-b
Solvent = CHLOROFORM-D
Actual_Start_Time = 5-DEC-2017 17:58:29
Revision_Time = 27-OCT-2021 18:41:35
Data_Format = 1D COMPLEX
Dim_Size = 26214
X_Domain = Carbon
Dim_Title = Carbon13
Dim_Units = [ppm]
Dimensions = X
Site = ECS 400
Spectrometer = DELTA2_NMR
Field_Strength = 9.389766[T] (400[MHz])
X_Acq_Duration = 1.04333312[s]
X_Domain = 13C
X_Freq = 100.52530333[MHz]
X_Offset = 100[ppm]
X_Points = 32768
X_Prescans = 4
X_Resolution = 0.95846665[Hz]
X_Sweep = 31.40703518[kHz]
X_Sweep_Clipped = 25.12562814[kHz]
Irr_Domain = Proton
Irr_Freq = 399.78219838[MHz]
Irr_Offset = 5[ppm]
Clipped = FALSE
Scans = 256
Total_Scans = 256
Relaxation_Delay = 2[s]
Recvr_Gain = 60
Temp_Get = 21.7[dC]
X_90_Width = 10.7[us]
X_Acq_Time = 1.04333312[s]
X_Angle = 30[deg]
X_Atn = 8.2[dB]
X_Pulse = 3.56666667[us]
Irr_Atn_Dec = 21.887[dB]
Irr_Atn_Noe = 21.887[dB]
Irr_Noise = WALTZ
Irr_Pwidth = 0.115[ms]
Decoupling = TRUE
Initial_Wait = 1[s]
Noe = TRUE
Noe_Time = 2[s]
Repetition_Time = 3.04333312[s]
179.768 179.692 172.608 172.455 169.795 141.277 141.182 128.291 124.735 124.658 122.675 122.618 109.785 82.058 72.857 69.920 67.737 51.662 42.652 41.851 37.064 28.073 24.965 22.867 22.152 3.646
abundance
X : parts per Million : Carbon13

oxIAA-*L*-Leu

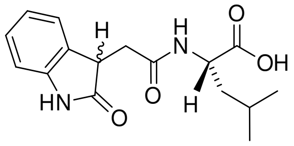

oxIAA-*L*-Leu was synthesized with the same procedure for oxIAA- *L*-Ala. oxIAA-*L*-Leu was obtained as a diastereomeric mixture (10 : 9) of oxIAA-*L*-Leu at C-3 position (139 mg, 82 % yield) as a pale yellow solid. ^{1}H-NMR (400 MHz, CD_3OD) δ 7.29 (d, J = 7.3 Hz, 1H), 7.19 (m, 3H), 6.95 (m, 2H), 6.88 (t, J = 7.1 Hz, 2H), 4.49-4.38 (m, 2H), 3.84-3.78 (m, 2H), 3.02-2.92 (m, 2H), 2.64 (q, J = 7.8 Hz, 1H), 2.53 (dd, J = 15.1, 9.6 Hz, 1H), 1.74-1.53 (m, 6H), 0.97-0.91 (m, 9H), 0.83-0.75 (d, J = 6.0, 3H). The signal integration was adjusted to 1H from each diastereomer; ^{13}C-NMR (100 MHz, CD_3OD) δ 181.6, 181.5, 175.9, 172.9, 172.8, 143.6, 143.5, 130.6, 130.4, 129.3, 129.2, 125.5, 125.3, 123.2, 123.2, 110.9, 110.7, 52.1, 44.2, 44.0, 41.5, 37.3, 37.0, 25.9, 25.9, 23.4, 21.7, 21.6; ESI-MS $[M+H]^+$ *m/z* 305.

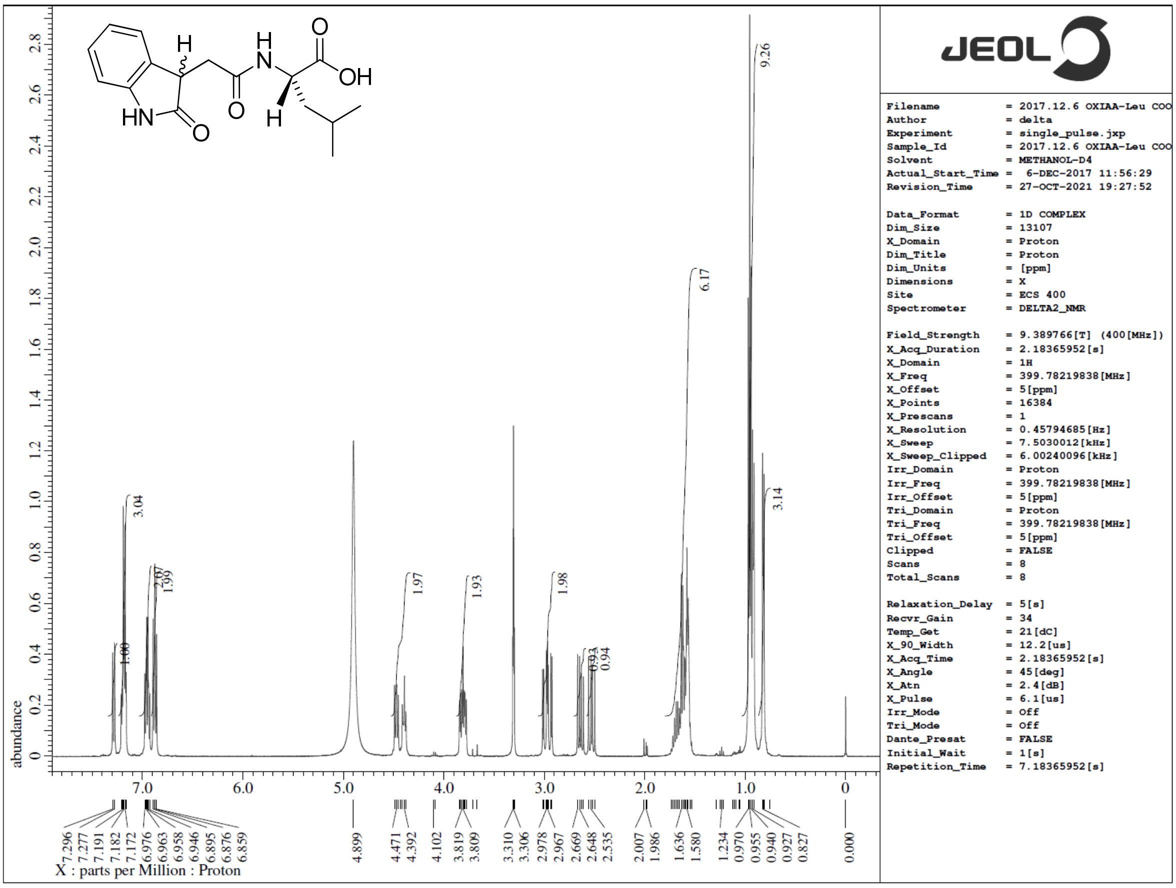

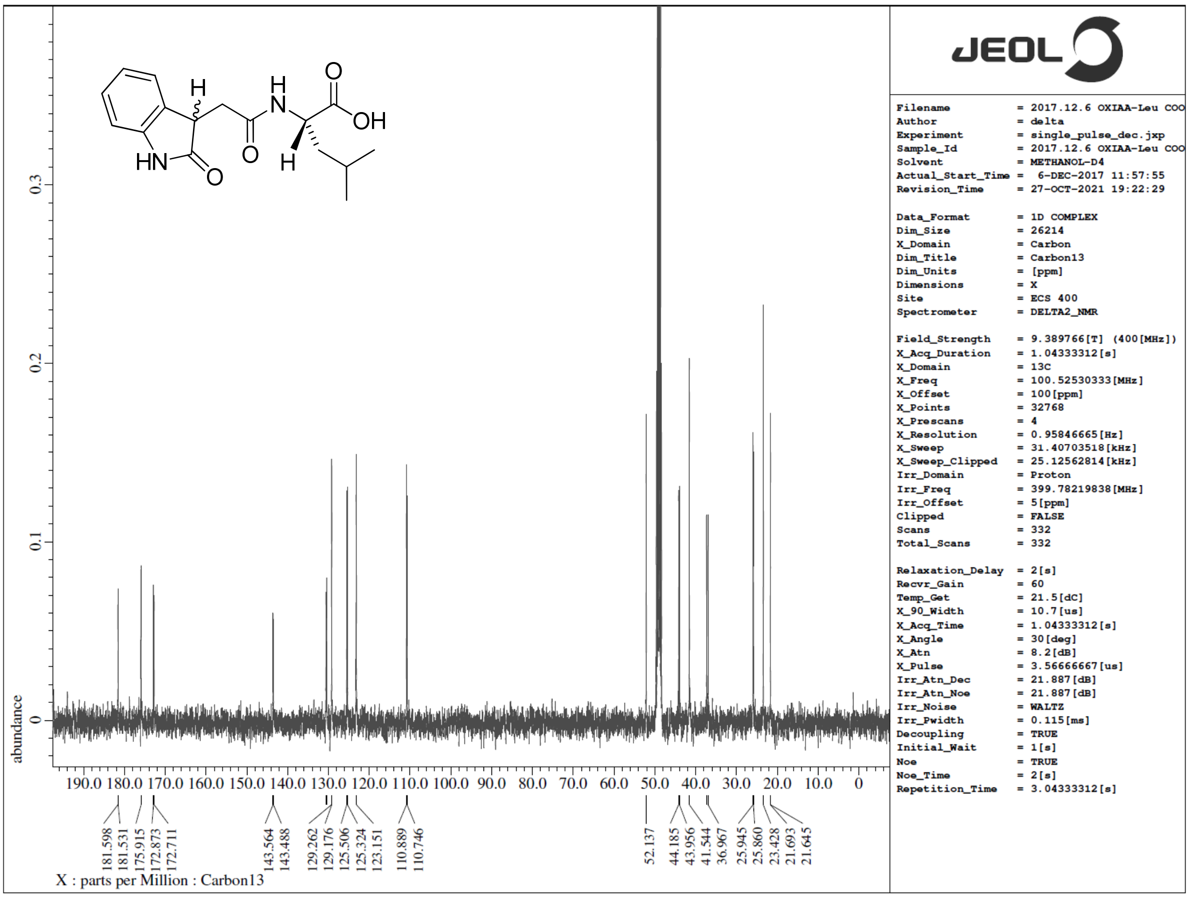

JEOL
Filename = 2017.12.6 OXIAA-Leu COO
Author = delta
Experiment = single_pulse_dec.jxp
Sample_Id = 2017.12.6 OXIAA-Leu COO
Solvent = METHANOL-D4
Actual_Start_Time = 6-DEC-2017 11:57:55
Revision_Time = 27-OCT-2021 19:22:29
Data_Format = 1D COMPLEX
Dim_Size = 26214
X_Domain = Carbon
Dim_Title = Carbon13
Dim_Units = [ppm]
Dimensions = X
Site = ECS 400
Spectrometer = DELTA2_NMR
Field_Strength = 9.389766[T] (400[MHz])
X_Acq_Duration = 1.04333312[s]
X_Domain = 13C
X_Freq = 100.52530333[MHz]
X_Offset = 100[ppm]
X_Points = 32768
X_Prescans = 4
X_Resolution = 0.95846665[Hz]
X_Sweep = 31.40703518[kHz]
X_Sweep_Clipped = 25.12562814[kHz]
Irr_Domain = Proton
Irr_Freq = 399.78219838[MHz]
Irr_Offset = 5[ppm]
Clipped = FALSE
Scans = 332
Total_Scans = 332
Relaxation_Delay = 2[s]
Recvr_Gain = 60
Temp_Get = 21.5[dC]
X_90_Width = 10.7[us]
X_Acq_Time = 1.04333312[s]
X_Angle = 30[deg]
X_Atn = 8.2[dB]
X_Pulse = 3.56666667[us]
Irr_Atn_Dec = 21.887[dB]
Irr_Atn_Noe = 21.887[dB]
Irr_Noise = WALTZ
Irr_Pwidth = 0.115[ms]
Decoupling = TRUE
Initial_Wait = 1[s]
Noe = TRUE
Noe_Time = 2[s]
Repetition_Time = 3.04333312[s]
abundance
0
0.1
0.2
0.3
190.0 180.0 170.0 160.0 150.0 140.0 130.0 120.0 110.0 100.0 90.0 80.0 70.0 60.0 50.0 40.0 30.0 20.0 10.0 0
181.598
181.531
175.915
172.873
172.711
143.564
143.488
129.262
129.176
125.506
125.324
123.151
110.889
110.746
52.137
44.185
43.956
41.544
36.967
25.945
25.860
23.428
21.693
21.645
X : parts per Million : Carbon13
HN
O
H
H
N
O
OH
O
H

oxIAA-*L*-Asp *tert*-butyl diester

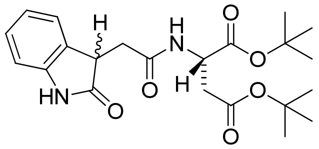

oxIAA-*L*-Asp *tert*-butyl diester was synthesized with the same procedure for oxIAA-*L*-Ala *tert*-butyl ester. oxIAA-*L*-Asp *tert*-butyl diester was obtained as a diastereomeric mixture (10 : 9) of oxIAA-*L*-Asp *tert*-butyl diester at C-3 position (210 mg, 79% yield) as a pale yellow solid. ^{1}H-NMR (400 MHz, $CDCl_3$) δ 8.31 (s, 2H), 7.29-7.17 (m, 4H), 7.00-6.95 (m, 4H), 6.88 (d, J = 7.8 Hz, 2H), 4.72 (m, 2H), 3.91 (m, 2H), 3.06-2.84 (m, 4H), 2.79-2.60 (m, 4H), 1.46 (s, 9H), 1.45 (s, 9H), 1.44 (s, 9H), 1.43 (s, 9H). The signal integration was adjusted to 1H from each diastereomer ; ^{13}C-NMR (100 MHz, $CDCl_3$) δ 179.5, 170.5, 170.3, 169.8, 141.3, 129.1, 128.2, 124.8, 124.6, 122.6, 122.6, 109.8, 82.5, 82.4, 81.7, 49.4, 42.5, 37.5, 36.9, 36.8, 28.1, 28.0; ESI-MS $[M+H]^+$ *m/z* 419.

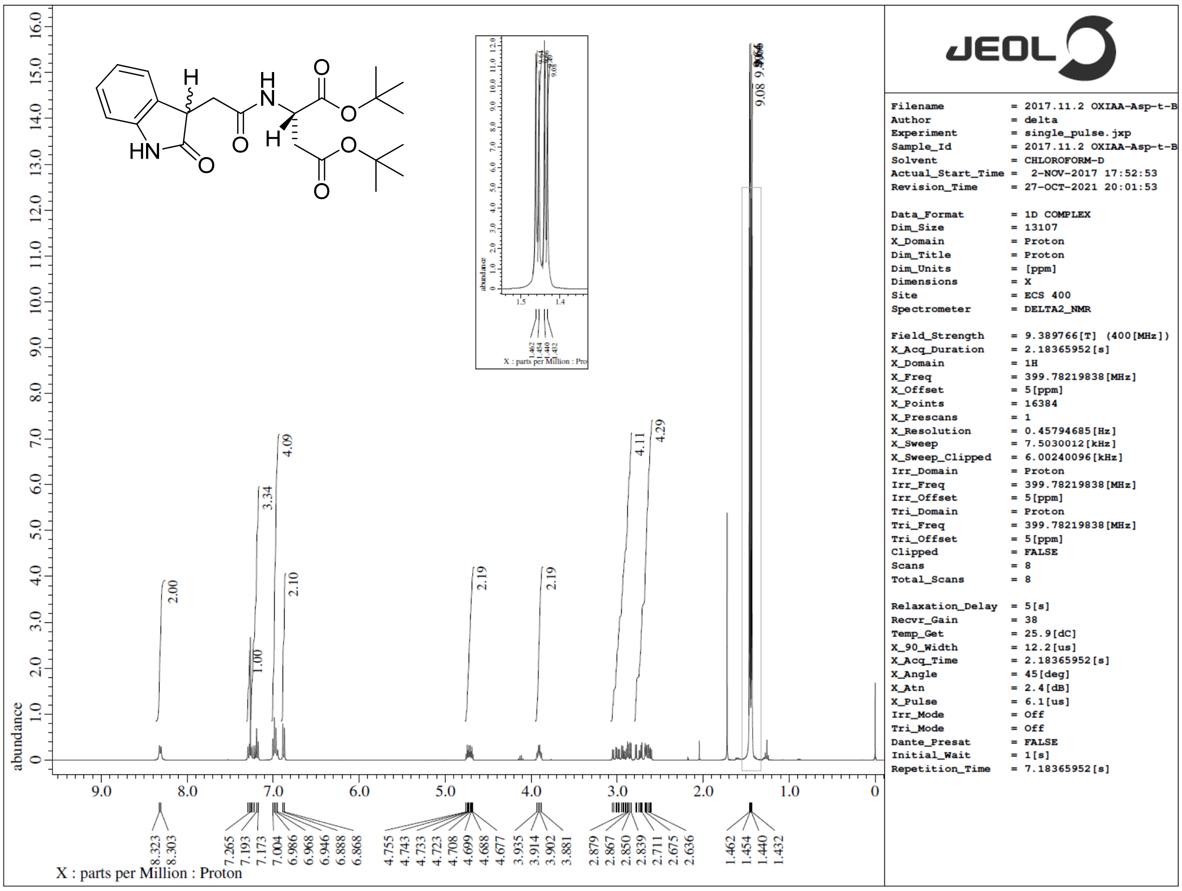

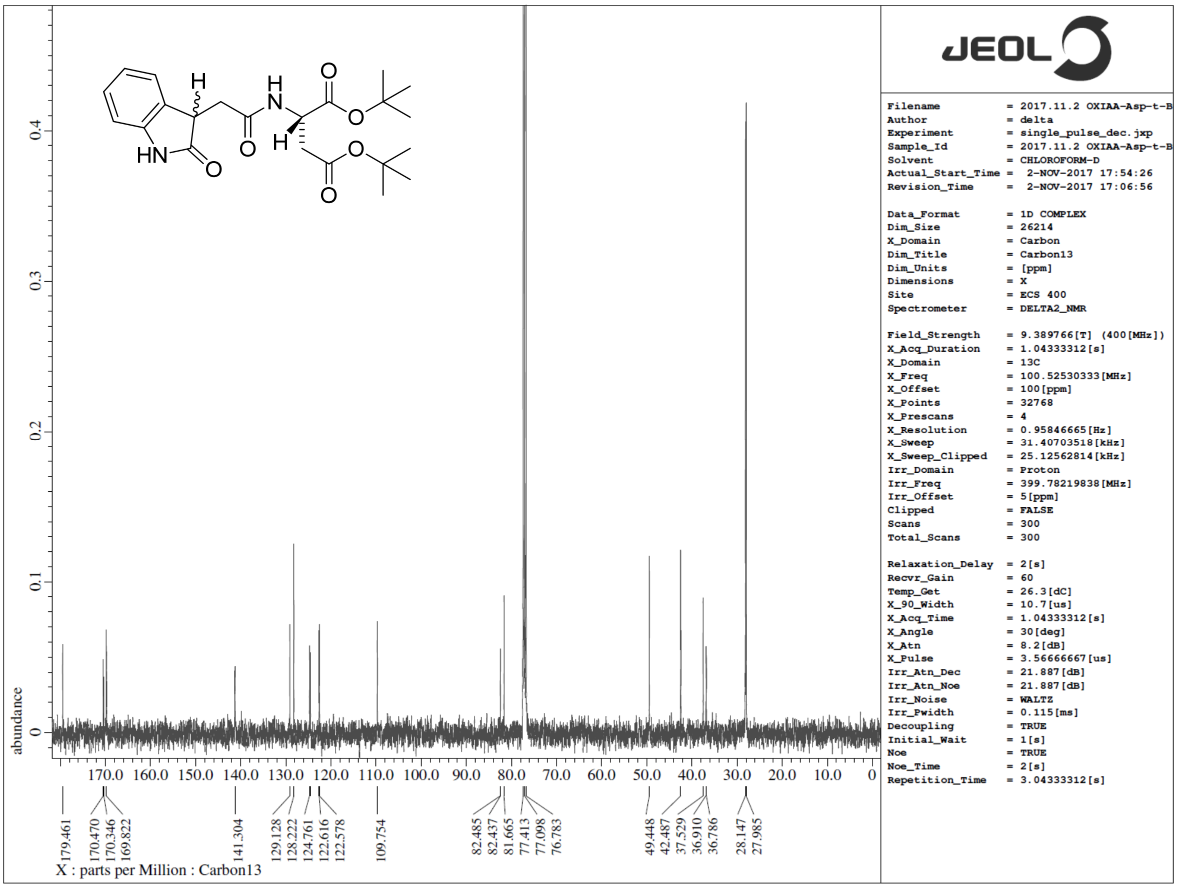
JEOL
Filename = 2017.11.2 OXIAA-Asp-t-B
Author = delta
Experiment = single_pulse_dec.jxp
Sample_Id = 2017.11.2 OXIAA-Asp-t-B
Solvent = CHLOROFORM-D
Actual_Start_Time = 2-NOV-2017 17:54:26
Revision_Time = 2-NOV-2017 17:06:56
Data_Format = 1D COMPLEX
Dim_Size = 26214
X_Domain = Carbon
Dim_Title = Carbon13
Dim_Units = [ppm]
Dimensions = X
Site = ECS 400
Spectrometer = DELTA2_NMR
Field_Strength = 9.389766[T] (400[MHz])
X_Acq_Duration = 1.04333312[s]
X_Domain = 13C
X_Freq = 100.52530333[MHz]
X_Offset = 100[ppm]
X_Points = 32768
X_Prescans = 4
X_Resolution = 0.95846665[Hz]
X_Sweep = 31.40703518[kHz]
X_Sweep_Clipped = 25.12562814[kHz]
Irr_Domain = Proton
Irr_Freq = 399.78219838[MHz]
Irr_Offset = 5[ppm]
Clipped = FALSE
Scans = 300
Total_Scans = 300
Relaxation_Delay = 2[s]
Recvr_Gain = 60
Temp_Get = 26.3[dC]
X_90_Width = 10.7[us]
X_Acq_Time = 1.04333312[s]
X_Angle = 30[deg]
X_Atn = 8.2[dB]
X_Pulse = 3.56666667[us]
Irr_Atn_Dec = 21.887[dB]
Irr_Atn_Noe = 21.887[dB]
Irr_Noise = WALTZ
Irr_Pwidth = 0.115[ms]
Decoupling = TRUE
Initial_Wait = 1[s]
Noe = TRUE
Noe_Time = 2[s]
Repetition_Time = 3.04333312[s]
abundance
0.4
0.3
0.2
0.1
0
170.0 160.0 150.0 140.0 130.0 120.0 110.0 100.0 90.0 80.0 70.0 60.0 50.0 40.0 30.0 20.0 10.0 0
179.461
170.470
170.346
169.822
141.304
129.128
128.222
124.761
122.616
122.578
109.754
82.485
82.437
81.665
77.413
77.098
76.783
49.448
42.487
37.529
36.910
36.786
28.147
27.985
X : parts per Million : Carbon13
HN
O
H
N
H
O
O

oxIAA-*L*-Asp

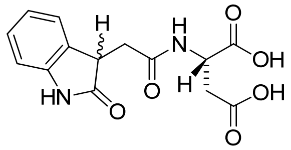

oxIAA-*L*-Asp was synthesized with same procedure for oxIAA- *L*-Ala. oxIAA-*L*-Asp was obtained as a diastereomeric mixture (10 : 9) of oxIAA-*L*-Asp at C-3 position (78 mg, 54% yield) as a pale yellow solid. ^{1}H-NMR (400 MHz, CD_3OD) δ 7.30 (d, J = 7.8 Hz, 1H), 7.23 (d, J = 7.8 Hz, 1H), 7.18 (t, J = 7.8 Hz, 2H), 6.98-6.94 (m, 2H), 6.88 (d, J = 7.8 Hz, 2H), 4.76-4.76 (m, 2H), 3.84-3.81 (m, 2H), 2.99-2.75 (m, 6H), 2.62-2.56 (m, 2H). The signal integration was adjusted to 1H from each diastereomer; ^{13}C-NMR (100 MHz, CD_3OD) δ 181.6, 174.2, 174.1, 172.7, 143.4, 130.6, 130.5, 129.2, 125.6, 125.5, 123.4, 123.3, 110.8, 110.8, 44.1, 44.0, 37.1; ESI-MS $[M+H]^+$ *m/z* 307.

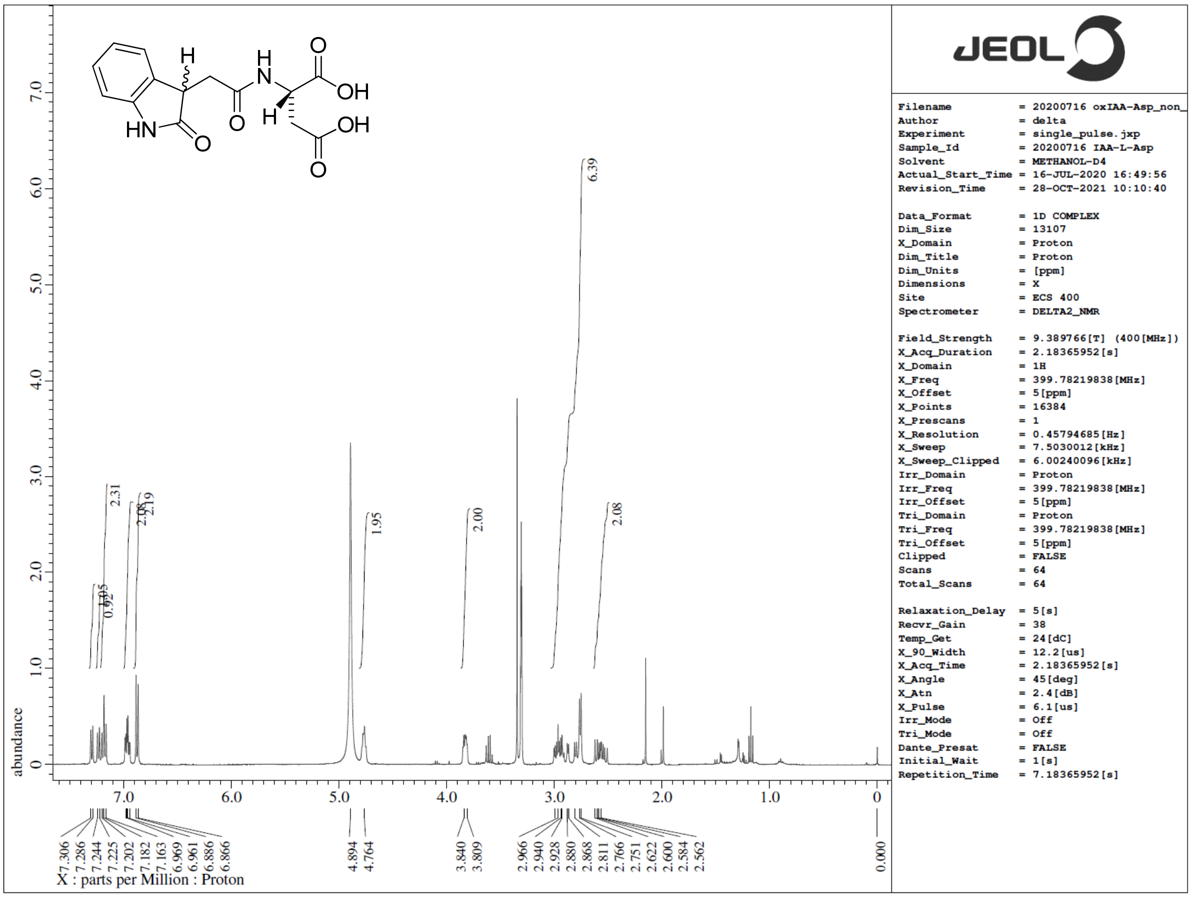

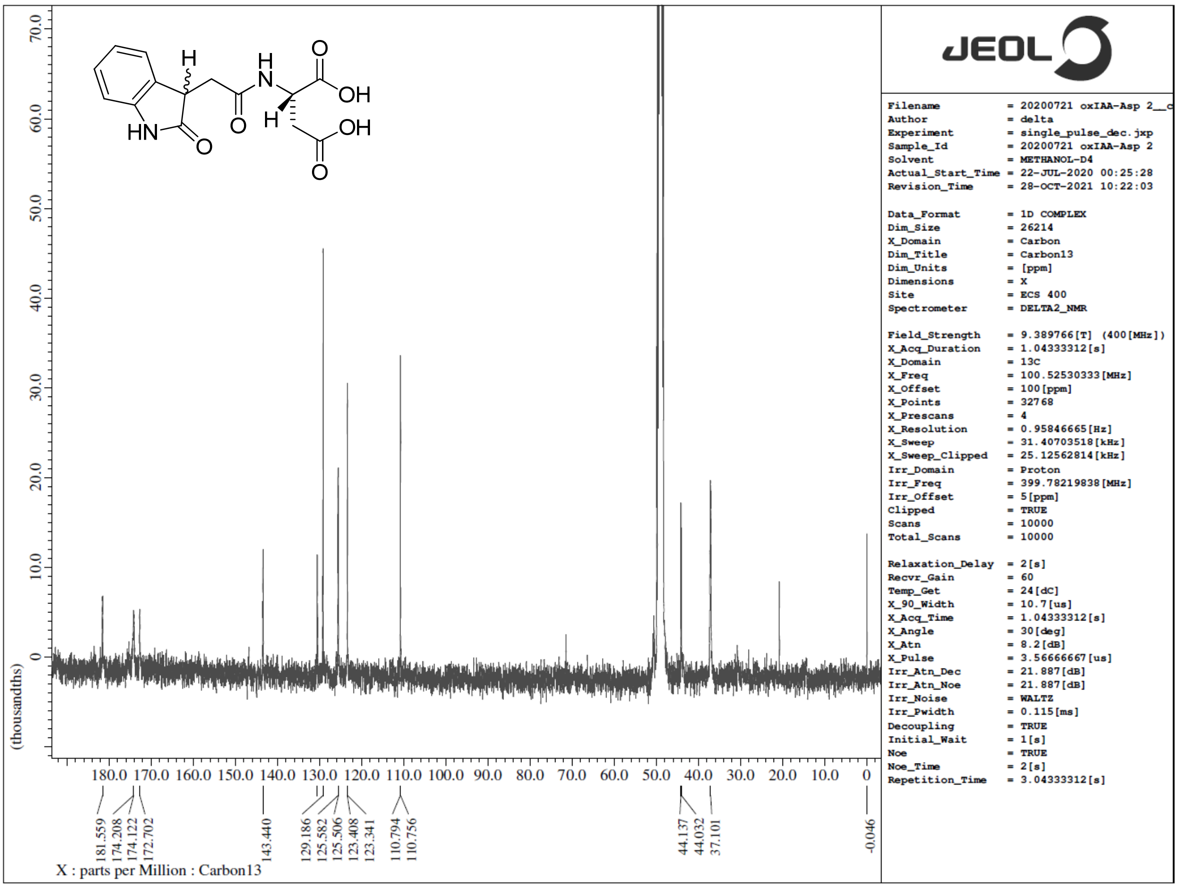

JEOL
Filename = 20200721 oxIAA-Asp 2__
Author = delta
Experiment = single_pulse_dec.jxp
Sample_Id = 20200721 oxIAA-Asp 2
Solvent = METHANOL-D4
Actual_Start_Time = 22-JUL-2020 00:25:28
Revision_Time = 28-OCT-2021 10:22:03
Data_Format = 1D COMPLEX
Dim_Size = 26214
X_Domain = Carbon
Dim_Title = Carbon13
Dim_Units = [ppm]
Dimensions = X
Site = ECS 400
Spectrometer = DELTA2_NMR
Field_Strength = 9.389766[T] (400[MHz])
X_Acq_Duration = 1.04333312[s]
X_Domain = 13C
X_Freq = 100.52530333[MHz]
X_Offset = 100[ppm]
X_Points = 32768
X_Prescans = 4
X_Resolution = 0.95846665[Hz]
X_Sweep = 31.40703518[kHz]
X_Sweep_Clipped = 25.12562814[kHz]
Irr_Domain = Proton
Irr_Freq = 399.78219838[MHz]
Irr_Offset = 5[ppm]
Clipped = TRUE
Scans = 10000
Total_Scans = 10000
Relaxation_Delay = 2[s]
Recvr_Gain = 60
Temp_Get = 24[dC]
X_90_Width = 10.7[us]
X_Acq_Time = 1.04333312[s]
X_Angle = 30[deg]
X_Atn = 8.2[dB]
X_Pulse = 3.56666667[us]
Irr_Atn_Dec = 21.887[dB]
Irr_Atn_Noe = 21.887[dB]
Irr_Noise = WALTZ
Irr_Pwidth = 0.115[ms]
Decoupling = TRUE
Initial_Wait = 1[s]
Noe = TRUE
Noe_Time = 2[s]
Repetition_Time = 3.04333312[s]
(thousandths)
181.559
174.208
174.122
172.702
143.440
129.186
125.582
125.506
123.408
123.341
110.794
110.756
44.137
44.032
37.101
-0.046
X : parts per Million : Carbon13

oxIAA-*L*-Glu *tert*-butyl diester

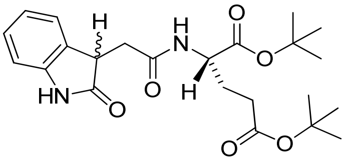

oxIAA-*L*-Glu *tert*-butyl diester was synthesized with the same procedure for oxIAA-*L*-Ala *tert*-butyl ester. oxIAA-*L*-Glu *tert*-butyl diester was obtained as a diastereomeric mixture (1 : 1) of oxIAA-*L*-Glu *tert*-butyl diester at C-3 position (373 mg, 82% yield) as a pale yellow solid. ^{1}H-NMR (400 MHz, $CDCl_3$) δ 8.53 (s, 1H), 8.49 (s, 1H), 7.28-7.27 (m, 2H), 7.19 (q, J = 7.2 Hz, 2H), 7.00 (q, J = 7.6 Hz, 2H), 6.89-6.86 (m, 4H), 4.53-4.48 (m, 2H), 3.93-3.89 (m, 2H), 3.03-2.92 (m, 2H), 2.66-2.59 (m, 2H), 2.36-2.11 (m, 6H), 1.93-1.89 (m, 2H), 1.48 (s, 9H), 1.47 (s, 9H), 1.44 (s, 18H). The signal integration was adjusted to 1H from each diastereomer; ^{13}C-NMR (100 MHz, $CDCl_3$) δ 179.7, 172.4, 172.3, 171.3, 171.3, 170.0, 169.9, 141.3, 129.1, 128.3, 124.7, 122.7, 122.6, 109.8, 82.5, 82.4, 80.9, 52.6, 52.5, 42.6, 37.0, 31.6, 31.5, 28.2, 28.1, 27.7; ESI-MS $[M+H]^+$ *m/z* 433.

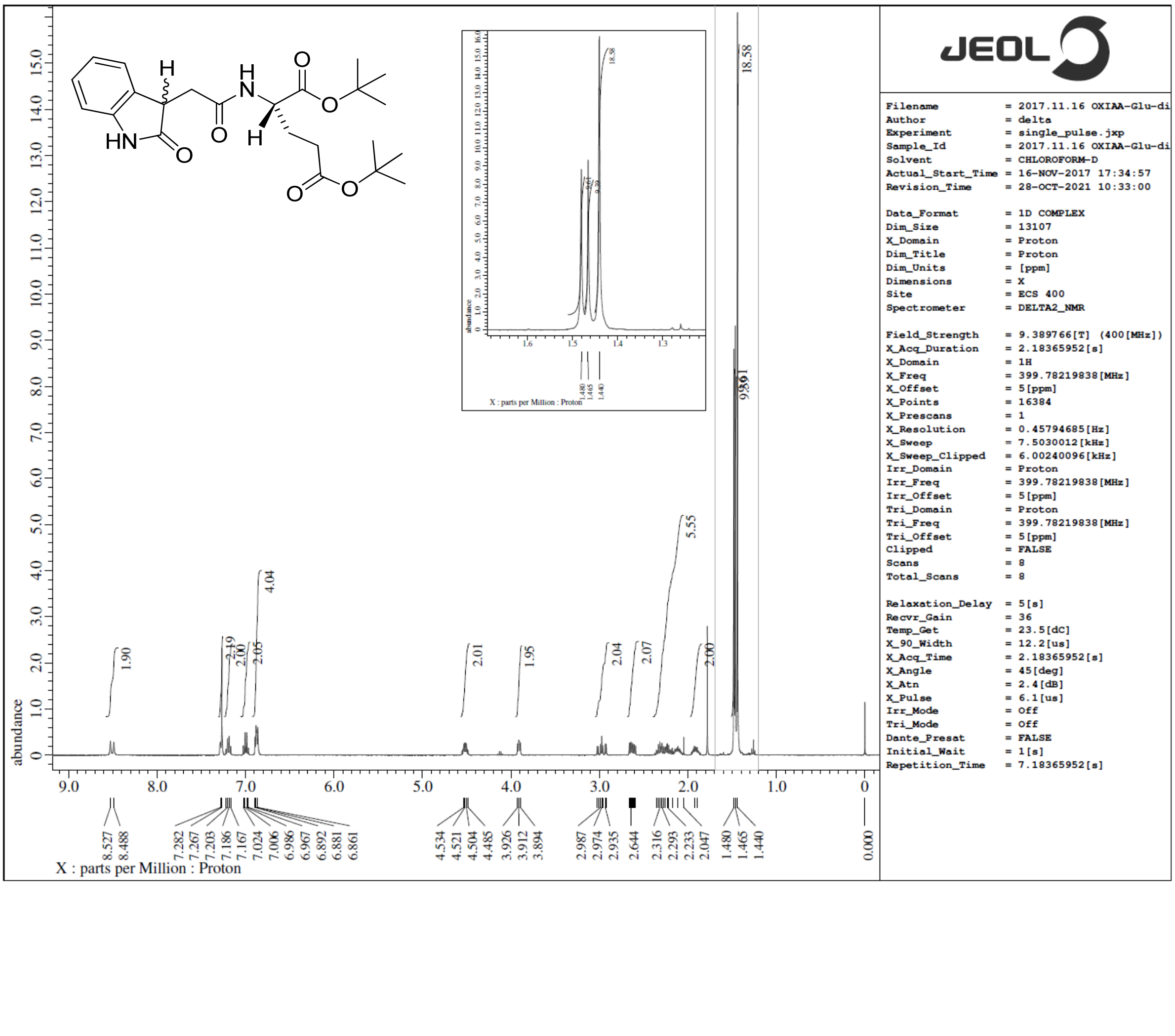

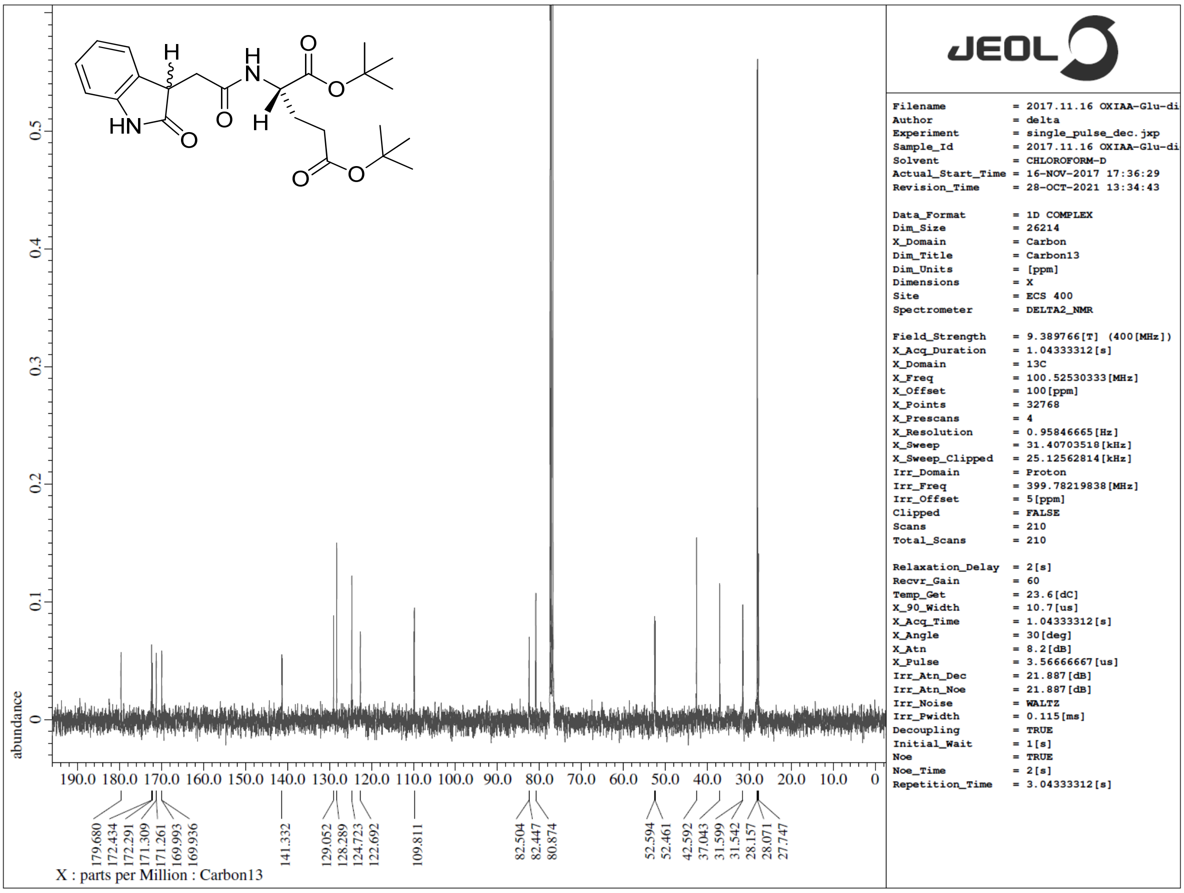

H
H
N
O
O
O
HN
O
H
O
O
abundance
0
0.1
0.2
0.3
0.4
0.5
190.0 180.0 170.0 160.0 150.0 140.0 130.0 120.0 110.0 100.0 90.0 80.0 70.0 60.0 50.0 40.0 30.0 20.0 10.0 0
179.680
172.434
172.291
171.309
171.261
169.993
169.936
141.332
129.052
128.289
124.723
122.692
109.811
82.504
82.447
80.874
52.594
52.461
42.592
37.043
31.599
31.542
28.157
28.071
27.747
X : parts per Million : Carbon13
Filename = 2017.11.16 OXIAA-Glu-di
Author = delta
Experiment = single_pulse_dec.jxp
Sample_Id = 2017.11.16 OXIAA-Glu-di
Solvent = CHLOROFORM-D
Actual_Start_Time = 16-NOV-2017 17:36:29
Revision_Time = 28-OCT-2021 13:34:43
Data_Format = 1D COMPLEX
Dim_Size = 26214
X_Domain = Carbon
Dim_Title = Carbon13
Dim_Units = [ppm]
Dimensions = X
Site = ECS 400
Spectrometer = DELTA2_NMR
Field_Strength = 9.389766[T] (400[MHz])
X_Acq_Duration = 1.04333312[s]
X_Domain = 13C
X_Freq = 100.52530333[MHz]
X_Offset = 100[ppm]
X_Points = 32768
X_Prescans = 4
X_Resolution = 0.95846665[Hz]
X_Sweep = 31.40703518[kHz]
X_Sweep_Clipped = 25.12562814[kHz]
Irr_Domain = Proton
Irr_Freq = 399.78219838[MHz]
Irr_Offset = 5[ppm]
Clipped = FALSE
Scans = 210
Total_Scans = 210
Relaxation_Delay = 2[s]
Recvr_Gain = 60
Temp_Get = 23.6[dC]
X_90_Width = 10.7[us]
X_Acq_Time = 1.04333312[s]
X_Angle = 30[deg]
X_Atn = 8.2[dB]
X_Pulse = 3.56666667[us]
Irr_Atn_Dec = 21.887[dB]
Irr_Atn_Noe = 21.887[dB]
Irr_Noise = WALTZ
Irr_Pwidth = 0.115[ms]
Decoupling = TRUE
Initial_Wait = 1[s]
Noe = TRUE
Noe_Time = 2[s]
Repetition_Time = 3.04333312[s]

oxIAA-*L*-Glu

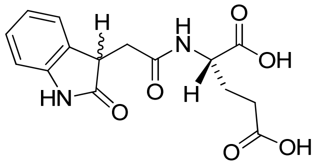

oxIAA-*L*-Glu was synthesized with the same procedure for oxIAA-*L*-Ala. oxIAA-*L*-Glu was obtained as a diastereomeric mixture (1 : 1) of oxIAA-*L*-Glu at C-3 position (78 mg, 54% yield) as a pale yellow solid. ^{1}H-NMR (400 MHz, CD_3OD) δ 7.33-7.28 (m, 1H), 7.22-7.16 (m, 3H), 6.99-6.95 (m, 2H), 6.88 (q, J = 4.0 Hz, 2H), 4.41-4.38 (m, 1H), 4.34-4.30 (m, 1H), 3.86-3.79 (m, 1H), 2.95 (q, J = 7.3 Hz, 2H), 2.71-2.56 (m, 2H), 2.38-2.26 (m, 4H), 2.22-2.06 (m, 2H), 1.94-1.82 (m, 2H). The signal integration was adjusted to 1H from each diastereomer; ^{13}C-NMR (100 MHz, CD_3OD) δ 180.4, 180.3, 174.6, 171.4, 171.3, 142.2, 142.1, 129.3, 129.1, 127.9, 124.1, 124.1, 122.1, 122.0, 109.6, 109.5, 53.5, 36.1, 35.8, 30.7, 30.7, 27.4, 27.4; ESI-MS $[M+H]^+$ *m/z* 321.

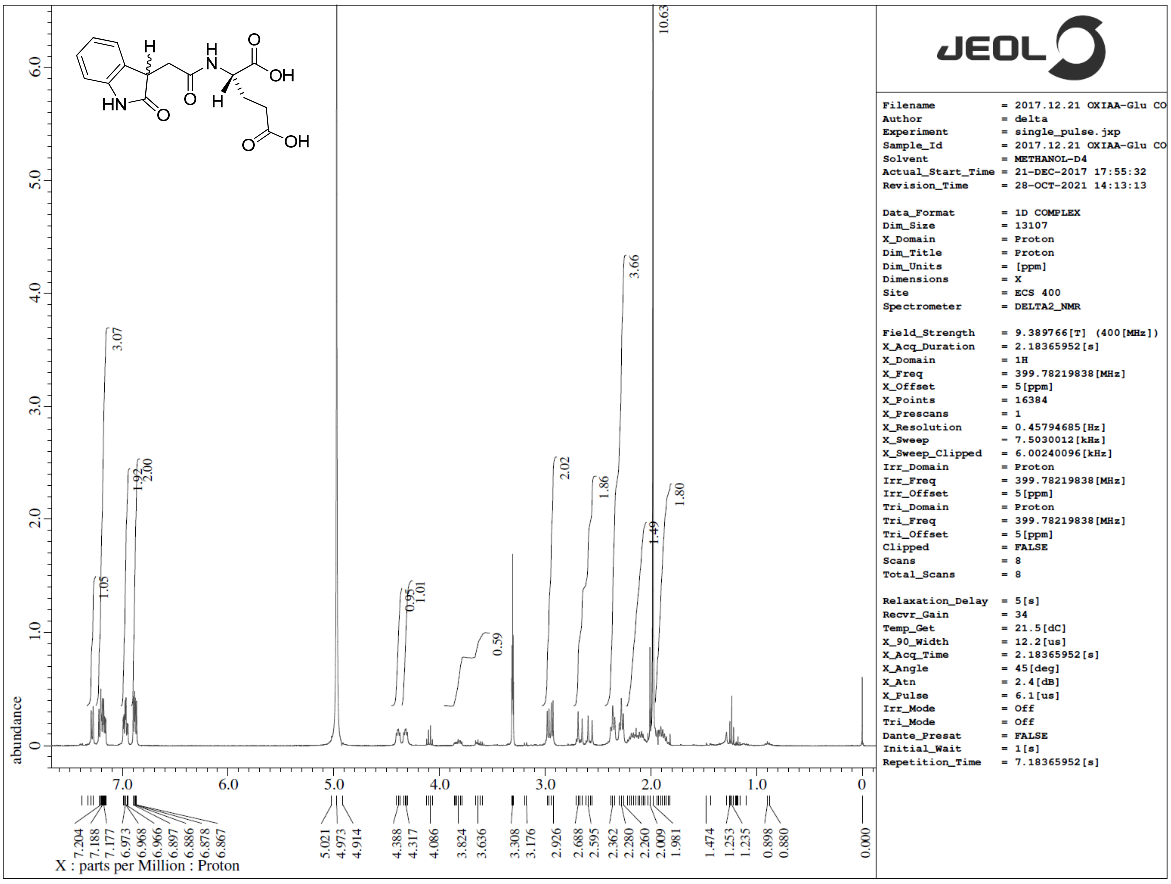

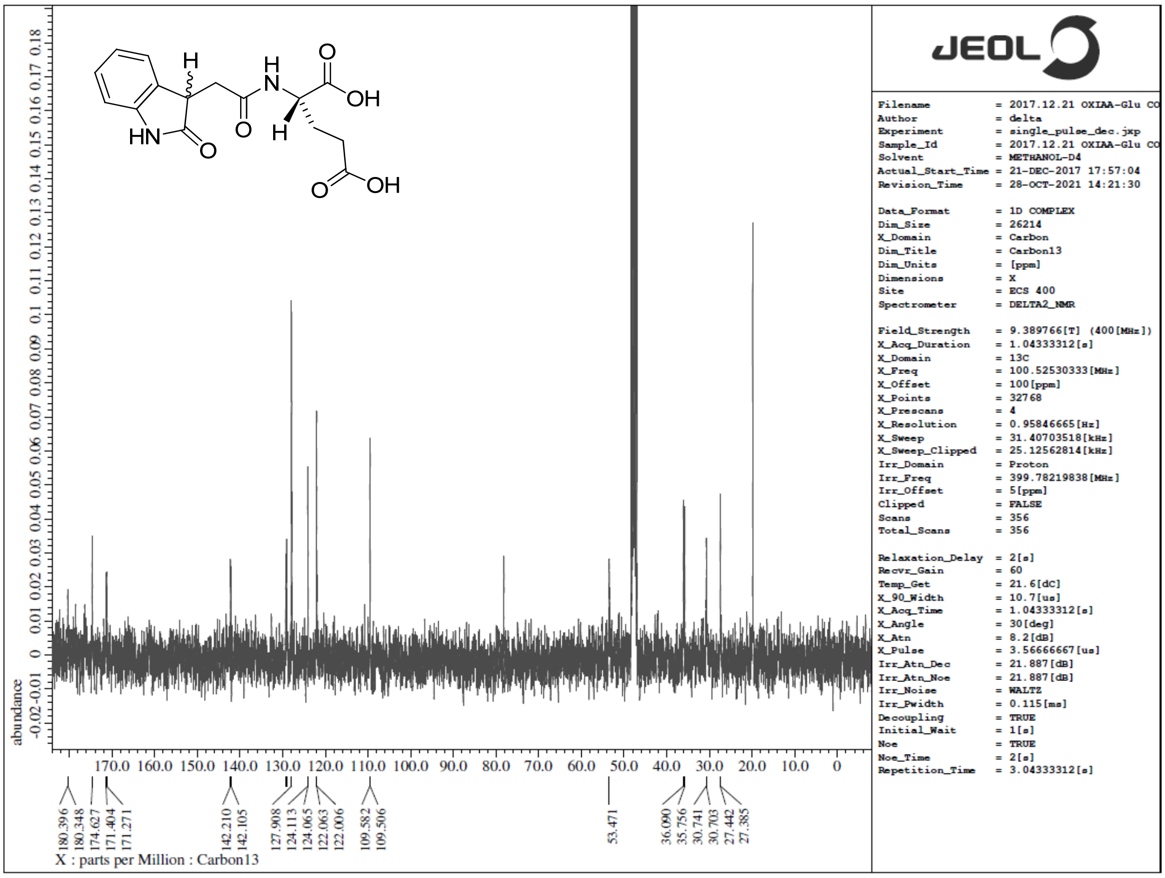

Derivatives of IAA-Asp diester and IAA-Glu diester

IAA-Asp 4-methylester, IAA-Asp 1-methyl ester, IAAL-Asp diethyl ester (IAA-Asp-DE) , and IAA-*L*-Asp dibutyl ester (IAA-Asp-DB),

IAA-Asp-DE and IAA-DB were synthesized according to the same methods as IAA-Asp-DM. Briefly, IAA was condensed with *L*-aspartic acid diethyl ester hydrochloride or *L*-asparatic acid dibutyl ester. *L*-asparatic acid dibutyl ester was synthesized from L-asparatic acid according to previous method [14]. IAA-Asp 4-methylester and 1-methyl ester were synthesized by coupling of IAA-benzotriazol with monomethyl aspartic acid according to the previous methods [15].

IAA-Asp 4-methyl ester was obtained as a pale yellow solid (34 mg, 28%); ^{1}H-NMR (400 MHz, acetone-D_6) δ 10.14 (s, 1H), 7.57 (d, J = 7.8 Hz, 1H), 7.39-7.36 (m, 2H), 7.28 (s, 1H), 7.10 (t, J = 7.6 Hz, 1H), 7.01 (t, J = 7.6 Hz, 1H), 4.82 (dd, J = 13.5, 5.7 Hz, 1H), 3.72 (d, J = 1.8 Hz, 2H), 3.50 (s, 3H), 2.84 (d, J = 6.0 Hz, 2H); ^{13}C-NMR (100 MHz, acetone-D_6) δ 172.4, 172.1, 171.6, 137.6, 128.4, 124.9, 122.3, 119.7, 119.5, 112.2, 109.4, 51.9, 49.7, 36.6, 33.6; MALDI-TOFMS $[M+Na]^+$ *m/z* 327.092.

IAA-Asp 1-methyl ester obtained as a pale yellow solid (38 mg, 24%); ^{1}H-NMR (400 MHz, acetone-D_6) δ 10.11 (s, 1H), 7.59 (d, J = 7.8 Hz, 1H), 7.40 (dd, J = 17.6, 8.0 Hz, 2H), 7.27 (d, J = 2.3 Hz, 1H), 7.10 (t, J = 7.6 Hz, 1H), 7.02 (t, J = 8.0 Hz, 1H), 4.86-4.82 (m, 1H), 3.71 (s, 2H), 3.60 (s, 3H), 2.83 (dd, J = 5.5, 1.4 Hz, 2H); ^{13}C-NMR (100 MHz, acetone-D_6) δ 172.4, 172.1, 171.9, 137.6, 129.1, 128.4, 124.8, 122.3, 119.6, 119.5, 112.1, 109.5, 52.5, 49.7, 36.6, 33.5; MALDI-TOFMS $[M+Na]^+$ *m/z* 327.102.

***L*-Asparatic acid dibutyl ester** ; ^{1}H-NMR (400 MHz, $CDCl_3$) δ 4.58 (t, J = 5.3 Hz, 1H), 4.26-4.11 (m, 4H), 3.27 (ddd, J = 46.7, 17.9, 5.0 Hz, 2H), 1.62 (td, J = 14.5, 7.3 Hz, 4H), 1.41-1.32 (m, 4H), 0.92 (td, J

= 7.3, 2.3 Hz, 6H); ^{13}C-NMR (100 MHz, $CDCl_3$) δ 170.2, 168.2, 66.8, 65.6, 49.7, 34.0, 30.5, 30.3, 19.1, 19.0, 13.7, 13.7; MALDI-TOFMS $[M+H]^+$ *m/z* 246.175.

IAA-Asp-DB was obtained as a yellow solid (80 mg, 81%); ^{1}H-NMR (400 MHz, $CDCl_3$) δ 8.25 (s, 1H), 7.55-7.51 (m, 1H), 7.41-7.32 (m, 1H), 7.21-7.08 (m, 2H), 6.62 (dd, J = 8.0 Hz, 1H), 4.87-4.80 (m, 1H), 4.18-3.97 (m, 2H), 3.91-3.71 (m, 4H), 3.03-2.62 (m, 2H), 1.93-1.60 (m, 4H), 1.52-1.20 (m, 4H), 0.86 (t, J = 7.3Hz, 6H); ^{13}C-NMR (100 MHz, $CDCl_3$) δ 171.2, 170.7, 170.7, 136.4, 127.1, 123.7, 122.5, 120.0, 118.7, 111.4, 108.7, 65.7, 64.9, 48.6, 36.4, 34.0, 33.4, 32.6, 30.4, 19.0, 13.7; MALDI-TOFMS $[M+H]^+$ *m/z* 425.227.

IAA-Asp-DE was obtained as a colorless oil (150 mg, 85%); ^{1}H-NMR (400 MHz, $CDCl_3$) δ 8.78 (s, 1H), 8.02 (s, 1H), 7.53 (d, J = 7.8 Hz, 1H), 7.34 (d, J = 8.2 Hz, 1H), 7.18 (t, J = 7.6 Hz, 1H), 7.12-7.08 (m, 1H), 6.70 (d, J = 8.2 Hz, 1H), 4.87-4.82 (m, 1H), 4.11 (qd, J = 7.1, 3.0 Hz, 2H), 3.89 (qd, J = 7.2, 1.9 Hz, 2H), 2.91-2.74 (m, 2H), 1.16 (t, J = 7.1 Hz, 3H), 1.06 (t, J = 7.1 Hz, 3H); ^{13}C-NMR (100 MHz, $CDCl_3$) δ 171.4, 170.6, 170.6, 162.6, 136.5, 127.0, 123.9, 122.3, 119.7, 118.5, 111.5, 108.2, 61.8, 60.9, 48.6, 36.4, 33.4, 13.9, 13.9; MALDI-TOFMS $[M+H]^+$ *m/z* 347.181.

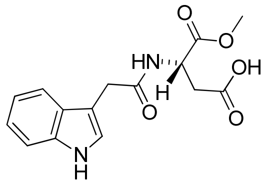

IAA-Asp 1-methyl ester

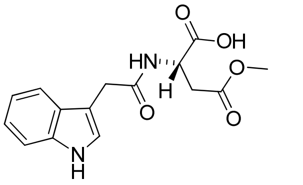

IAA-Asp 4-methyl ester

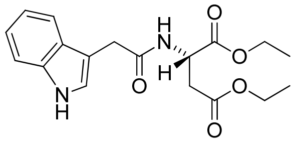

IAA-Asp diethyl ester

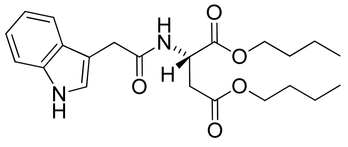

IAA-Asp dibutyl ester

Supplementary table 1. Nucleotide sequences of oligo DNAs

genotyping primers

Gene	Primer name	Sequence
iamt1 c-2_CRISPR	*iamt1-LP*	AGCTTCTTCCACCACTTGTCTC
	iamt1-RP	GCACTAGCACACTCAACACCTC
ill2_SALK_087005C	ILL2-LP	AGGCCAATACAAATGTCTCTTAGGT
	ILL2-RP	ATTCAATGGAGTGTTAGGTTTGTGC
ill1_SALK_046056C	ILL1-LP	AAATCCAAATCTTTTGACCACG
	ILL1-RP	GCTAATTCGAGGAAATTGATCG
dao2-1_SALK_205223C	AtDAO2-LP	GCCTTGTTTCCATTCTCCTATG
	AtDAO2-RP	TCCCAATAACAGTTTTGGATCC
ill6_SALK_024894C	ILL6-LP	GAATGGGAACATATAAGCAAAGTTG
	ILL6-RP	CGTTTCTTGAGGTAGTAGAAGCTTG
ill3_SALK_092410C	ILL-3-LP	CTCGACTTCTCTCTCTCTCTCTTTG
	ILL3-RP	GTTTGTTGTCCCATGAAAGATTTAC
iar3_SALK_022636C	IAR3-LP_A	CACAAGGCAAGCGAGCGTGAA
	IAR3-RP_A	AGCAGACGCAACGGTAAGAGAGC
	IAR3-LP_B	GCGAGTGCTTACAGGTGGTT
	IAR3-RP_B	TTTACGGGAAATGTTTGTACGAC
ugt84b1-c2 _CRISPR	UGT84B1-LP-A	GTTGATCATTGTAACGGTCAAGTGT
	UGT84B1-RP-A	ACCAAAACCCATTTCACATACCTCA
	UGT84B1-LP-B	CAATCAAATAAAAAGGAACAAATGG
	UGT84B1-RP-B	TCCGCCATTAGATTATAGAAGTGAG
dao1-1_SALK_093162	AtDAO1-LP	TTCCCCACGGAATTAAGGTAC
	AtDAO1-RP	CTATGGGGAAAAAGGTTCCTG
dao1 dao2 _CRISPR	DAO1CRP5-GT1	AATCGAAATTTGGCGCGTGG
	DAO2CRP6-GT2	GTCCTCCGTCACTGATAGGC
	DAO1-KO-LP	CGATAAAAATCTAGAGGCAAGATTCC
	DAO1-KO-RP	CTTCCGTTGCTCCATATCGTAGC
ilr1_ SAIL_631_F01	ILR1-LP	CACTCGCTCGCGGGATGCTT
	ILR1-RP	TCCGCTGATTTCAGCCAAGATTCTG

Primers for vector construction

Constructs and amplified fragments	Primer name	sequence
genome fragment (3934 bp) of *ILR1* from genomic DNA	ILR1-genome-promoer-5'	GAGTCGGGTTTGGACTTGAGACTATTGG
	ILR1 genome-UTR-3'	TTCCCAACCCGAAACCTAACCTCAC

for PCR template		
pDONR-proILR1::genome ILR1	ILR1-pro-5' attB1	AAAAAGCAGGCTTCGGACTCCTTATTCTGAATATGG
	ILR1-3'-attB2	AGAAAGCTGGGTGTAATTCACTCTTAACCTCTTCT
pDONR-genome ILR1	ILR1-gORF-5'-attB1	AAAAAGCAGGCTTCATGGATTTCTCAGGGAGCTTCT
	ILR1-3'-attB2	AGAAAGCTGGGTGTAATTCACTCTTAACCTCTTCT
genome fragment (4992 bp) of *IAR3* from genomic DNA PCR template	IAR3-genome-promoer-5'	CGATTCTCAACAGATTGGCAGACAATAGTGAA
	IAR3 genome-UTR-3'	TCTTTACGGGAAATGTTTGTACGACAACAAC
pDONR-proIAR3::genome IAR3	IAR3-pro-5' attB1	AAAAAGCAGGCTTC GGCTCTAGACTCTCTCTGCTCTTTCTGTTAAGC
	IAR3-3'-attB2	AGAAAGCTGGGTGAAGTTCATCTTTTTTGTTACTCTTATTTAG
pDONR-genome IAR3	IAR3-gORF-5'-attB1	AAAAAGCAGGCTTCATGAGTTTCTTCAAATGGGTTTC
	IAR3-3'-attB2	AGAAAGCTGGGTGAAGTTCATCTTTTTTGTTACTCTTATT
genome fragment (3830 bp) of *ILL2* from genomic DNA PCR template	ILL2-genome-promoer-5'	GGTCTATGGTTCAGAAATTTGGATTAAGGATTAG
	ILL2 genome-UTR-3'	CTGAACCAATAACCGATGAACAACCTGTC
pDONR-proILL2::genome ILL2	ILL2-pro-5' attB1	AAAAAGCAGGCTTCCTCTCCTGGTCTATGCTTAATTTG
	ILL2-3'-attB2	AGAAAGCTGGGTGCAACCTGTCTACAACCAAGGTGTAGA
pDONR-genome ILL2	ILL2-gORF-5'-attB1	AAAAAGCAGGCTTCATGGCTCTAAACAAGCTCCTCAGTTTG
	ILL2-3'-attB2	AGAAAGCTGGGTGCAACCTGTCTACAACCAAGGTGTAGA
pDONR-AtDAO1_cDNA_ORF	AtDAO1_attB1	AAAAAGCAGGCTTCATGGGGGAACTAAACGGAG
	AtDAO1-attB2	AGAAAGCTGGGTGTCATTTATCTAGTCCTGCATG
pDONR-AtDAO2_cDNA_ORF	AtDAO2-attB1	AAAAAGCAGGCTTCATGGCGGAAGTAAATGGAGTC
	AtDAO2-attB2	AGAAAGCTGGGTGTTAATCTATCTTTGGGACGTC
pDONR-OsDAO_ORF	OsDAO-attB1	AAAAAGCAGGCTTCATGGTCGAGATTCCTGCG
	OsDAO-attB2	AGAAAGCTGGGTGTTAGGCAGCTAAACGCGCAAG
pDONR-BdDAO_ORF	BdDAO-attB1	AAAAAGCAGGCTTCATGGTCGAAATTCCCGTCATTGA
	BdDAO-attB2	AGAAAGCTGGGTGTGCCGCCATTCGCGCCAATGC
pDONR-AtGH3-17_cDNA_ORF	GH3-17-attB1	AAAAAGCAGGCTTC ATGATACCAAGTTACGACCCAAA
	GH3-17-attB2	AGAAAGCTGGGTGTTAAGAATCTAAACCAAGTGGTTCC
attB adapter primers	Adapter primer for attB1	GGGGACAAGTTTGTACAAAAAAGCAGGCT
	Adapter primer for attB2	GGGGACCACTTTGTACAAGAAAGCTGGGT
The expression vector for recombinant Enzymes		
pCold I_OsDAO	pCold_OsDAOs _Kpn-5’	GGCATATGGAGCTCGGTACCATGGTGGAAATCCCCGCC
	pCold_OsDAOs _HindIII-3’	GCAGGTCGACAAGCTTTTACGCGGCAAGACGTGC
pCold_DAO1	pCold_DAO1_BamHI-5'	TACCCTCGAGGGATCATACATGGGGGAACTAAACG

	pCold_DAO1_Hind-3'	GCAGGTCGACAAGCTTCATTTATCTAGTCCTGCATG
pEU1-GST_ILR1 for wheat cell-free protein expression	pEU1-ILR1_BamHI-5’	GATATCTCGAGGATCCTGGGAGCTACGATTCTGG
	pEU1-ILR1_Kpn I-3’	ACTAGTGCGGCCGCGGTACCCTATAATTCACTCTTAACCTC

Red colored sequence shows the attB1 and attB2 adapter sequence for adaptor PCR (2 rounds of PCR with shorter oligos) using attB adapter primers.

Supplementary table 2 Parameters for LC-ESI-MS/MS analysis.

For analysis of oxIAA-amino acid conjugates

HPLC gradient program	Time (min)	flow(ml/min)	Solvent Ratio B(%)	mobile phase		column temp(℃)
				A	B	
	0	0.2	3	water containing 0.1% formic acid	MeCN,0.1% formic acid	30
	2	0.2	8			
	9	0.2	11			
	15	0.2	15			
	15.1	0.2	98			
	20	0.2	98			
	20.1	0.2	3			
	25	0.2	3			

	Retention time (min)
oxIAA-Asp<br>D_2-oxIAA-Asp	5.9, 6.5
oxIAA-Glu<br>D_2-oxIAA-Glu	6.9, 7.4

	ESI	transitions for quantifications (m/z)	Collision energy (V)	Fragmentor (V)	Cell Accelerator voltage (V)	Dwell time (msec)	Capillary (V)	source gas (℃)	gas flow (l/min)	Nebulizer (psi)
oxIAA-Asp<br>D_2-oxIAA-Asp	+	307.08/147.1<br>309.08/149.1	30	104	7	125	4000	300	10	30
oxIAA-Glu<br>D_2-oxIAA-Glu	+	321.09/147.1<br>323.09/149.1	24	104	7	125	4000	300	10	30

For analysis of IAA-amino acid conjugates

HPLC gradient program	Time (min)	flow(ml/min)	Solvent Ratio B(%)	mobile phase		column temp(℃)
				A	B	
	0	0.2	3	water containing 0.1% formic acid	MeCN,0.1% formic acid	30
	2	0.2	8			
	9	0.2	11			
	15	0.2	15			
	15.1	0.2	98			
	20	0.2	98			
	20.1	0.2	3			
	25	0.2	3			

	Retention time (min)
IAA-Ala<br>D_2-IAA-Ala	16.7
IAA-Leu<br>D_2-IAA-Leu	18.4

	ESI	transitions for quantifications (m/z)	Collision energy (V)	Fragmentor (V)	Cell Accelerator voltage (V)	Dwell time (msec)	Capillary (V)	source gas (℃)	gas flow (l/min)	Nebulizer (psi)
IAA-Ala<br>D_2-IAA-Ala	+	247.1/130.1<br>249.1/132.1	22	85	7	250	4000	300	10	30
IAA-Leu<br>D_2-IAA-Leu	+	289.16/130.1<br>291.16/132.1	26	104	7	250	4000	300	10	30

For analysis of DioxIAA

HPLC gradient program for DioxIAA, IAA and oxIAA	Time (min)	flow(ml/min)	Solvent Ratio B(%)	mobile phase		column temp(℃)
				A	B	
	0	0.2	3	water containing 0.01% acetic acid	MeCN,0.05% acetic acid	40
	3	0.2	3			
	5	0.2	15			
	15	0.2	15			
	16.1	0.2	98			
	19	0.2	98			
	19.1	0.3	98			
	21	0.3	98			
	21.1	0.2	3			
	26	0.2	3			

	Retention time (min)
DioxIAA<br>D_2-DioxIAA	2.9

	ESI	transitions for quantifications (m/z)	Collision energy (V)	Fragmentor (V)	Cell Accelerator voltage (V)	Dwell time (msec)	Capillary (V)	source gas (℃)	gas flow (l/min)	Nebulizer (psi)
DioxIAA<br>D_2-DioxIAA	+	208.05/146.0<br>210.06/148.0	6	52	7	250	4000	300	9	30

Supplementary text

Synthetic DNA sequence of OsDAO and Bd DAO.

OsDAO [LOC4336150, Os04g0475600] codon optimized for *Escherichia coli* expression

ATGGTGGAAATCCCCGCCATTGATCTGCGCTTAGCTGGAGGTGGTGGCGGTGCAGAGGAAACCGCTCGTCTGCGTG
ACGCATGTGCACGTCTGGGTTGCTTCCGCGTTTCAGGCCATGGTGTGCCACCGGGGTTGCAAGCTGAGATGAAAGC
TGCGGTTCGTGCCCTGTTTGATCTGCCGGATGATGCTAAACGGCGCAATGCGGACATCATCCCGGGTAGTGGCTAT
GTACCGCCAGGGACAGCGAATCCCCTGTATGAGGCGTTTGGTCTGTGTGATGCGGCAGCTCCGGCCGATGTAGACG
CCTTTTGTGCCCGGCTCGACGCGCCACCTCATGTGCGTGAAACGGTCAAAGCGTATGCGGAACGCATGCACTCTCT
GATTGTGGACGTCGCCGGGAAAGTTGCCGCGAGTCTGGGTTTGCATGGAGCGTCCTTTCAGGATTGGCCCTGCCAG
TTCCGTATGAACCGCTATAACTACACCCAGGATTCGGTCGGCTCTCCGGGCGTTCAGGTGCACACCGATAGCGGAT
TTCTCACCGTTCTGCAGGAGGATGAATGCGTGGGCGGGCTTGAAGTGCTGGACCCTGCAGCCGGCGAATTTGTGCC
GGTAGATCCGCTTCCGGGCAGCTTCGTCGTAAACGTAGGGGATGTTGGCCAAGCCTGGTCCAATGGCCGCCTCCAC
AACGTGAAGCATCGCGTGCAATGCGTTGCAGCAGTGCCACGCGTTAGCATTGCCATGTTCCTGCTGGCGCCGAAAG
ACGACACTGTCTCGGCGCCGGGTGAACTGGTCGATGGCGAACATCCTCGTCGTTACCGCGAGTTCAAGTACGACG
ATTATCGCCGCTTACGCTTGAGCACGGGTGAACGCGCGGGCGAAGCATTAGCACGTCTTGCCGCGTAA

OsDAO [LOC4336150, Os04g0475600] codon optimized for *Arabidopsis thaliana* expression

ATGGTCGAGATTCCTGCGATCGACCTTAGATTGGCAGGAGGTGGAGGAGGAGCAGAAGAAACCGCTAGACTCAGA
GATGCTTGCGCCAGATTAGGGTGCTTTAGGGTTTCAGGGCATGGAGTCCCTCCAGGACTTCAAGCTGAGATGAAGG
CTGCAGTCAGAGCCCTGTTCGATTTGCCAGATGATGCGAAAAGACGGAATGCAGACATCATTCCGGGTTCTGGATA
CGTTCCTCCTGGTACTGCTAATCCGCTCTATGAGGCGTTTGGGCTGTGTGATGCAGCCGCTCCAGCTGACGTTGATG
CCTTTTGCGCGAGATTGGATGCACCACCGCATGTGCGAGAAACTGTGAAGGCATATGCTGAACGTATGCATTCGTT
GATAGTGGACGTTGCTGGAAAAGTCGCTGCTAGTTTAGGACTTCATGGCGCTAGTTTCCAGGATTGGCCATGTCAG
TTCAGGATGAATCGTTACAACTACACGCAGGATTCAGTTGGTTCTCCTGGTGTCCAAGTGCATACAGATAGCGGTT
TTCTAACTGTGTTGCAAGAGGACGAGTGTGTTGGTGGATTGGAGGTTCTAGATCCAGCAGCTGGAGAGTTTGTGCC
TGTAGATCCGCTTCCCGGTAGCTTCGTGGTTAACGTAGGCGATGTTGGGCAAGCTTGGTCCAATGGTCGACTACAC
AACGTCAAGCACAGGGTTCAGTGTGTTGCAGCGGTACCCAGGGTTTCCATTGCTATGTTTCTTCTCGCCCCTAAAG
ACGATACCGTATCTGCTCCAGGTGAACTTGTGGATGGCGAACACCCTAGAAGATATCGGGAGTTCAAGTACGACG
ATTATAGGCGACTGAGACTCTCTACAGGCGAACGTGCAGGGGAAGCACTTGCGCGTTTAGCTGCCTGA

2-oxoglutarate-dependent dioxygenase BdDAO [*Brachypodium distachyon* XP_003579963.1] codon optimized for *Arabidopsis thaliana* expression.

ATGGTCGAAATTCCCGTCATTGATCTCCGATTAGCTGGTGCTGCCCCTGAAGAATCTGCTAGGCTGAGAGATGCAT
GTGAAAGACTCGGTTGCTTCCGAGTATTTGGGCATGGTGTTCCTGCGGCACTTCAAGCTGACATGAAAGCAGCTCT
AAGAGCGCTATTCGATCTGCCAGATGATGCAAAGAGGCGTAATACCGAGATCATAGCTGGATCTGGTTATGTGCC
ACCAAGTGCTGCCAATCCCTTGTATGAGGCCTTCGGATTGTGGGATGCTGCAGTTCCTGCAGATGTTGACGCGTTTT
GCGCTCGTCTAGATGCCCCTCCACACGCTAGAGAAGCTGTGAAGAGCTATGCTGAGAAGATGCATGAGTTGATCGT
GGATGTAGCTGGAAAAGTTGCCGCTTCTCTTGGCCTTGAAGGTCATCCTTTCCAAGACTGGCCTTGCCAATTTCGGA
TGAATCGTTACAACTACACGGAGGACACTGTTGGAAGCTCAGGAGTTCAGATCCATACAGACTCAGGCTTCCTCAC
CGTTTTACAGGAGGATGACTGTGTTGGTGGCTTGGAAGTGCTGGACCCTGCAGCTGGAGAATTCGTGCCGGTTGAT
CCGTTTCCAGGGTCTTTTCTCGTCAACATTGGAGATGTTGGTACTGCATGGTCGAATGGGAGATTACACTCCGTAA
AACACAGAGTCCAGTGTGTCGCCGCTGTACCAAGGATTTCCATAGCCATGTTTCTTCTTGCACCGAAGGATGATAG
AGTGTGTACACCTGAGGCTTTTGTGGATGCTGATCATCCGCGTAGGTATAGAGCGTTCAACTACGACGAGTACAGA
AAACTTCGGTTGAGTACTGGAGAGAGAGCTGGTGAAGCATTGGCGCGAATGGCGGCA

References and Notes

1. Curtis, M.D. & Grossniklaus, U. A gateway cloning vector set for high-throughput functional analysis of genes in planta. *Plant Physiol* **133**, 462-469 (2003).
2. Nakagawa, T. *et al.* Development of series of gateway binary vectors, pGWBs, for realizing efficient construction of fusion genes for plant transformation. *J Biosci Bioeng* **104**, 34-41 (2007).
3. Narusaka, M., Shiraishi, T., Iwabuchi, M. & Narusaka, Y. The floral inoculating protocol : a simplified Arabidopsis thaliana transformation method modified from floral dipping. *Plant biotechnology* **27**, 349-351 (2010).
4. Trott, O. & Olson, A.J. AutoDock Vina: improving the speed and accuracy of docking with a new scoring function, efficient optimization, and multithreading. *J Comput Chem* **31**, 455-461 (2010).
5. Guenin, E., Monteil, M., Bouchemal, N., Prange, T. & Lecouvey, M. Syntheses of phosphonic esters of alendronate, pamidronate and neridronate. *European Journal of Organic Chemistry* **2007**, 3380-3391 (2007).
6. Chen, Q., Westfall, C.S., Hicks, L.M., Wang, S. & Jez, J.M. Kinetic basis for the conjugation of auxin by a GH3 family indole-acetic acid-amido synthetase. *J Biol Chem* **285**, 29780-29786 (2010).
7. Morrison, J.F. Kinetics of the reversible inhibition of enzyme-catalysed reactions by tight-binding inhibitors. *Biochim Biophys Acta* **185**, 269-286 (1969).
8. Cha, S. Tight-binding inhibitors-I. Kinetic behavior. *Biochem Pharmacol* **24**, 2177-2185 (1975).
9. Revelou, P.-K. & Constantinou-Kokotou, V. Preparation of synthetic auxin-amino acid conjugates. *Synthetic Communications* **49**, 1708-1712 (2019).
10. Mashiguchi, K. *et al.* The main auxin biosynthesis pathway in Arabidopsis. *Proc Natl Acad Sci U S A* **108**, 18512-18517 (2011).
11. Tanaka, K. *et al.* UGT74D1 catalyzes the glucosylation of 2-oxindole-3-acetic acid in the auxin metabolic pathway in Arabidopsis. *Plant & cell physiology* **55**, 218-228 (2014).
12. Aoi, Y. *et al.* UDP-glucosyltransferase UGT84B1 regulates the levels of indole-3-acetic acid and phenylacetic acid in Arabidopsis. *Biochemical and biophysical research communications* **532**, 244-250 (2020).
13. Kai, K., Nakamura, S., Wakasa, K. & Miyagawa, H. Facile preparation of deuterium-labeled standards of indole-3-acetic acid (IAA) and its metabolites to quantitatively analyze the disposition of exogenous IAA in Arabidopsis thaliana. *Biosci Biotechnol Biochem* **71**, 1946-1954 (2007).
14. Maiti, M., Rozenski, J., De Jonghe, S. & Herdewijn, P. Aspartic acid based nucleoside phosphoramidate prodrugs as potent inhibitors of hepatitis C virus replication. *Org Biomol Chem* **13**, 5158-5174 (2015).
15. Katritzky, A.R., Khelashvili, L. & Munawar, M.A. Syntheses of IAA- and IPA-amino acid conjugates. *J Org Chem* **73**, 9171-9173 (2008).
